# Single-molecule junctions of multinuclear organometallic wires: long-range carrier transport brought about by metal–metal interaction†‡

**DOI:** 10.1039/d0sc06613c

**Published:** 2021-02-08

**Authors:** Yuya Tanaka, Yuya Kato, Kaho Sugimoto, Reo Kawano, Tomofumi Tada, Shintaro Fujii, Manabu Kiguchi, Munetaka Akita

**Affiliations:** Laboratory for Chemistry and Life Science, Institute of Innovative Research, Tokyo Institute of Technology 4259 Nagatsuta, Midori-ku Yokohama 226-8503 Japan ytanaka@res.titech.ac.jp akitatit@icloud.com; Department of Chemical Science and Engineering, School of Materials and Chemical Technology, Tokyo Institute of Technology 4259 Nagatsuta, Midori-ku Yokohama 226-8503 Japan; Kyushu University Platform of Inter/Transdisciplinary Energy Research, Kyushu University 744 Motooka, Nishi-ku Fukuoka 819-0395 Japan; Department of Chemistry, School of Science, Tokyo Institute of Technology 2-12-1 Ookayama, Meguro-ku Tokyo 152-8551 Japan

## Abstract

Here, we report multinuclear organometallic molecular wires having (2,5-diethynylthiophene)diyl-Ru(dppe)_2_ repeating units. Despite the molecular dimensions of 2–4 nm the multinuclear wires show high conductance (up to 10^−2^ to 10^−3^*G*_0_) at the single-molecule level with small attenuation factors (*β*) as revealed by STM-break junction measurements. The high performance can be attributed to the efficient energy alignment between the Fermi level of the metal electrodes and the HOMO levels of the multinuclear molecular wires as revealed by DFT–NEGF calculations. Electrochemical and DFT studies reveal that the strong Ru–Ru interaction through the bridging ligands raises the HOMO levels to access the Fermi level, leading to high conductance and small *β* values.

## Abstract 2

Multinuclear organometallic molecular wires having (diethynylthiophene)diyl-Ru(dppe)_2_ repeating units show high conductance with small attenuation factors. The strong Ru–Ru interaction is the key for the long-range carrier transport.

## Graphical abstract

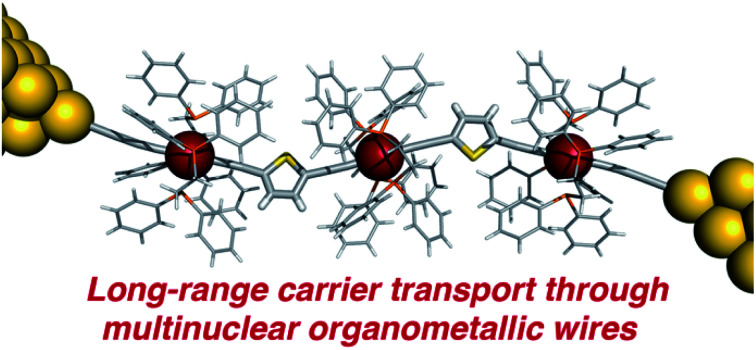

## Introduction

One of the goals of molecular devices is to control carrier transport through molecular junctions.^1–4^ Molecular conductance, however, strongly depends on the dimensions of the molecules according to the following equation: *G* = *A* exp(−*βL*), where *G*, *A*, *β*, and *L* refer to conductance, contact resistance, attenuation factor, and molecular length, respectively. To realize molecular wires with efficient long-range electron-transport performance, it is essential to minimize the *β* factor as much as possible. The coherent tunneling process dominates for molecules with small dimensions, while the incoherent hopping process additionally contributes to the conductance of molecules with dimensions greater than ∼3 nm.^5–7^ The former process often causes a large *β* factor mainly due to the energy mismatch between the molecules and electrodes.

**Fig. 1:**
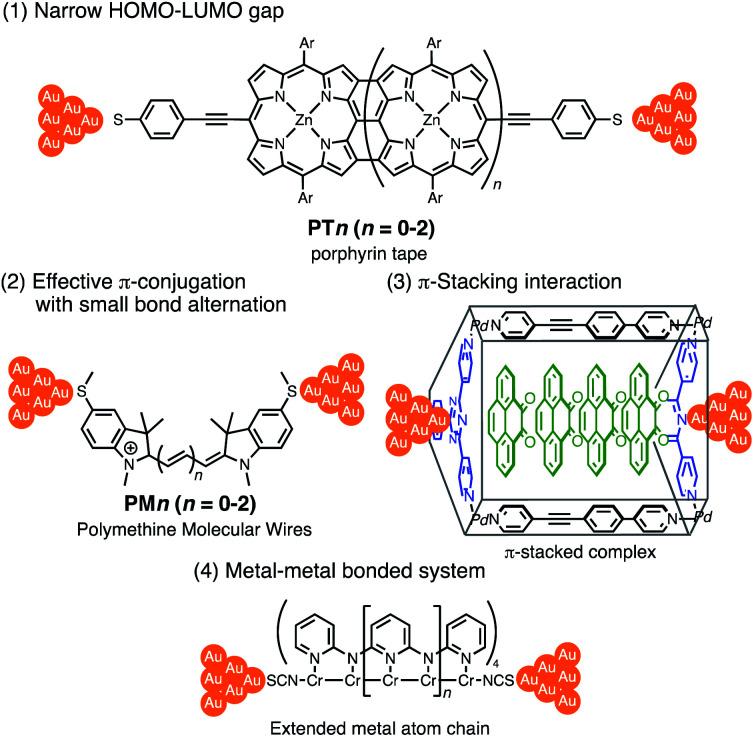
Examples of molecular junctions with small *β* values.

Thus far, extensive single-molecule conductance studies have unveiled general strategies for organic and inorganic molecular wires with small *β* values such as (1) narrowing the HOMO–LUMO gap,^8–10^ (2) extending π-conjugated systems (*e.g.* less bond alternation),^11,12^ and making use of (3) π-stacking interactions^13–15^ and (4) metal–metal bonded systems (Fig. 1).^16,17^ These strategies have led to the successful development of efficient but relatively short molecular wires (<2 nm). On the other hand, those longer than 2 nm remain to be developed due to the instability of longer derivatives and synthetic difficulty.

**Fig. 2:**
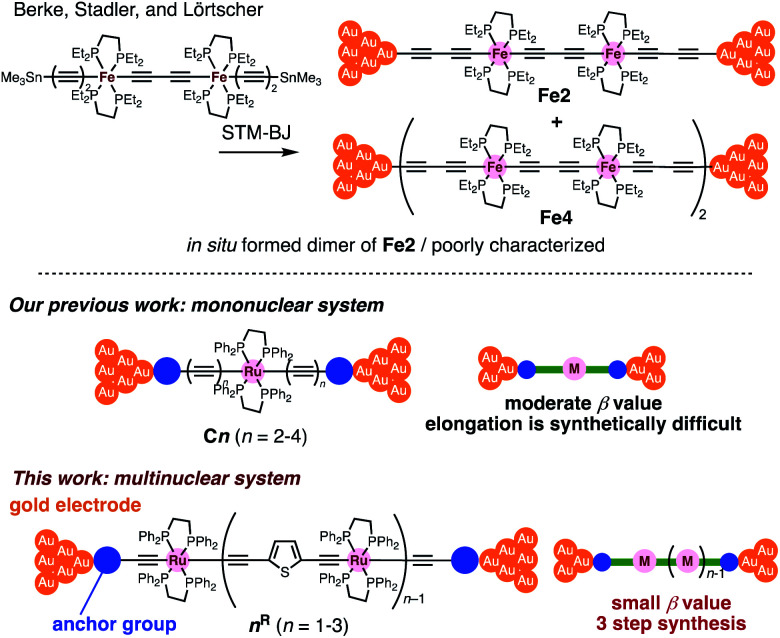
(Top) Examples of multimetallic molecular wires. (Bottom) Concept of our previous and present studies.

Insertion of metal fragments into organic molecular wires is an effective way to make the *β* factor smaller while increasing the molecular dimensions.^18,19^ Single-molecule conductance studies of multimetallic complexes containing metal fragments as repeating units are scarce^20^ in contrast to those of self-assembled monolayer systems.^21–26^ Berke, Stadler, Lörtscher *et al.* reported the single-molecule conductance of multinuclear molecular wires of a diiron complex (**Fe2**), which dimerized to form a tetrairon complex (**Fe4**) during break-junction (BJ) measurements using a scanning tunneling microscope (STM) (Fig. 2 top).^27^ In this case, however, the poor characterization of the *in situ* formed dimer **Fe4** prevented the detailed study of a series of multinuclear molecular wires.^28^

Recently, we reported metallapolyyne wires **Cn** with electron-rich Ru(dppe)_2_ fragments (dppe: 1,2-bis(diphenylphosphino)ethane) end-capped with gold groups, which facilitated covalent linkage with gold electrodes. The molecular wires shorter than 2 nm turned out to be highly conductive (10^−2^ to 10^−3^*G*_0_, 1 *G*_0_ = 77.5 μS).^29,30^ The synthetic difficulty, however, hampered the elongation of the system and the *β* value was moderate (0.25 Å^−1^). To address these issues, we designed a series of mono-, di- and tri-nuclear molecular wires **nR** (*n* = 1–3) consisting of (2,5-diethynylthiophene)diyl-Ru(dppe)_2_ repeating units (Fig. 2 bottom and 3). To examine the effect of the terminal anchor groups, pyridine- (**nPy**) and gold-terminated complexes (**nAu**) were prepared. Notably, these molecular wires were accessible within three steps from their known precursors and showed significantly small *β* values as a result of metal–metal interaction as will be discussed later.

## Results and discussion

### Synthesis and characterization

**Fig. 3:**
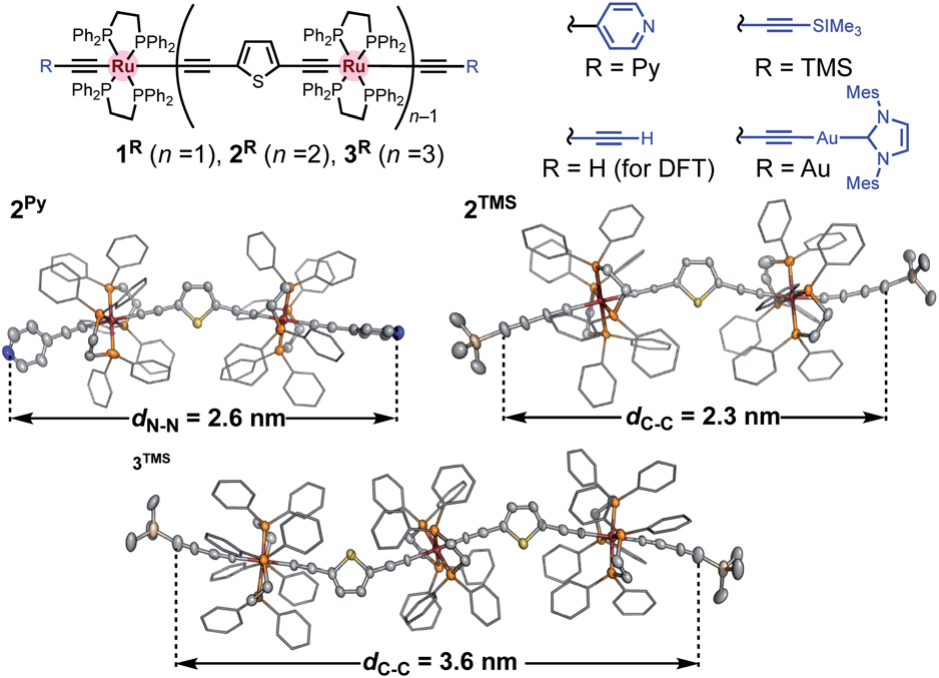
Molecular structures of **nR** (*n* = 1–3; R = Py, H, TMS, and Au) and X-ray structures of **2Py**, **2TMS**, and **3TMS**. Thermal ellipsoid plots are set at the 50% probability level. Phenyl rings are represented by a stick model. Hydrogen atoms, solvent molecules, and disordered parts are omitted for clarity.

We prepared multinuclear molecular wires **2Py** and **3Py** in two steps from the known vinylidene complex [*trans*-ClRu(dppe)_2_(C=CH_2_)][PF_6_].^31–34^ Treatment of the vinylidene complex with H–C≡C–Th–C≡C–H **4** (for **2Py**; Th: 2,5-thiophenediyl) and *trans*-(H–C≡C–Th–C≡C)_2_Ru(dppe)_2_**5** (for **3Py**) followed by Sonogashira coupling reaction with 4-bromopyridine afforded **2Py** and **3Py**, respectively. On the other hand, gold-terminated complexes **2Au** and **3Au** with N-heterocyclic carbene auxiliaries were prepared *via* sequential desilylation and auration of the TMS-protected precursors **2TMS** and **3TMS**, respectively, which were obtained from *trans*-ClRu(dppe)_2_(C≡CC≡CTMS) and **4**/**5**.^35^ We characterized these complexes by conventional spectroscopic methods. Notably, the complexes prepared here were stable under ambient conditions in both solution and solid state. Molecular structures of **2Py**, **2TMS**, and **3TMS** determined by X-ray crystallography are shown in Fig. 3. The distances between the atoms to be connected to the gold electrodes during the subsequent STM-BJ measurements are 2.6 (**2Py**; N⋯N), 2.3 (**2TMS**; C⋯C), and 3.6 nm (**3TMS**; C⋯C), and separations of the Ru atoms are around 1.2 nm. While the thiophene parts may come into contact with gold electrodes,^36^ they are sufficiently protected by the bulky dppe ligands.

### STM-break junction study

**Fig. 4:**
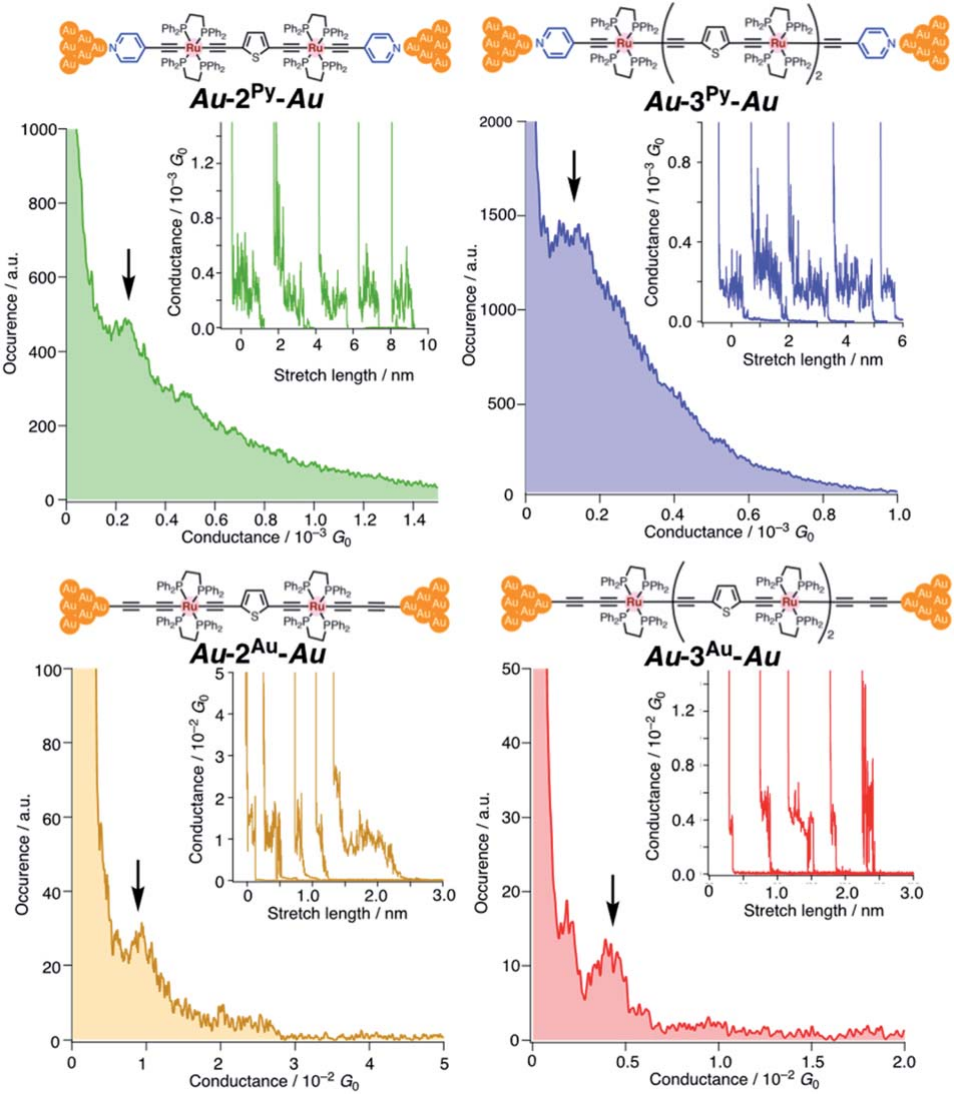
1D linear-scale histograms obtained by the STM break-junction measurements of **2R** and **3R** (R = Py and Au) of 0.1 mM tetraglyme solution. Arrows indicate molecular conductance. Insets show individual traces. No data selection was performed.

**Fig. 5:**
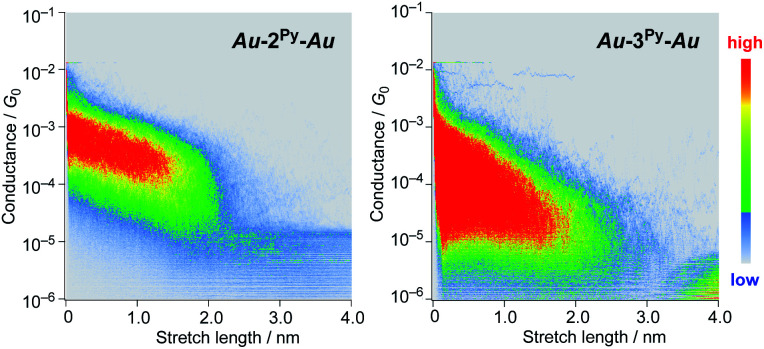
2D histograms of **2py** and **3py**.

We performed single-molecule conductance measurements of the obtained wires **nR** using the STM break-junction method (bias voltage: 100 mV).^37^ Individual traces for 0.25 M tetraglyme solutions of the pyridine-terminated complexes **2Py** and **3Py** (Fig. 4 and S11‡) showed steps with high probabilities for the formation of molecular junctions (>90%). Both of the linear and log histograms showed peaks in the 10^−4^*G*_0_ region. The 2D histograms for **2Py** and **3Py** revealed that the molecular junction formation was extended over the 2 nm region (Fig. 5), indicating junction formation at the terminal pyridine anchors rather than at the thiophene linker parts (*d*_N–S_ ∼1.3 nm). Single-molecule conductance of **2Py** and **3Py** was 2.1 (±0.6) and 1.3 (±0.6) × 10^−4^*G*_0_, respectively, as determined by statistical analysis of the linear-scale histograms.

Next, molecular wires **2Au** and **3Au** were subjected to an STM-break junction study (1.0 mM in tetraglyme, Fig. 4 and S12‡). The lack of coordinating anchor groups, like those in the pyridine-anchor in **2Py** and **3Py**, lowered the formation probability of the molecular junctions **Au–2Au–Au** and **Au–3Au–Au** through the C(acetylide)–Au(electrode) covalent bonds to 5–10% (*via e.g.* transmetalation and fusion).^35^ Nevertheless, in the individual traces, the steps were observed in the 10^−2^ to 10^−3^*G*_0_ region, and single-molecule conductance of **2Au** and **3Au** was determined to be 1.0 (±0.3) × 10^−2^ and 4.2 (±1.2) × 10^−3^*G*_0_, respectively, from the statistical analysis of the linear-scale histograms. The virtually linear relationship between the conductance in the logarithmic scale and the junctions' dimensions suggests the occurrence of the tunneling process for molecular junctions of **1R–3R** (R = Py, Au).^38,39^ Thus, the *β* values for **1Py–3Py** and **1Au–3Au** are determined to be 0.03 and 0.07 Å^−1^, respectively.

**Fig. 6:**
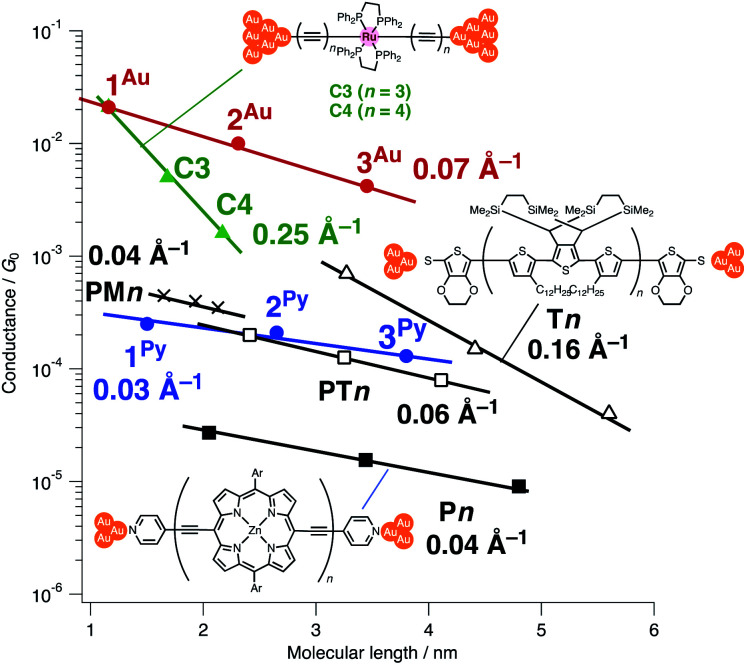
Plots of conductance against the molecular lengths and *β* values for various molecular wires (measured with bias voltages below 200 mV). The molecular lengths are the distances between the terminal anchor atoms of the model complexes as estimated from the PM6 calculation.

**Fig. 7:**
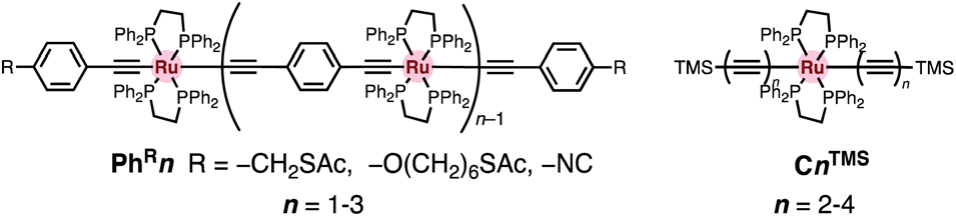
Molecular structures of the related 1,4-diethynylbenzene-bridged wire with the Ru(dppe)_2_ fragments **PhRn** and metallapolyynes with trimethylsilyl end groups **CnTMS**.

When compared with the representative molecular wires with low *β* values obtained with low bias voltage (<200 mV) (Fig. 6),^40^ the obtained *β* value turns out to be significantly smaller than that for the mononuclear polyyne wires **Cn** (0.25 Å^−1^) and comparable to or even smaller than those for porphyrin wires **Pn** (0.04 Å^−1^)^8^ and porphyrin tapes **PTn** (0.06 Å^−1^).^9^ Furthermore, the *β* value for **1R–3R** is smaller than those of the related 1,4-diethynylbenzene-bridged wire with Ru(dppe)_2_ fragments **PhRn** (0.09–0.16 Å^−1^, Fig. 7) estimated by conductive probe AFM measurements of the self-assembled monolayer.^22,23^ Two factors are considered for the smaller *β* factor for **1R–3R**. One is that thienylene linkers were reported to support metal–metal interactions compared to phenylene linkers.^41^ The other is the terminal anchor groups: **PhRn** bear electronically weakly coupling thioacetate ester and cyanide groups. Details are still unclear, and the terminal anchor groups were also reported to affect the *β* values.^42^

The excellent performance of **nAu** can be ascribed to the two following factors: (1) the small contact resistance of the covalent C(acetylide)–Au electrode bonds and (2) the performance of the bridging linkers. For the former factor, the absolute conductance of the organometallic wires **1Au–3Au** is significantly larger than that for the molecules compared herein due to the small contact resistance brought about by the strong coupling between the acetylide anchors and the gold electrodes as discussed previously by Lörtscher, Venkatesan, Berke, *et al.*^43^ For the bridging linkers, when compared with the porphyrin wires **Pn** with the same pyridine anchor groups,^8^ the conductance of **1Py–3Py** is larger by one order of magnitude. Furthermore, the conductance of **1Py–3Py** is comparable to that of the wires with the less resistive thiolate and thioether anchor groups^44^ such as the porphyrin tapes **PTn**, polymethine wires **PMn**,^11^ and oligo(thiophene) wires **Tn**.^6,45^ Thus, wires **nAu** are superior to the related wires in these two aspects.

### DFT–NEGF study

According to the approximated Landauer–Büttiker formula, zero bias conductance (*G*) can be expressed by eqn (1),^46,47^1
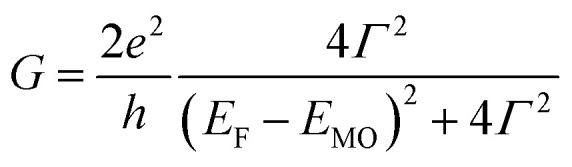
where *e*, *h*, *Γ*, *E*_F_ and *E*_MO_ represent elemental electric charge, the Planck constant, the electronic coupling between the electrodes and the molecule, the Fermi energy level of the electrodes, and the energy levels of the frontier orbitals of the molecule, respectively. Generally, the *β* value decreases with the increase of the dimensions of the molecular wire because the contribution of the terminal anchor groups in the conduction orbitals decreases as compared to that of the bridging linker.^48^ In other words, conductance decreases as the molecular wire is lengthened, if *E*_MO_ stays constant. To suppress the conductance decay, therefore, controlling *E*_MO_ is essential.

**Fig. 8:**
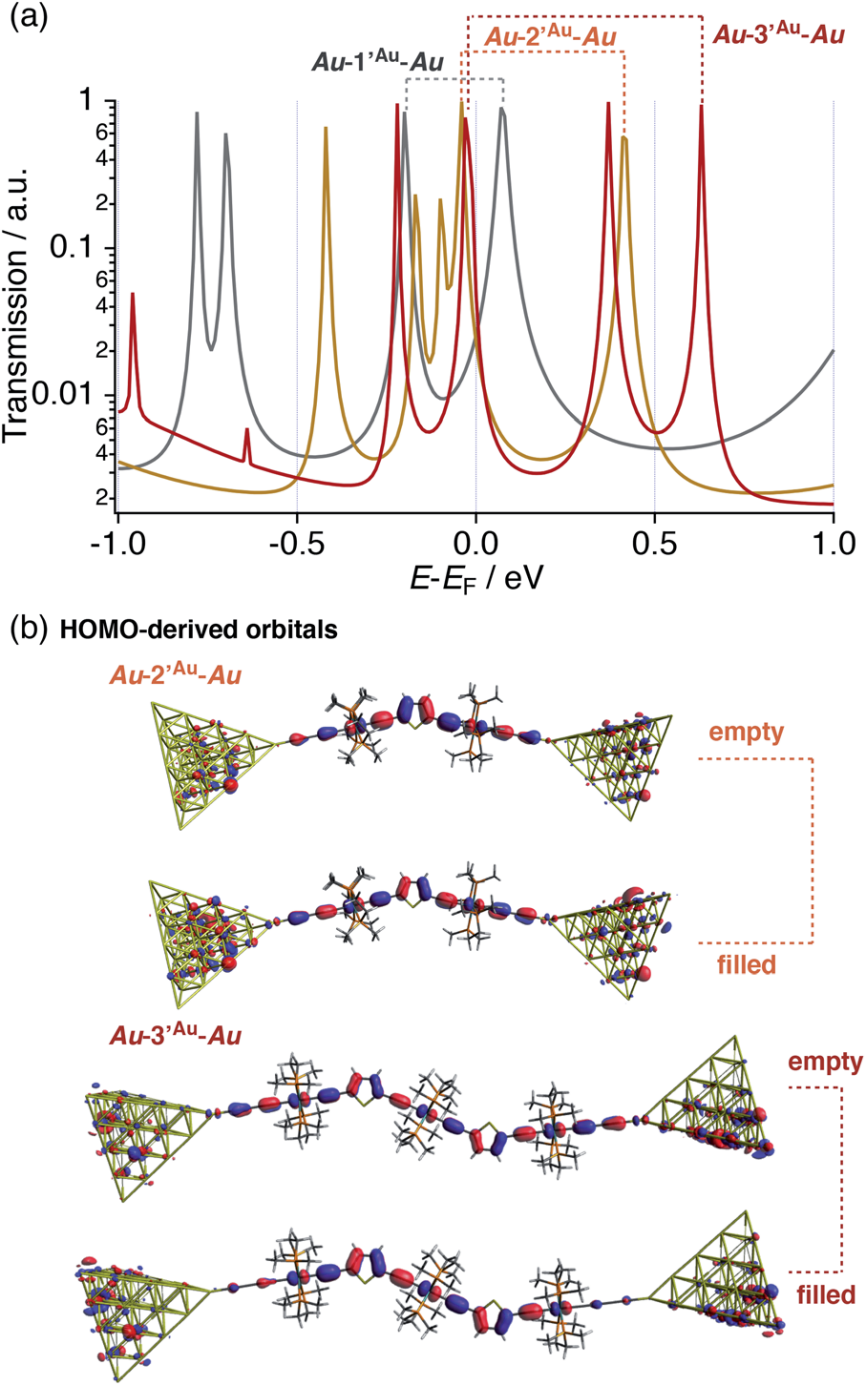
(a) Transmission spectra of **Au–n′Au–Au** (*n* = 1–3). Dotted lines: pairs of split conduction peaks derived from the HOMOs of the corresponding metal complexes. (b) Conduction orbitals for **Au–2′Au–Au** and **Au–3′Au–Au** derived from the HOMOs of the corresponding metal complexes.

In this respect, DFT–NEGF calculations were carried out in order to gain insights into the highly conductive nature and small *β* value of the multinuclear molecular junctions **Au**–**nAu**–**Au**.^49^ The truncated model complexes, where the Ph groups of the dppe ligands were replaced with Me groups for the sake of calculation cost, were used as molecular junction models (denoted as **1′Au–3′Au**). Because acetylide anchor groups form stable σ bonds with Au electrodes,^50^ the on-top σ-attachment to Au_35_ pyramidal clusters has been adopted as the molecular junction models. Transmission spectra are shown in Fig. 8a. As we reported previously,^29^ the metallapolyyne junction **Au–1Au–Au** shows a unique transmission spectrum with the two peaks closely located above and below the Fermi level (*E*_F_) of the electrodes (highlighted with the dashed line). This phenomenon is due to the splitting of the HOMO of the **1Au** caused upon molecular junction formation as a result of charge transfer interaction between the Ru center and the electrodes through the acetylide anchor groups.^51^ In a manner similar to the dppe junction **Au–1Au–Au**, the truncated dmpe analogues **Au–n′Au–Au** (*n* = 1–3) also undergo orbital splitting (Fig. 8a) to generate the filled and empty conduction peaks located within 0.5 eV from *E*_F_, and these conduction peaks originate from the HOMO and HOMO−1 orbitals of the corresponding molecules **n′H** (*n* = 1–3, Fig. 8b).^52^ The orbital characteristics for the HOMO of **n′H** and the conduction orbitals of **Au–n′Au–Au** are almost the same (Fig. S19‡). The empty conduction orbitals are pushed up and away from *E*_F_ as the number (*n*) of repeating units increases (**Au–n′Au–Au**; 0.07 (*n* = 1) → 0.41 (*n* = 2) → 0.63 eV (*n* = 3)), while the filled conduction orbitals get closer to *E*_F_ (−0.20 → −0.05 → −0.03 eV) (Fig. 8a). In particular, the latter factor predominantly contributes to the large transmission factors of **Au–n′Au–Au** at *E*_F_ and, as a result, the theoretically estimated *β* value becomes small (0.02 Å^−1^ for **1′Au–3′Au**). The pyridine derivatives show similar features (*β* = 0.01 Å^−1^ for **1′Py–3′Py**, Fig. S23‡).^53^ These features are in stark contrast to those of the mononuclear series (**C2** (= **1Au**), **C3** and **C4**).^29^ In this case, the energies of the transmission peaks (*E*_filled_, Table 1) are rather insensitive to the dimensions of the acetylene linkers, leading to the moderate theoretically estimated *β* value (0.14 Å^−1^).

**Table 1:** Electrochemical and theoretical data for **nTMS** (*n* = 1–3) and **CnTMS** (*n* = 2, 3, 4)

Complex	In (mV)						*K*_C1_ b	*K*_C2_ b	In (eV)			
	*E*_onset_	*E*_1/2_^1^	*E*_1/2_^2^	*E*_1/2_^3^	Δ*E*_1_^a^	Δ*E*_2_^a^			HOMO_CV_^c^	HOMO_DFT_^d^ (for terminal H analogues)	*E*_filled_ e	*E*_empty_ e
**1TMS**/**C2TMS**^f^	80	150							−5.18	−4.49	−0.20^g^/−0.23^h^	0.07^g^/0.09 h
**2TMS**	−430	−360	10		370		1.8 × 10^6^		−4.67	−3.96	−0.05^g^	0.41^g^
**3TMS**	−510	−440	−200	100	240	300	1.2 × 10^4^	1.2 × 10^5^	−4.59	−3.75	−0.03^g^	0.63^g^
**C3TMS** f	195	270							−5.30	−4.59	−0.22^h^	0.09^h^
**C4TMS** f	253	325							−5.35	−4.70	−0.10^h^	0.15^h^

a Δ*E*_*n*_ = *E*_1/2_^*n*+1^ − *E*_1/2_^*n*^.

b *K*_C*n*_ = exp(Δ*E*_*n*_(*V*)·*F*/*RT*).

c HOMO_CV_ = –(*E*_onset_/*V* + 5.1) eV.

d DFT calculations performed for the terminal H analogues at the B3LYP/LanL2DZ, 6-31G(d) levels of theory.

e Conduction orbital energies estimated using the DFT–NEGF method.

f Ref. 25.

g Calculations were performed for the truncated dmpe models.

h Calculations were performed for the dppe models.

### Metal–metal interaction

**Fig. 9:**
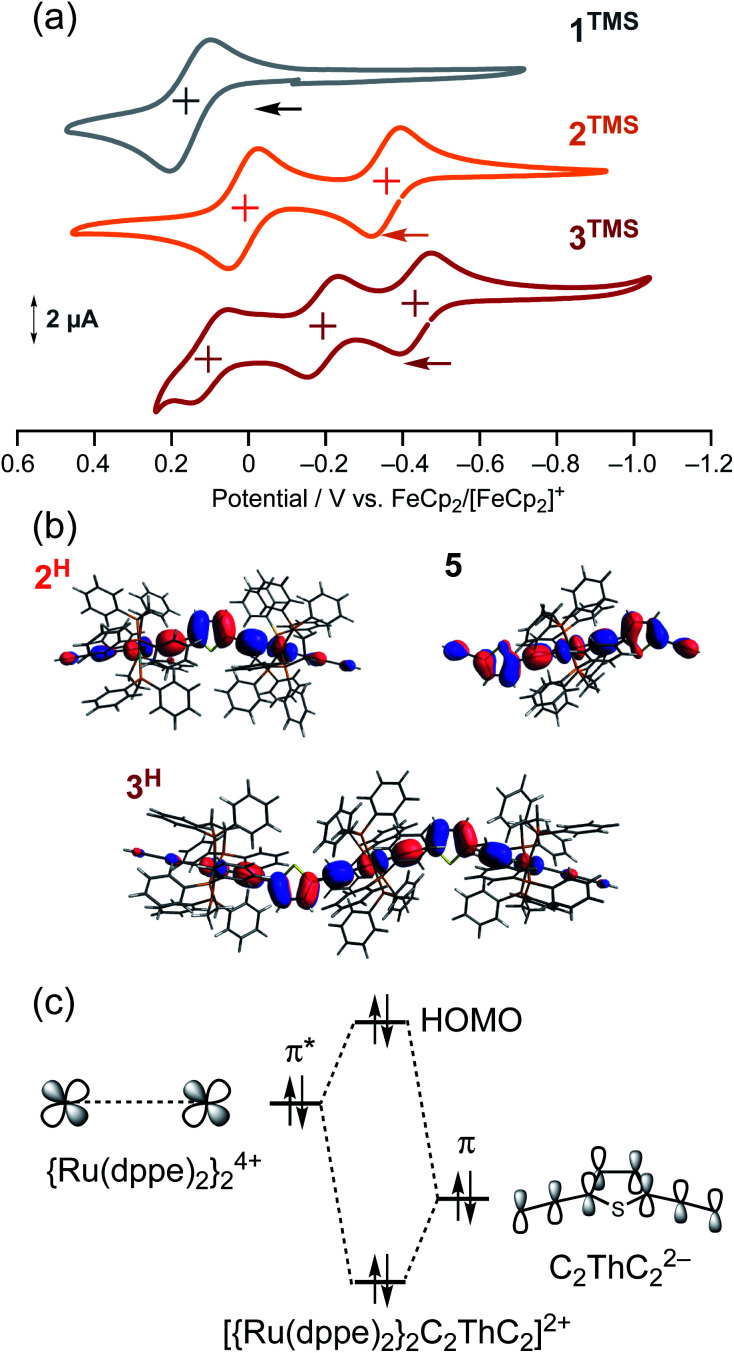
(a) Cyclic voltammograms of **1TMS–3TMS**. (b) HOMO orbitals of **2H**, **3H** and **5**. (c) Qualitative orbital interaction diagram of {Ru(dppe)_2_}_2_^4+^ and C_2_ThC_2_^2−^.

To gain further insight into the energy shift of the conduction orbitals of the multinuclear systems (**nAu**, *n* = 1–3), we carried out electrochemical and DFT studies for the TMS (**nTMS**) and H analogues (**nH**), respectively (Fig. 9 and Table 1). While the mononuclear complex **1TMS** shows a single redox wave, the di- (**2TMS**) and tri-nuclear complexes (**3TMS**), respectively, show successive two and three reversible oxidation waves.^54^ The large separations (Δ*E*_*n*_ = *E*_1/2_^*n*^ − *E*_1/2_^*n*−1^; 370 mV for the dinuclear complex **2TMS**; 240 (Δ*E*_1_) and 300 mV (Δ*E*_2_) for the trinuclear complex **3TMS**) reveal significant Ru–Ru interaction with large comproportionation constants (*K*_C_ > 10^4^).^32,55^ This metal–metal interaction makes the HOMO energies higher than that of the mononuclear wire **1TMS** (−5.18 eV) by 0.49 eV (**2TMS**) and 0.61 eV (**3TMS**). In contrast, the HOMO energy level of the mononuclear system is slightly lowered, as the acetylene linkers are elongated from **C2TMS** to **C3TMS** and **C4TMS** (Fig. 7) by 0.12 and 0.18 eV, respectively.^29^ Thus, the significant higher-energy shifts of the HOMO level are the key for the energy level alignment of the molecular junction.

To gain insight into the high-lying HOMO energies for the multinuclear wires, we carried out a DFT study.^56^ Two factors can be considered for this observation: extension of the π-conjugated systems and/or metal–metal interactions. For the multinuclear complexes **2H** and **3H**, the HOMOs are delocalized over the conjugated systems (Fig. 9b), leading to higher HOMO energy levels compared to that of **1H** (−4.49 eV; Δ*E*(HOMO_DFT_) = 0.53 eV for **2H** and 0.74 eV for **3H**) (Table 1). Furthermore, the *E*(HOMO)_DFT_ level for *trans*-(H–C≡C–Th–C≡C)_2_Ru(dppe)_2_**5** (−4.39 eV) is comparable to that of **1H** and much lower than that of **3H** (−3.75 eV). Because the HOMO of **5**, a substructure of **3H**, is highly delocalized over the (C≡C–Th–C≡C)_2_Ru moiety as also observed for **3H** (Fig. 9b), the extension of the π-system and substituent effects on the *E*(HOMO_DFT_) level for **3H** are negligible. The HOMOs of **2H** and **3H** are composed of the π*-orbitals of the ruthenium fragments and the π-orbitals of the diethynylthienylene bridging linkers, and the anti-bonding orbital interaction between them raises the HOMO energy levels (Fig. 9c).^41^ Thus, the high-lying HOMOs for **2H** and **3H**, which are caused by the metal–metal interactions through the diethynylthiophene-diyl linkers push up both of the filled and empty conduction orbitals' energies for the molecular junction, leading to effective energy alignment between the filled conduction orbitals and the Fermi energy.

## Conclusions

In summary, we have described the synthesis, and the results of single-molecule conductance measurements, and electrochemical and theoretical study of multinuclear molecular wires and junctions with the (2,5-diethynylthiophene)diyl-Ru(dppe)_2_ repeating units, **Au**–C≡C–C≡C–Ru(dppe)_2_–{C≡C–Th–C≡C–Ru(dppe)_2_}_*n*−1_–C≡C–C≡C–**Au** (*n* = 1–3). Their high single-molecule conductance with small *β* factors is shown. The DFT–NEGF study reveals that elongation by insertion of the organometallic repeating units causes a shift of the HOMO conduction peaks towards the electrode's Fermi level. The high HOMO energy levels of the multinuclear systems were ascribed to the Ru–Ru interaction as revealed by the CV and DFT study. Further study to unveil the relationship between the metal–metal interaction and single-molecule conductance is ongoing in our laboratory.

## Conflicts of interest

There are no conflicts to declare.

## Supplementary Material

SC-012-D0SC06613C-s001

SC-012-D0SC06613C-s002
