# Supplementary material for: Single-molecule junctions of multinuclear organometallic wires: long-range carrier transport brought about by metal–metal interaction
Source: Chem Sci. 2021 Feb 8;12(12):4338–44. doi: 10.1039/d0sc06613c (PMC8179486; doi:10.1039/d0sc06613c)
Supplement: SC-012-D0SC06613C-s001 [file SC-012-D0SC06613C-s001.pdf]

Electronic Supplementary Material (ESI) for Chemical Science.
This journal is © The Royal Society of Chemistry 2021

Supplementary Information

Single-Molecule Junction of Multinuclear Organometallic Wires: Long-Range Carrier Transport Brought About by Meta-Metal Interaction

Yuya Tanaka,*[a,b] Yuya Kato,[a,b] Kaho Sugimoto,[a,b] Reo Kawano,[a,b] Tomofumi Tada,[c] Shintaro Fujii,[d] Manabu Kiguchi,[d] and Munetaka Akita*[a,b]

[a] *Laboratory for Chemistry and Life Science, Institute of Innovative Research, Tokyo Institute of Technology, 4259, Nagatsuta, Midori-ku, Yokohama 226-8503, Japan.*

[b] *Department of Chemical Science and Engineering, School of Materials and Chemical Technology, Tokyo Institute of Technology, 4259,*
Nagatsuta, Midori-ku, Yokohama 226-8503, Japan.

[c] *Kyushu University Platform of Inter/Transdisciplinary Energy Research, Kyushu University, 744 Motooka, Nishi-ku, Fukuoka 819-0395, Japan*

[d] *Department of Chemistry, School of Science, Tokyo Institute of Technology, 2-12-1 Ookayama, Meguro-ku, Tokyo 152-8551, Japan*

E-mails: ytanaka@res.titech.ac.jp, akitatit@icloud.com,

Table of Contents

I. General **S4**

II. Synthesis
Scheme S1. General synthetic route for **2^R^** and **3^R^** (R = Py and Au). **S5**
Figure S1a. ^{1}H NMR spectrum of $\mathbf{2^{C2}}$ (400 MHz, C_6D_6, r.t.). **S6**
Figure S1b. ^{31}P NMR spectrum of $\mathbf{2^{C2}}$ (162 MHz, C_6D_6, r.t.). **S7**
Figure S1c. HR-ESI-TOF-MS spectrum of $\mathbf{2^{C2}}$ (CH_2Cl_2). **S7**
Figure S2a. ^{1}H NMR spectrum of $\mathbf{2^{Py}}$ (500 MHz, CD_2Cl_2, r.t.). **S9**
Figure S2b. ^{31}P NMR spectrum of $\mathbf{2^{Py}}$ (202 MHz, CD_2Cl_2, r.t.). **S9**
Figure S2c. ^{13}C NMR spectrum of $\mathbf{2^{Py}}$ (126 MHz, CD_2Cl_2, r.t.). **S10**
Figure S2d. A part of HSQC spectrum of $\mathbf{2^{Py}}$ (400 MHz, CD_2Cl_2, r.t.). **S10**
Figure S2e. HR-ESI-TOF-MS spectra of $\mathbf{2^{Py}}$ (CH_2Cl_2). **S11**
Figure S3a. ^{1}H NMR spectrum of $\mathbf{3^{H}}$ (400 MHz, C_6D_6, r.t.). **S12**
Figure S3b. ^{31}P NMR spectrum of $\mathbf{3^{H}}$ (162 MHz, C_6D_6, r.t.). **S13**

Figure S4a. ^{1}H NMR spectrum of $\mathbf{3^{Py}}$ (500 MHz, CD_2Cl_2, r.t.). S14
Figure S4b. ^{1}H NMR spectrum of $\mathbf{3^{Py}}$ (202 MHz, CD_2Cl_2, r.t.). S15
Figure S4c. A part of HH-COSY spectrum of $\mathbf{3^{Py}}$ (400 MHz, CD_2Cl_2, r.t.). S15
Figure S4d. HR-ESI-TOF-MS spectra of $\mathbf{3^{Py}}$ (CH_2Cl_2). S16
Figure S5a. ^{1}H NMR spectrum of $\mathbf{2^{TMS}}$ (400MHz, $CDCl_3$, r.t.). S17
Figure S5b. ^{31}P NMR spectrum of $\mathbf{2^{TMS}}$ (162MHz, $CDCl_3$, r.t.). S18
Figure S5c. ^{13}C NMR spectrum of $\mathbf{2^{TMS}}$ (126MHz, $CDCl_3$, r.t.). S18
Figure S5d. A part of HSQC spectrum of $\mathbf{2^{TMS}}$ (400MHz, $CDCl_3$, r.t.). S19
Figure S5e. HR-ESI-TOF-MS spectra of $\mathbf{2^{TMS}}$ **(**CH_2Cl_2). S19
Figure S6a. ^{1}H NMR spectrum of $\mathbf{2^{H}}$ (400MHz, $CDCl_3$, r.t.). S20
Figure S6b. ^{31}P NMR spectrum of $\mathbf{2^{H}}$ (162MHz, $CDCl_3$, r.t.). S21
Figure S6c. ^{13}C NMR spectrum of $\mathbf{2^{H}}$ (126MHz, $CDCl_3$, r.t.). S21
Figure S6d. A part of HSQC spectrum of $\mathbf{2^{H}}$ (400MHz, $CDCl_3$, r.t.). S22
Figure S6e. HR-ESI-TOF-MS spectra of $\mathbf{2^{H}}$ (CH_2Cl_2). S22
Figure S7a. ^{1}H NMR spectrum of $\mathbf{2^{Au}}$ (500MHz, $CDCl_3$, r.t.). S23
Figure S7b. ^{31}P NMR spectrum of $\mathbf{2^{Au}}$ (162 MHz, $CDCl_3$, r.t.). S24
Figure S7c. ^{13}C NMR spectrum of $\mathbf{2^{Au}}$ (126MHz, $CDCl_3$, r.t.). S24
Figure S7d. A part of HSQC spectrum of $\mathbf{2^{Au}}$ (500MHz, $CDCl_3$, r.t.). S25
Figure S7e. A part of HSQC spectrum of $\mathbf{2^{Au}}$ (400MHz, $CDCl_3$, r.t.). S25
Figure S7f. HR-ESI-TOF-MS spectra of $\mathbf{2^{Au}}$ (CH_2Cl_2). S26
Figure S8a. ^{1}H NMR spectrum of $\mathbf{3^{TMS}}$ (400MHz, $CDCl_3$, r.t.). S27
Figure S8b. ^{31}P NMR spectrum of $\mathbf{3^{TMS}}$ (162MHz, $CDCl_3$, r.t.). S28
Figure S8c. HR-ESI-TOF-MS spectra of $\mathbf{3^{TMS}}$ (CH_2Cl_2). S28
Figure S9a. ^{1}H NMR spectrum of $\mathbf{3^{H}}$ (500MHz, $CDCl_3$, r.t.). S29
Figure S9b. ^{31}P NMR spectrum of $\mathbf{3^{H}}$ (202MHz, $CDCl_3$, r.t.). S30
Figure S9c. HR-ESI-TOF-MS spectra of $\mathbf{3^{H}}$ (CH_2Cl_2). S30
Figure S10a. ^{1}H NMR spectrum of $\mathbf{3^{Au}}$ (400MHz, C_6D_6, r.t.). S31
Figure S10b. ^{31}P NMR spectrum of $\mathbf{3^{Au}}$ (162MHz, C_6D_6, r.t.). S32
Figure S10c. HR-ESI-TOF-MS spectra of a mixture of $\mathbf{3^{Au}}$(CH_2Cl_2). S32

III. X-ray crystallographic data S33
Table S1. Crystal data and structure refinement for $\mathbf{2^{Py}}$, $\mathbf{2^{TMS}}$, and $\mathbf{3^{TMS}}$**.** S33

IV. STM-BJ study S34
Figure S11. 1D log histograms of ***Au-n***$^{\mathbf{Py}}$***-Au*** (*n* = 2 and 3) constructed from 2000 traces without any data selection. S35
Figure S12. 1D (top) and 2D log histograms (bottom) of a tetraglyme solution (a blank experiment) and ***Au-n***$^{\mathbf{Au}}$***-Au*** (*n* = 2 and 3) complexes in tetraglyme. The histograms were constructed from 2000 traces without any data selection. S35

V. Discussion on metal-metal interactions in solution S36

Figure S13. Cyclicvoltammograms of $\mathbf{1^{Py}}$-$\mathbf{3^{Py}}$. **S37**
Table S2. Electrochemical data for $\mathbf{1^{R}}$-$\mathbf{3^{R}}$. **S37**
Figure S14. Absorption spectra of $[\mathbf{2^{Py}}]^{+}$ and $[\mathbf{2^{Py}}]^{2+}$. **S37**
Figure S15. Absorption spectra of $[\mathbf{3^{Py}}]^{+}$, $[\mathbf{3^{Py}}]^{2+}$ and $[\mathbf{3^{Py}}]^{3+}$. **S38**
Figure S16. Absorption spectra of $[\mathbf{2^{TMS}}]^{+}$ and $[\mathbf{2^{TMS}}]^{2+}$. **S38**
Figure S17. Absorption spectra of $[\mathbf{3^{TMS}}]^{+}$, $[\mathbf{3^{TMS}}]^{2+}$ and $[\mathbf{3^{TMS}}]^{3+}$. **S38**
Table S2. Absorption spectral data of $[\mathbf{2^{Py}}]^{+}$, $[\mathbf{3^{TMS}}]^{+}$ and $[\mathbf{3^{TMS}}]^{2+}$. **S39**

VI. Theoretical study **S40**
Figure S18. Energy diagrams and forntier orbitals for $\mathbf{1^{R}}$-$\mathbf{3^{R}}$ (R = H and Py). **S40**
Figure S19. Comparisons of HOMO of ***n*'$^{\mathbf{H}}$** and the conduction orbitals of ***Au- n*'$^{\mathbf{Au}}$*-Au*** (*n* = 2, 3). **S40**
Figure S20. (a) Spin density map and spin densities, and (b) *β*-HOSO and *β*-LUSO of $[\mathbf{2^{H}}]^{+}$ **S40**
Figure S21. (a) Spin density map and spin densities, and (b) *β*-HOSO and *β*-LUSO of $[\mathbf{3^{H}}]^{+}$ **S40**
Figure S22. Selected spin orbitals of $[\mathbf{2^{H}}]^{+}$ and $[\mathbf{3^{H}}]^{+}$ related to NIR absorption bands. **S41**
Table S3. UV-Vis-NIR transition data obtained by TD-DFT calculation of $[\mathbf{2^{H}}]^{+}$ **S41**
Table S4. UV-Vis-NIR transition data obtained by TD-DFT calculation of $[\mathbf{3^{H}}]^{+}$ **S41**
Figure S23. (a) Whole and (b) expanded transmission spectra for ***Au-n*'$^{\mathbf{Py}}$*-Au*** (*n* = 1-3) obtained by the DFT-NEGF method. Conduction orbitals for (c) ***Au*-2'$^{\mathbf{Py}}$*-Au*** and (d) ***Au*-3'$^{\mathbf{Py}}$*-Au*** derived from the HOMOs of the corresponding metal complexes. **S42**
Table S5. Cartesian Coordinates of $\mathbf{2^{H}}$. **S43**
Table S6. Cartesian Coordinates of $\mathbf{3^{H}}$. **S46**
Table S7. Cartesian Coordinates of $\mathbf{2^{Py}}$. **S49**
Table S8. Cartesian Coordinates of $\mathbf{3^{Py}}$. **S53**
Table S9. Cartesian Coordinates of $[\mathbf{2^{H}}]^{+}$. **S57**
Table S10. Cartesian Coordinates of $[\mathbf{3^{H}}]^{+}$. **S59**
Tabale S11. Cartesian coordinates of **5.** **S62**

VI. References **S64**

I. General

Materials

Reactions were performed under N_2 atmosphere using standard Schlenk tube technique unless stated otherwise. THF, CH_2Cl_2, diethyl ether, pentane were purified by the Grubbs solvent system.[S1] Dry MeOH and Benzene were used without any purification. Triethylamine was pre-dried by NaOH and distilled over CaH. Ru(dppe)$_2$Cl(C≡C-C≡C-TMS),[S2] [Cl-Ru(dppe)$_2$C=CH$_2$][PF$_6$],[S3] 2,5-diethynylthiophene [S4] Ru(dppe)$_2$(C≡C-C$_4$SH$_2$C≡CH)$_2$,[S5] AuNHC(Mes)Cl [S6] were synthesized according to literature procedure. Other reagents, Silica (Kanto chemical Co Inc. Silica Gel 60N) and alumina (Merck Alminum oxide 90 standardized) were used as received.

Instruments

NMR spectra were recorded on Bruker biospin AVANCE III 400 MHz (^{1}H 400 MHz, ^{31}P 162 MHz) or Bruker biospin ASCEND-500 spectrometers (^{1}H 500 MHz, ^{13}C{^{1}H} NMR 126 MHz, ^{31}P 202 MHz). NMR Chemical shifts were referenced to the residual non-deuterated solvents (^{1}H: $CHCl_3$ δ = 7.26 ppm, C_6D_5H δ = 7.16 ppm, $CDHCl_2$ δ = 5.32 ppm, ^{13}C; $CDCl_3$ δ = 77 ppm) and H_3PO_3 (an external reference for ^{31}P). ^{13}C{^{1}H} NMR spectra are only reported for dinuclear complexes except **2**$^{\mathbf{C2}}$ because of limited solubility of **2**$^{\mathbf{C2}}$ and trinuclear complexes in common organic solvents. Solvents for NMR measurements were dried over molecular sieves, degassed, and stored under nitrogen atomosphere. HR-ESI-TOF-MS were performed on Bruker micrOTOF II. UV–Vis and IR spectra (KBr pellets and CH_2Cl_2 solution) were obtained on a JASCO V670DS and FTIR 4200 spectrometer, respectively. Electrochemical measurements (CV and DPV) were made with a Hokuto DenkoHZ-5000 (observed in CH_2Cl_2; [complex] = *ca.* 1×10^{-3} M; [NBu_4PF_6] = 0.1 M; working electrode: Pt, counter electrode: Pt, reference electrode: Ag/$AgNO_3$; scan rates were 100 mV/s (CV). After the measurement, ferrocene (Fc) was added to the mixture and the potentials were calibrated with respect to the Fc/Fc$^+$ redox couple.

Single-Crystal X-ray Crystallography

X-ray Diffraction data was collected at 90-93 K under a cold nitrogen gas stream on Bruker model APEX2 platform-CCD X-ray diffractometer or Rigaku XtaLaB Synergy-DW X-ray diffractometer systems, using graphite-monochromated Mo-Kα radiation (λ = 0.71073 Å) or CuKα radiation (λ = 1.54184). Intensity data were collected by an ω-scan with 0.5° oscillations for each frame. Bragg spots were integrated using the ApexII program package[S7] or CrysAlis^{Pro} program package[S8]. Using Olex2,[S9] structures were solved by SHELXT[S10] and refined by SHELXL.[S11] All non-disordered non-hydrogen atoms except solvent molecules were refined with anisotropic displacement parameters. Hydrogen atoms were placed at calculated positions and refined by applying riding models. CCDC numbers 2039830-2039832 contain the supplementary crystallographic data for **2**$^{\mathbf{Py}}$, **2**$^{\mathbf{TMS}}$, and **3**$^{\mathbf{TMS}}$.

II. Synthesis

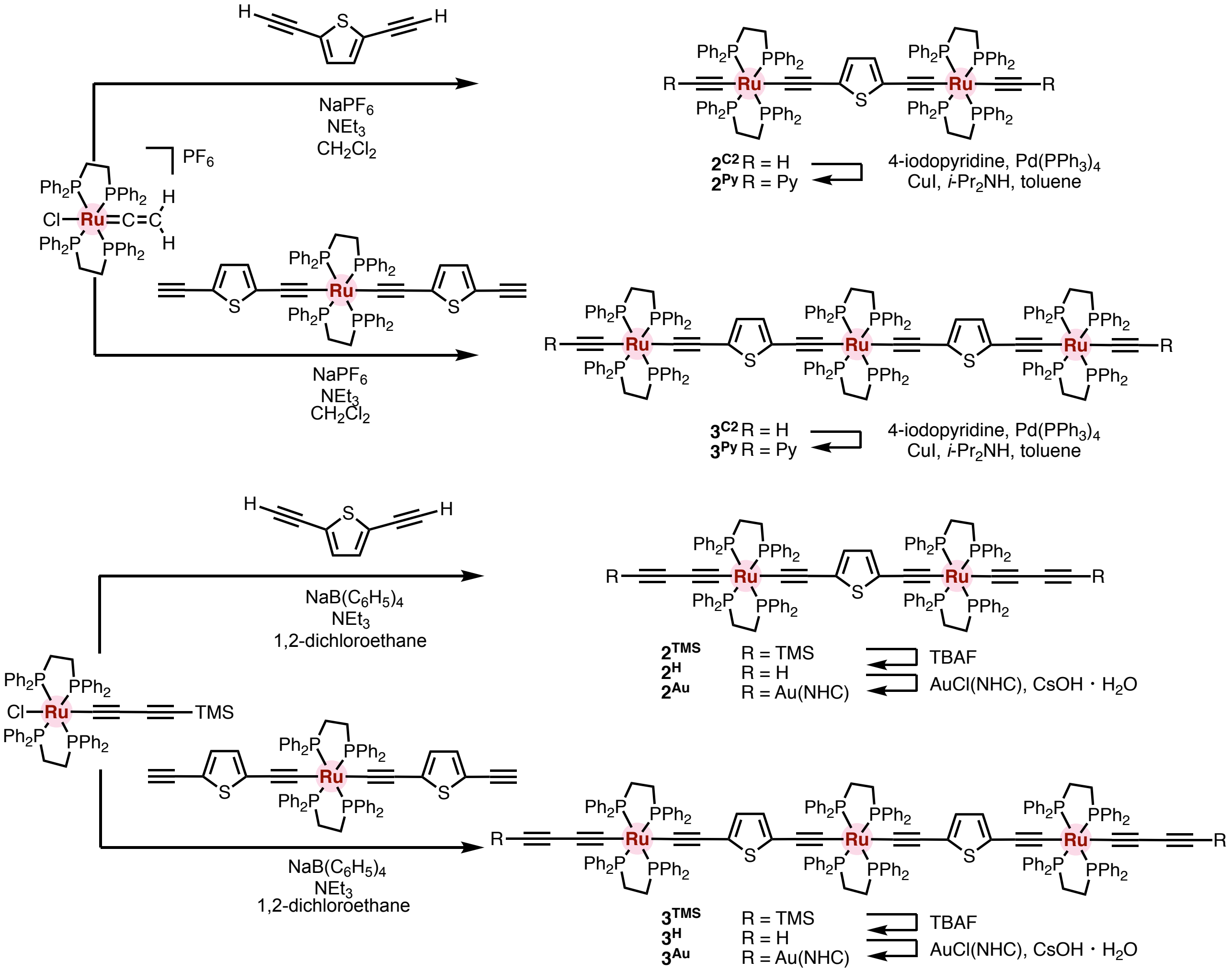

Scheme S1. General synthetic route for $\mathbf{2^R}$ and $\mathbf{3^R}$ (R = Py and Au).

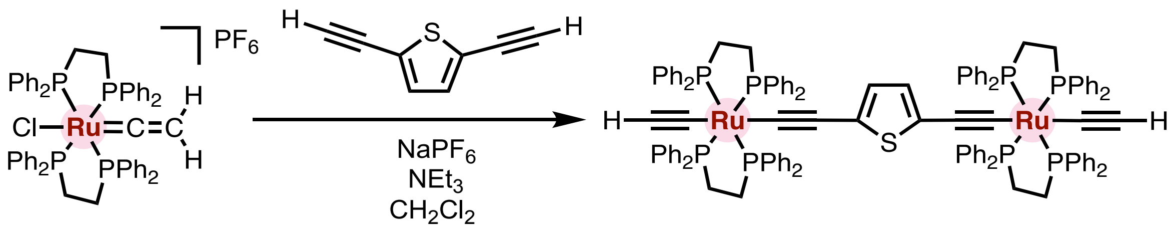

Synthesis of 2^{C2}. To a mixture of [Cl-Ru(dppe)$_2$C=CH$_2$][PF$_6$] (212 mg, 0.192 mmol) and NaPF$_6$ (64.3mg, 0.383 mmol) in CH$_2$Cl$_2$ (10 mL) was added a mixture of 2,5-diethynylthiophene (12.1 mg, 0.0914 mmol) and NEt$_3$ (1.0 mL) in CH$_2$Cl$_2$ (10 mL) *via* canula, and the resultant mixture was stirred for 24 hours at room temperature. The reaction mixture was concentrated *in vacuo* and the residue was extracted with CH$_2$Cl$_2$ and filtered through a Celite® pad. To the filtrate was added MeOH to give precipitates, which were washed with MeOH and diethyl ether, and dried under vacuum to give a yellow solid (102 mg, 0.0516 mmol, 56%).

IR (KBr, cm^{-1}): ν(≡C-H) 3270 (m), ν(C≡C) 2051 (m), 1925 (m). ^{1}H NMR (400 MHz, C$_6$D$_6$, r.t.): δ 1.99 (s, 2H, C≡C*H*), 2.65 (br, 16H, dppe-CH$_2$), 6.63 (s, 2H, C$_4$S*H*$_2$), 6.97-7.30 (m, 48H, dppe-Ph) 7.60-7.72 (m. 16H, *o*-phenyl), 7.84-7.95 (m. 16H, *o*-phenyl). ^{31}P NMR (162 MHz, C$_6$D$_6$, r.t.): δ 54.1. HR-ESI-TOF-MS (CH$_2$Cl$_2$, CsI): *m/z* Calcd. for C$_{116}$H$_{100}$P$_8$Ru$_2$S: 1976.3572, Found 1976.3561 [M]$^+$. Anal. Calcd. for C$_{116}$H$_{100}$P$_8$Ru$_2$S·(CH$_2$Cl$_2$) : C, 67.96 ; H, 4.98, Found : C, 68.18; H, 4.99.

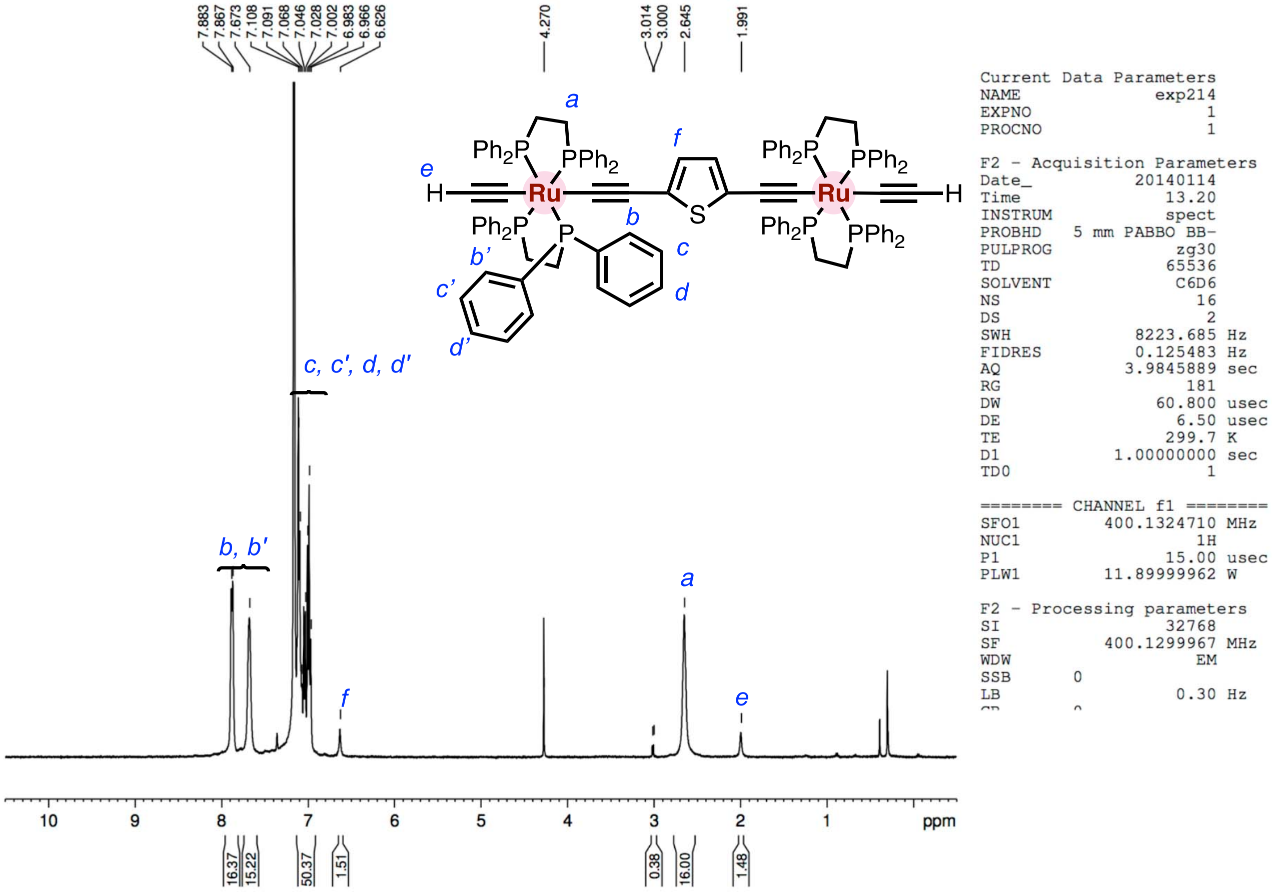

Figure S1a. ^{1}H NMR spectrum of 2^{C2} (400 MHz, C$_6$D$_6$, r.t.).

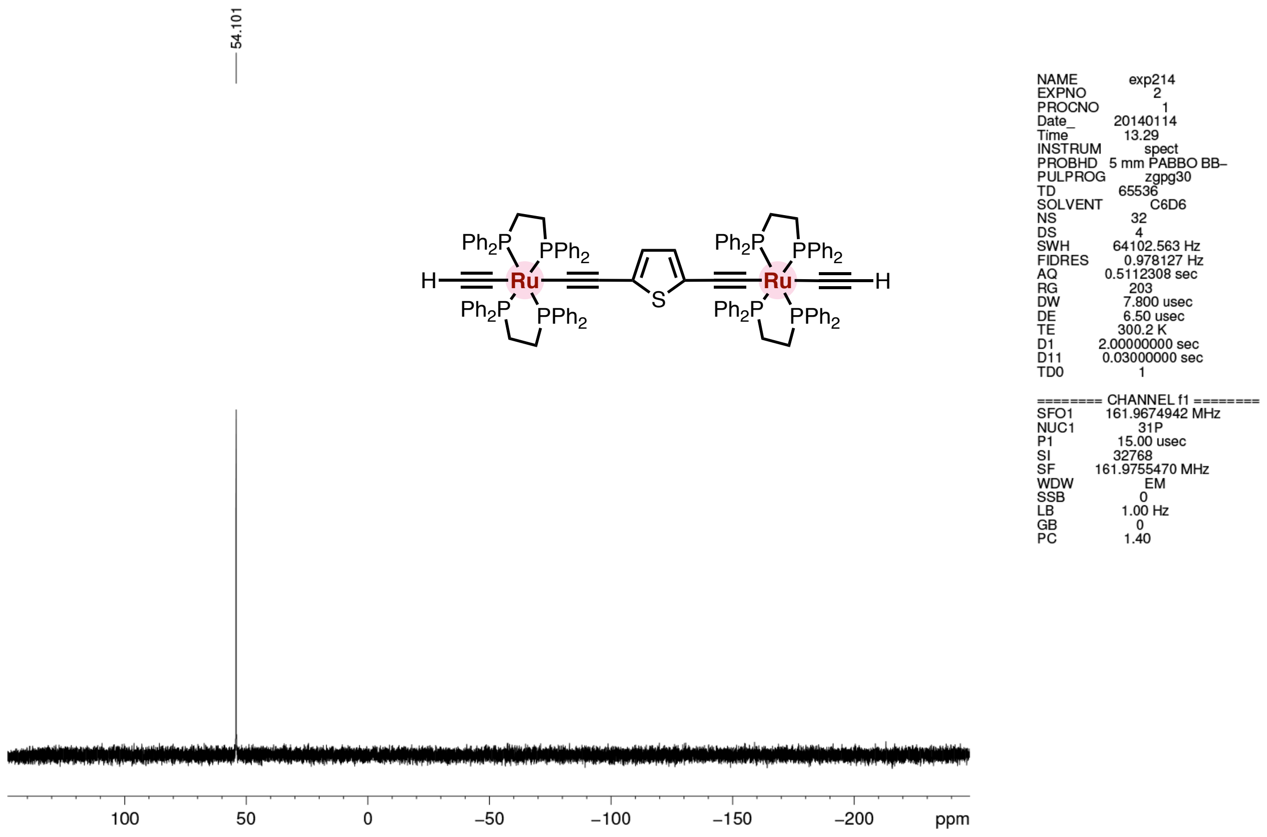

Figure S1b. ^{31}P NMR spectrum of $\mathbf{2}^{\mathbf{C2}}$ (162 MHz, C_6D_6, r.t.).

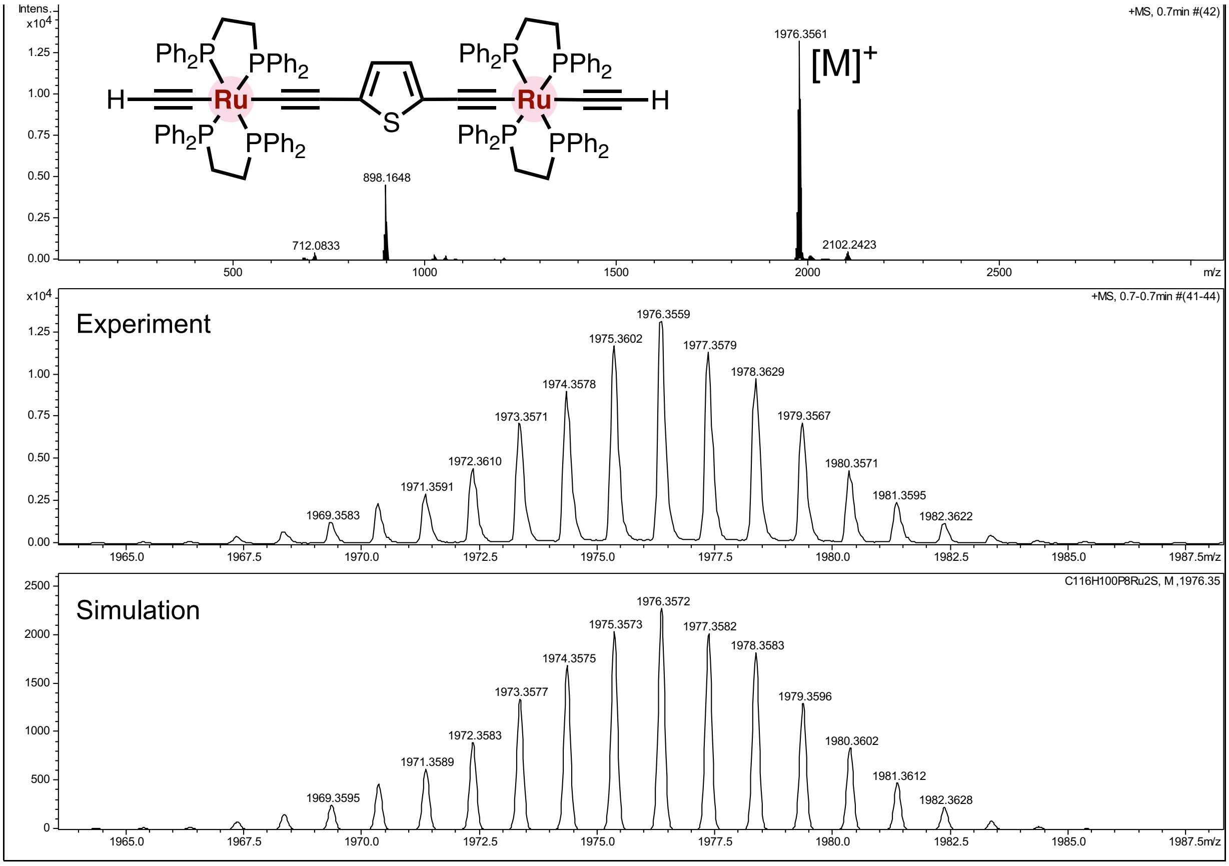

Figure S1c. HR-ESI-TOF-MS spectrum of $\mathbf{2}^{\mathbf{C2}}$ (CH_2Cl_2).

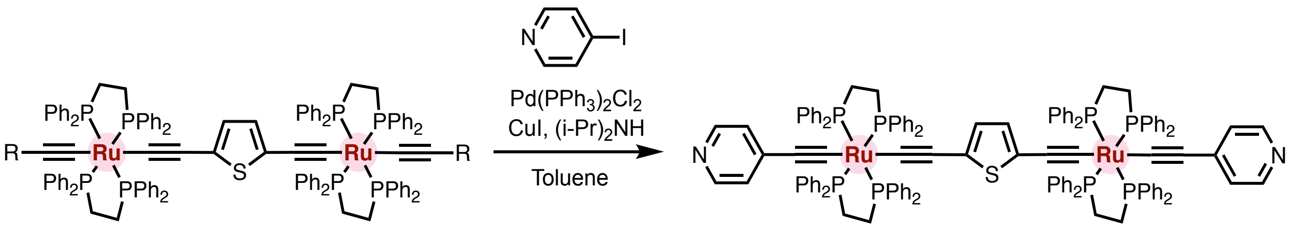

Synthesis of 2^{Py}. To a toluene solution (120 mL) of 2^{C2} (300 mg, 0.152 mmol), 4-iodopyridine (311 mg, 1.52 mmol) was added diisopropylamine (3 mL), $Pd(PPh_3)_4$ (100 mg, 0.0865 mmol) and CuI (49.0 mg, 0.258 mmol), and the mixture was stirred for 24 h at 95 ºC. To the mixture was added 4-iodopyridine (311 mg, 1.52 mmol), $Pd(PPh_3)_4$ (100 mg, 0.0865 mmol) and CuI (49.0 mg, 0.258 mmol). This process was repeated every 24h until 2^{C2} was consumed. After 5 days, triethylenetetramine (3 mL) was added to the mixture to remove copper species coordinated to the pyridine moieties. The reaction mixture was stirred for 1 h at room temperature, and then concentrated to *ca.* 15 mL, and filtered through a Celite® pad. The volatiles were removed under reduced pressure, and the residue was reprecipitated with CH_2Cl_2/diethyl ether. The collected precipitates were, then, dissolved in CH_2Cl_2, and MeOH was added to the solution to give precipitates, which were dried under reduced pressure to give an orange powder (140 mg, 0.0657 mmol, 43%).

IR (KBr, cm^{-1}): ν(C≡C) 2051 (m). ^{1}H NMR (500 MHz, C_6D_6, r.t.): δ 2.67 (br, 16H, dppe-CH_2), 6.23 (s, 2H, Th), 6.48 (d, 4H, J = 4.0 Hz, Py), 6.97 (t, 16H, J = 6.0 Hz, *m*-phenyl), 7.11 (t, 16H, J = 6.0 Hz, *m*-phenyl), 7.20 (t, 8H, J = 6.0 Hz, *p*-phenyl), 7.27 (t, 8H, J = 6.0 Hz, *p*-phenyl), 7.25–7.30 (m, 16H, *o*-phenyl), 7.32–7.38 (m, 16H, *o*-phenyl), 8.23 (d, 4H, J = 4.0 Hz, Py). ^{31}P NMR (162 MHz, C_6D_6, r.t.): δ 53.1. ^{13}C NMR (126 MHz, CD_2Cl_2, r.t.): δ 31.7, 111.2, 115.3, 124.9, 125.0, 126.9, 127.5, 127.7, 129.2, 129.3, 123.4, 134.7, 134.7 (t, J = 11.3 Hz), 137.0 (quint, J = 11.3 Hz), 137.5 (quint, J = 11.3 Hz), 137.6, 149.1. Quaternary carbon atoms of acetylene α carbon are not observed. HR-ESI-TOF-MS (CH_2Cl_2, CsI): *m/z* Calcd. for $C_{126}H_{106}N_2P_8Ru_2S$: 2130.4108, Found 2130.4106 $[M]^+$. Anal. Calcd. for $C_{126}H_{106}N_2P_8Ru_2S \cdot 2(CH_2Cl_2)$: C, 66.84 ; H, 4.82, Found : C, 66.98 ; H, 4.90.

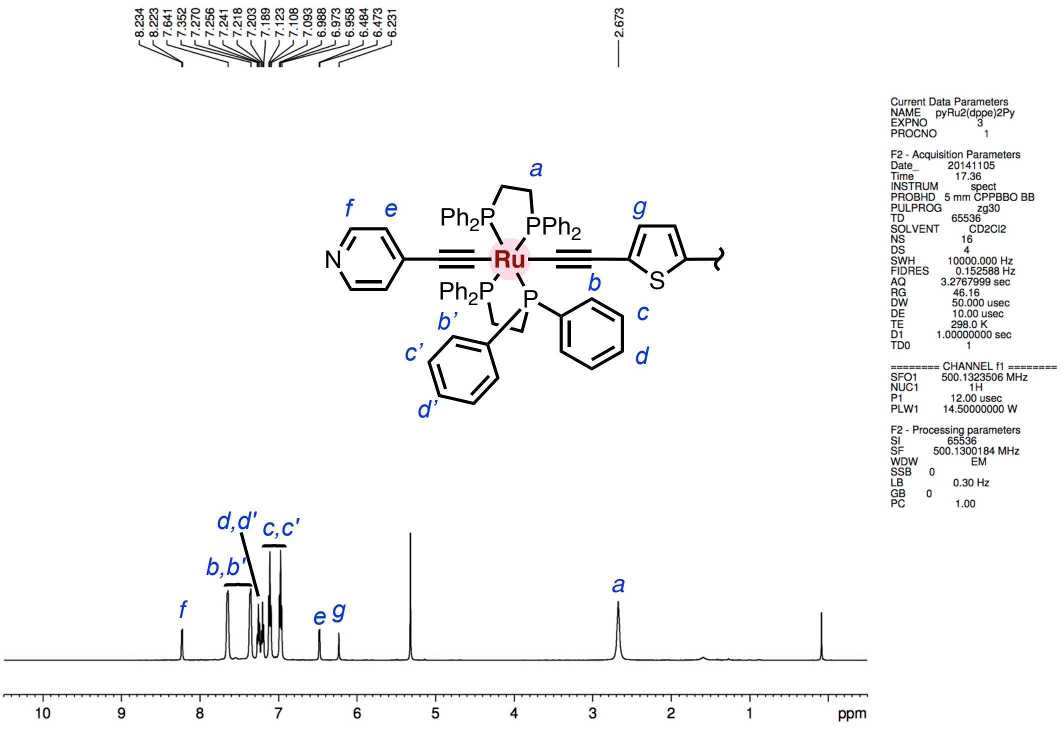

Figure S2a. ^{1}H NMR spectrum of $\mathbf{2}^{\mathbf{Py}}$ (500 MHz, CD_2Cl_2, r.t.).

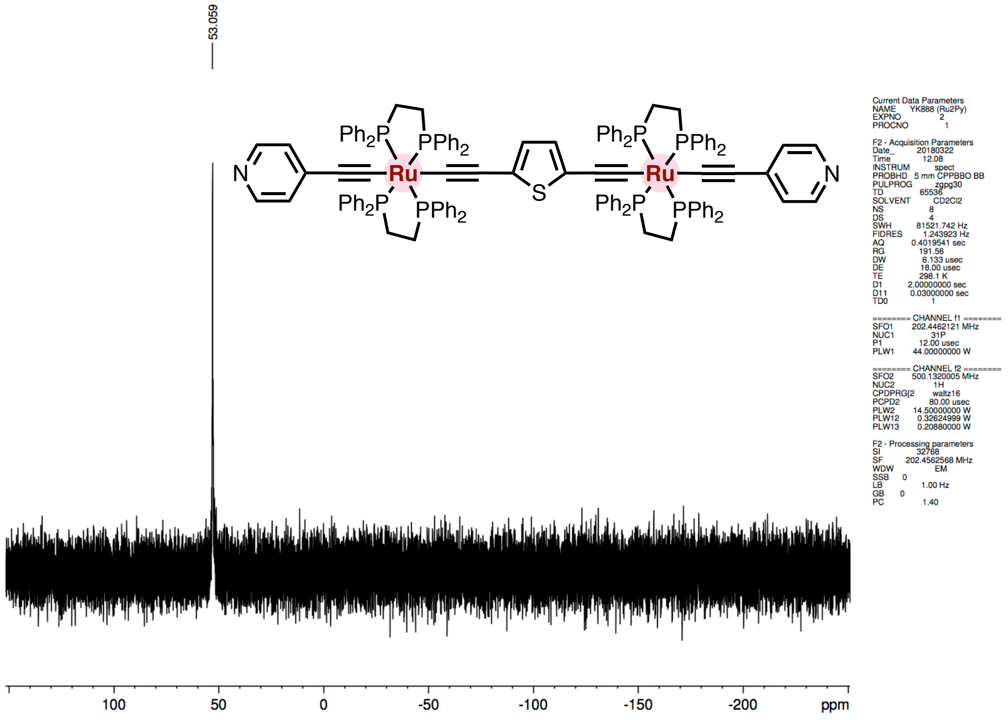

Figure S2b. ^{31}P NMR spectrum of $\mathbf{2}^{\mathbf{Py}}$ (202 MHz, CD_2Cl_2, r.t.).

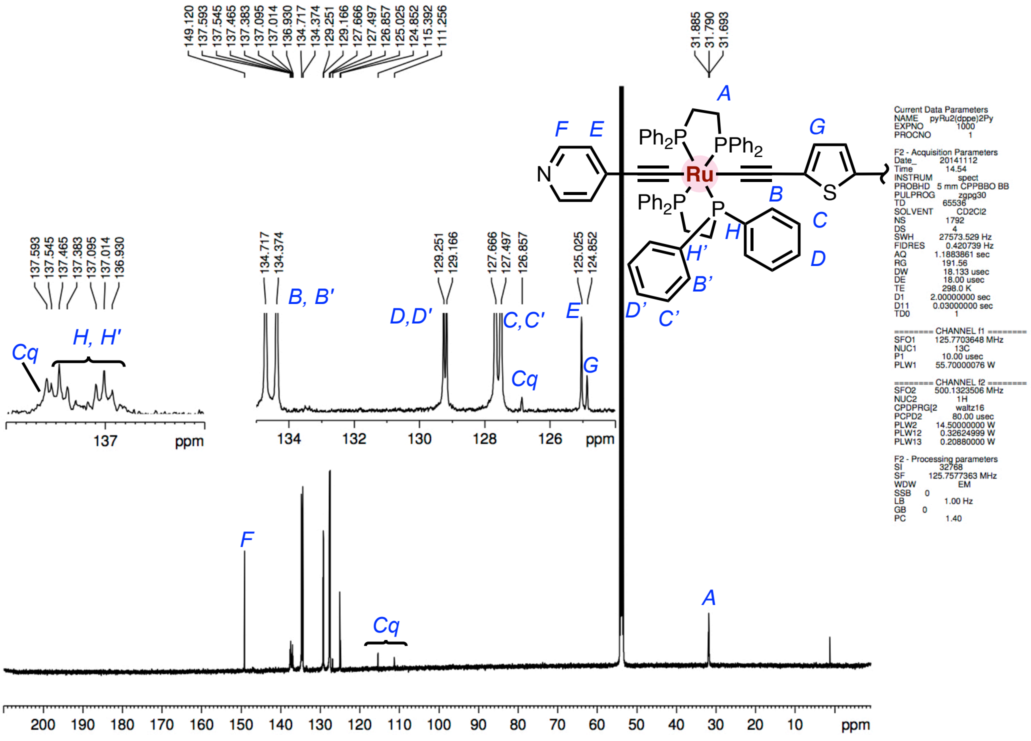

Figure S2c. ^{13}C NMR spectrum of **2**$^{\mathbf{Py}}$ (126 MHz, CD_2Cl_2, r.t.).

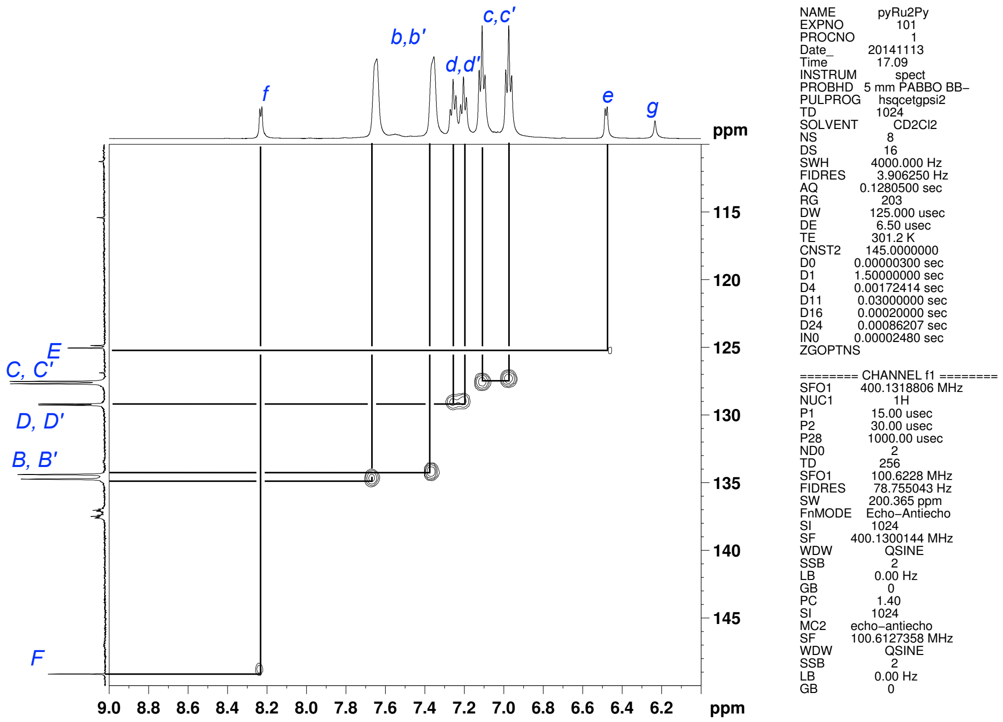

Figure S2d. A part of HSQC spectrum of **2**$^{\mathbf{Py}}$ (400 MHz, CD_2Cl_2, r.t.).

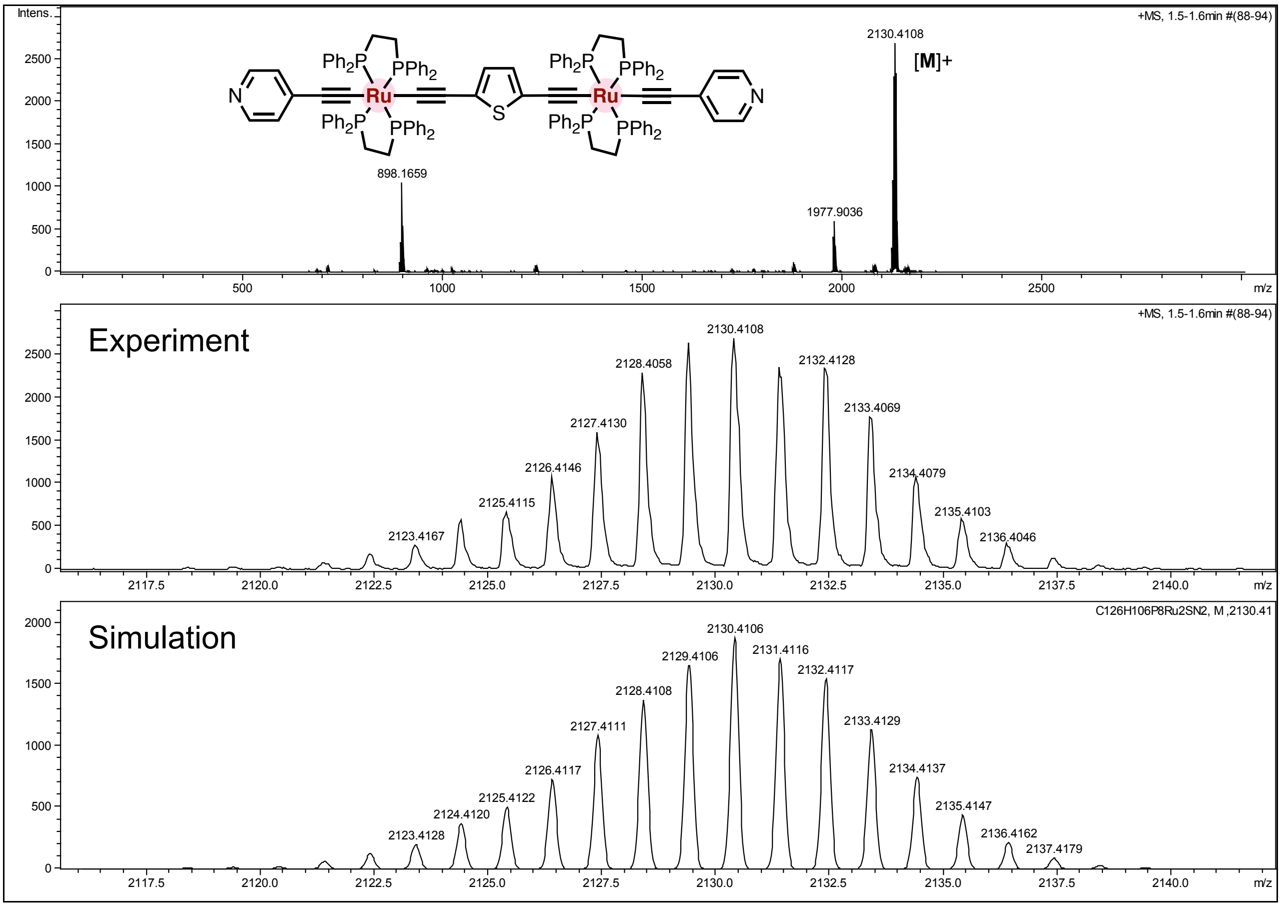

Figure S2e. HR-ESI-TOF-MS spectra of $\mathbf{2^{Py}}$ (CH_2Cl_2).

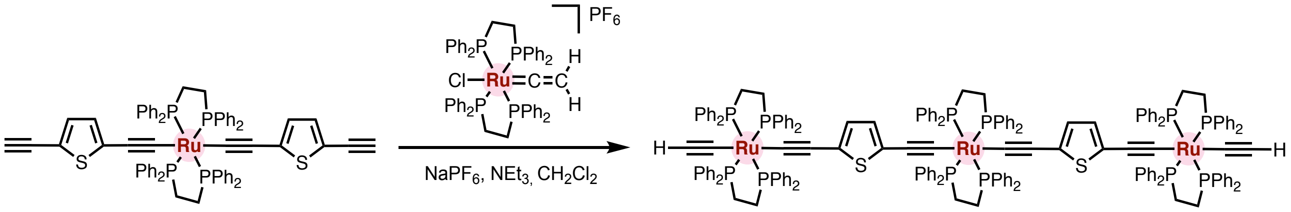

Synthesis of 3[H]. To a mixture of Ru(dppe)$_2$(C≡C-C$_4$SH$_2$C≡CH)$_2$ (186 mg, 0.160 mmol), [Cl-Ru(dppe)$_2$C=CH$_2$][PF$_6$] (442 mg, 0.400 mmol), NaPF$_6$ (107 mg, 0.640 mmol) in CH$_2$Cl$_2$ (50 mL) was NEt$_3$ (2.0 mL) and the resultant mixture was stirred for 3 hours at room temperature. The reaction mixture was concentrated *in vacuo,* and MeOH was added to the solution to give reprecipitats, which were washed with pentane and MeOH, and dried under vacuum to give a yellowish-green powder (371 mg, 0.123 mmol, 77%).

IR (KBr, cm^{-1}): ν(≡C-H) 3266 (w), ν(C≡C) 2047 (m) 1925 (w). ^{1}H NMR (400 MHz, C$_6$D$_6$, r.t.): δ 1.95 (s, 2H, C≡C*H*), 2.67 (br, 24H, dppe-CH$_2$), 6.65-6.80 (br, 4H, C$_4$S*H*$_2$), 6.97-7.20 (m, 72H, dppe-Ph), 7.40-7.70 (m, 48H, *o*-phenyl). ^{31}P NMR (162 MHz, C$_6$D$_6$, r.t.): δ 53.6, 53.8. HR-ESI-TOF-MS (CH$_2$Cl$_2$, CsI): *m/z* Calcd. for C$_{116}$H$_{100}$P$_8$Ru$_2$S : C$_{176}$H$_{150}$P$_{12}$Ru$_3$S$_2$; 3004.5224, Found 3004.5222 [M]$^+$.

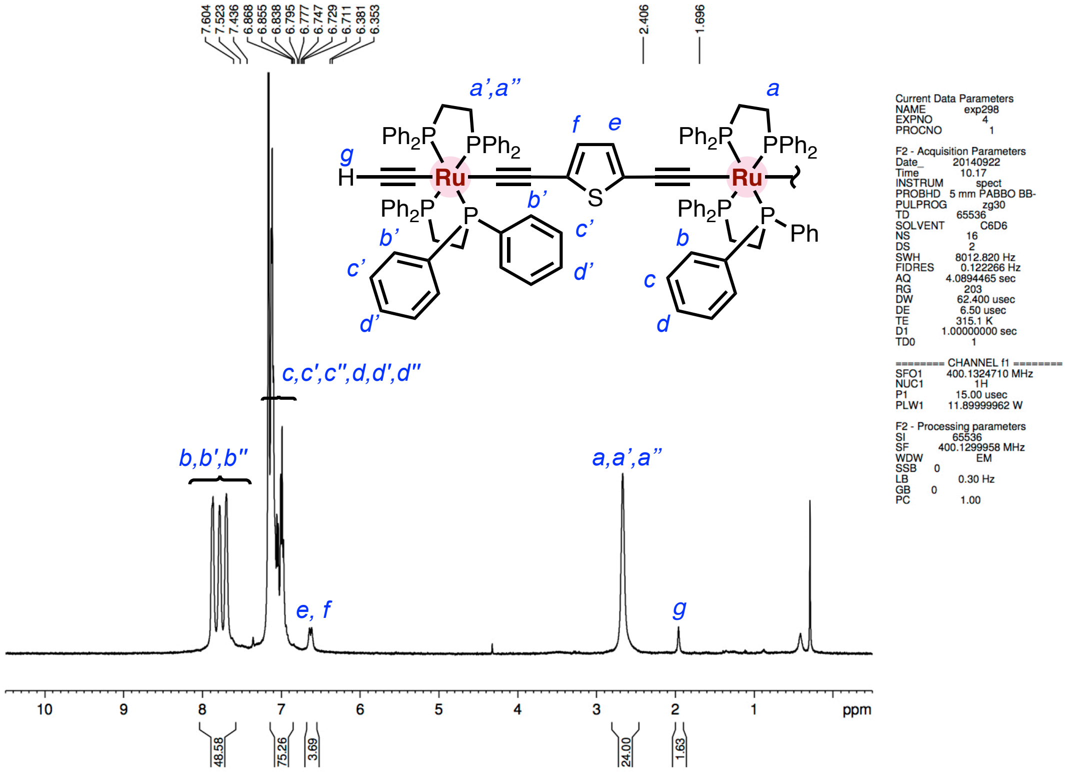

Figure S3a. ^{1}H NMR spectrum of **3^H** (400 MHz, C$_6$D$_6$, r.t.).

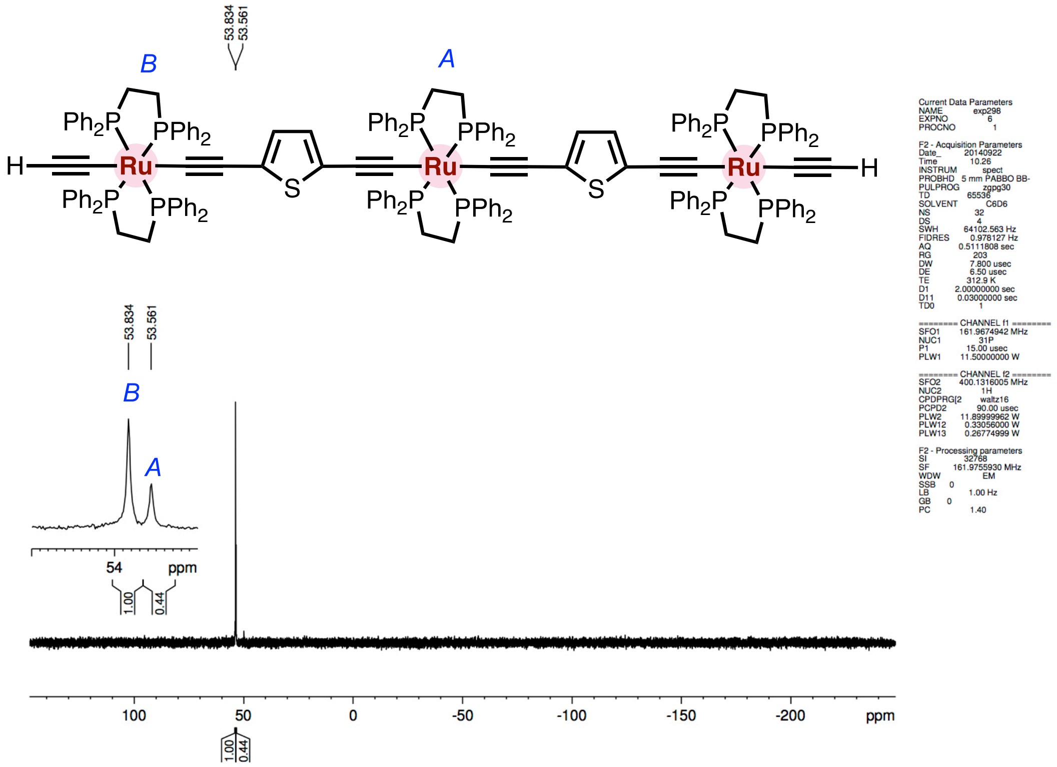

Figure S3b. ^{31}P NMR spectrum of $\mathbf{3^{H}}$ (162 MHz, C_6D_6, r.t.).

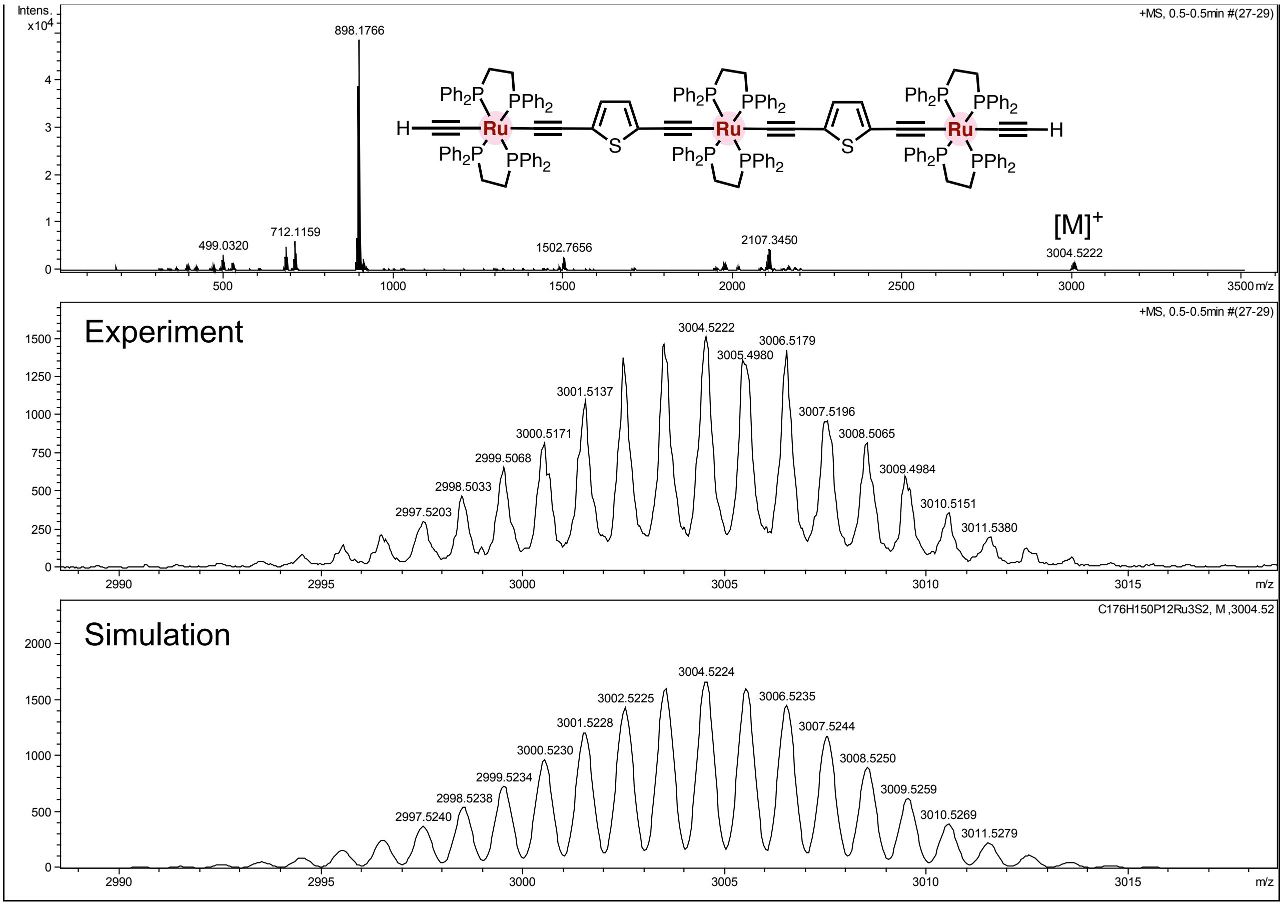

Figure S3c. HR-ESI-TOF-MS spectra of $\mathbf{3^{H}}$ (CH_2Cl_2).

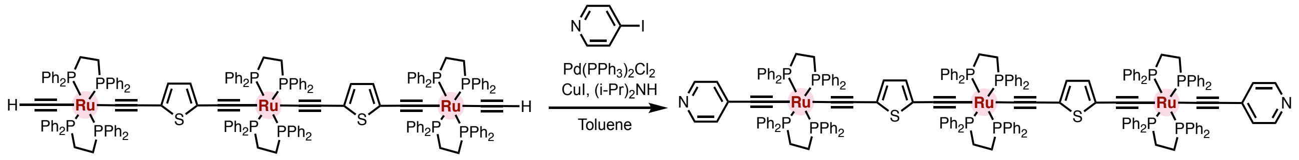

Synthesis of 3^{Py}. To a toluene solution (120 mL) of $\mathbf{3^{H}}$ (291 mg, 0.0969 mmol) and 4-iodopyridine (199 mg, 0.969 mmol) was added isopropylamine (3 mL), $Pd(PPh_3)_4$ (66.3 mg, 0.0574 mmol) and CuI (33.2 mg, 0.174 mmol), and the mixture was stirred for 24 h at 97 ºC. Additional 4-Iodopyridine (199 mg, 0.0969 mmol), $Pd(PPh_3)_4$ (66.3 mg, 0.0574 mmol) and CuI (33.2 mg, 0.174 mmol) was added and the mixture was further stirred for 24 h. Then, triethylenetetramine (3 mL) was added to the mixture, and the solution was stirred for 1 h at room temperature. The reaction mixture was concentrated to *ca.* 5 mL and filtered through a Celite® pad and the volatiles were removed under reduced pressure. The residue was washed with diethyl ether, dissolved in CH_2Cl_2. MeOH was added to the solution to give precipitates, which were dried under reduced pressure to afford a brown powder (130 mg, 0.0412 mmol, 43%).

IR (KBr, cm^{-1}): ν(C≡C) 2048 (m). ^{1}H NMR (500 MHz, CD_2Cl_2, r.t.): δ 2.67 (br, 24H, dppe-CH_2), 6.10-6.25 (br, 2H, Th), 6.39 (d, 4H, J = 5.2 Hz, Py), 6.87-7.59 (m, 120H, dppe-Ph, Th), 8.14 (d, 4H, J = 5.2 Hz, Py). ^{31}P NMR (162 MHz, CD_2Cl_2, r.t.): δ 52.8. HR-ESI-TOF-MS (CH_2Cl_2, CsI): *m/z* Calcd. for $C_{186}H_{156}N_2P_{12}Ru_3S_2$; 1579.2876, Found 1579.2879 $[M]^{2+}$. Anal. Calcd. for $C_{188}H_{160}Cl_4N_2P_{12}Ru_3S_2$·($\mathbf{3^{Py}}$•2($CH_2Cl_2$)) : C, 67.85 ; H, 4.85, Found : C, 68.10; H, 4.91.

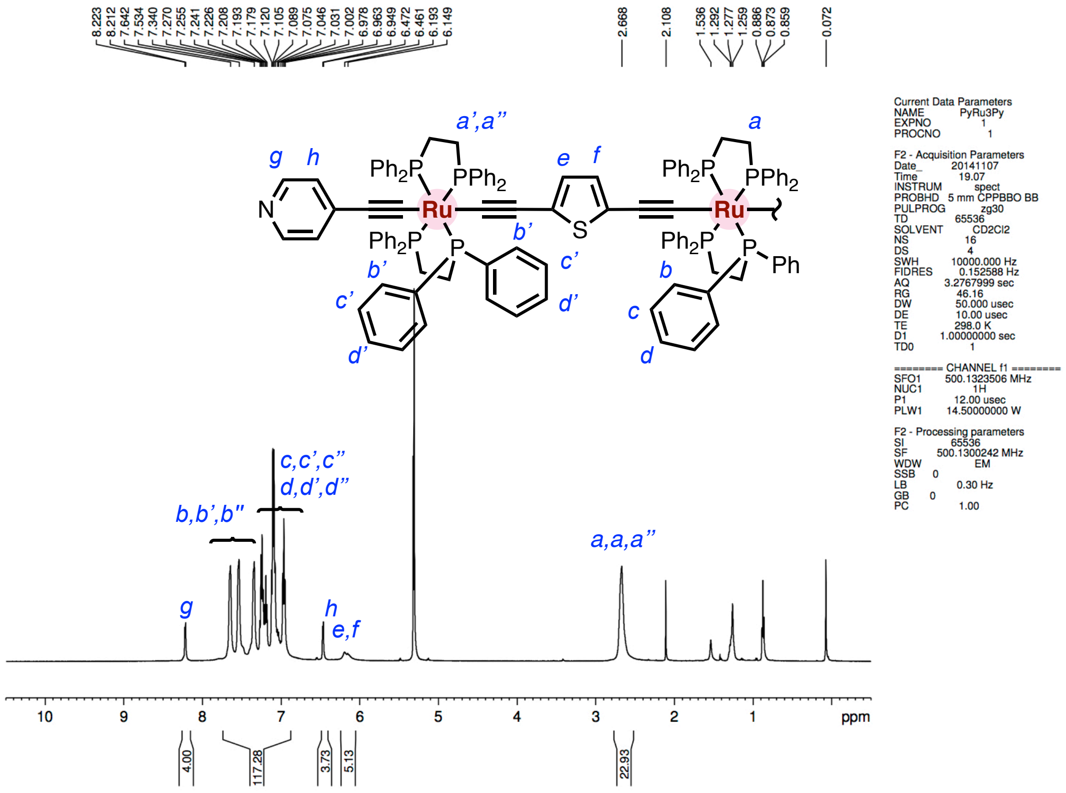

Figure S4a. ^{1}H NMR spectrum of $\mathbf{3^{Py}}$ (500 MHz, CD_2Cl_2, r.t.).

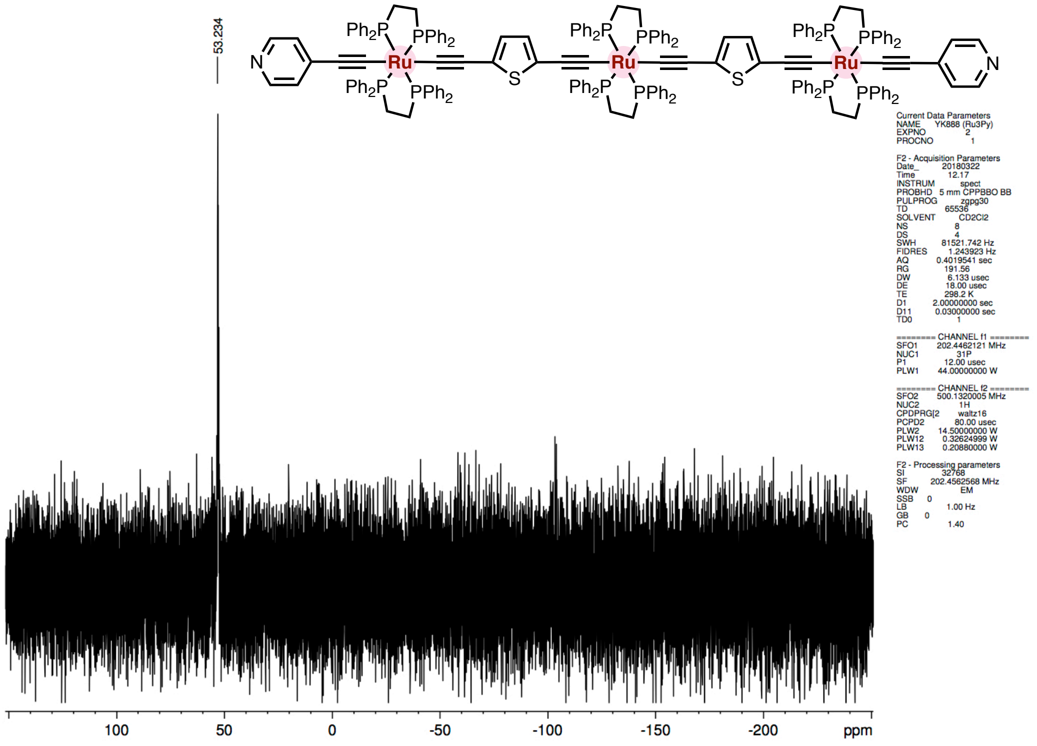

Figure S4b. ^{31}P NMR spectrum of $\mathbf{3^{Py}}$ (202 MHz, CD_2Cl_2, r.t.).

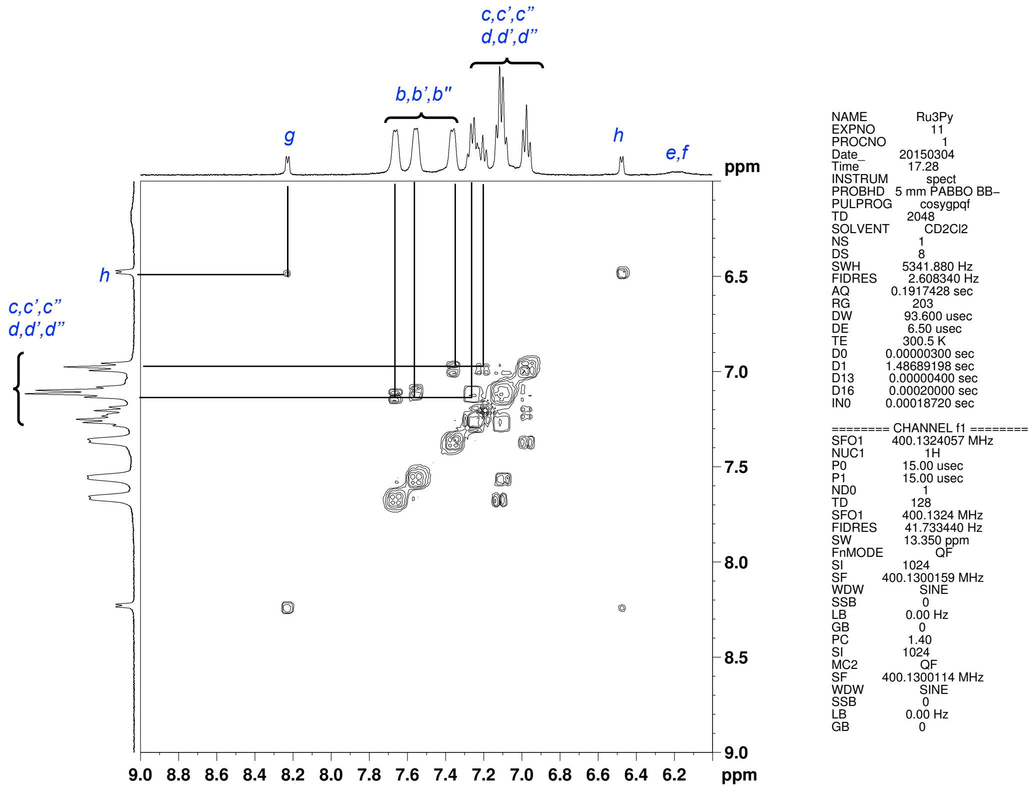

Figure S4c. A part of HH-COSY spectrum of $\mathbf{3^{Py}}$ (400 MHz, CD_2Cl_2, r.t.).

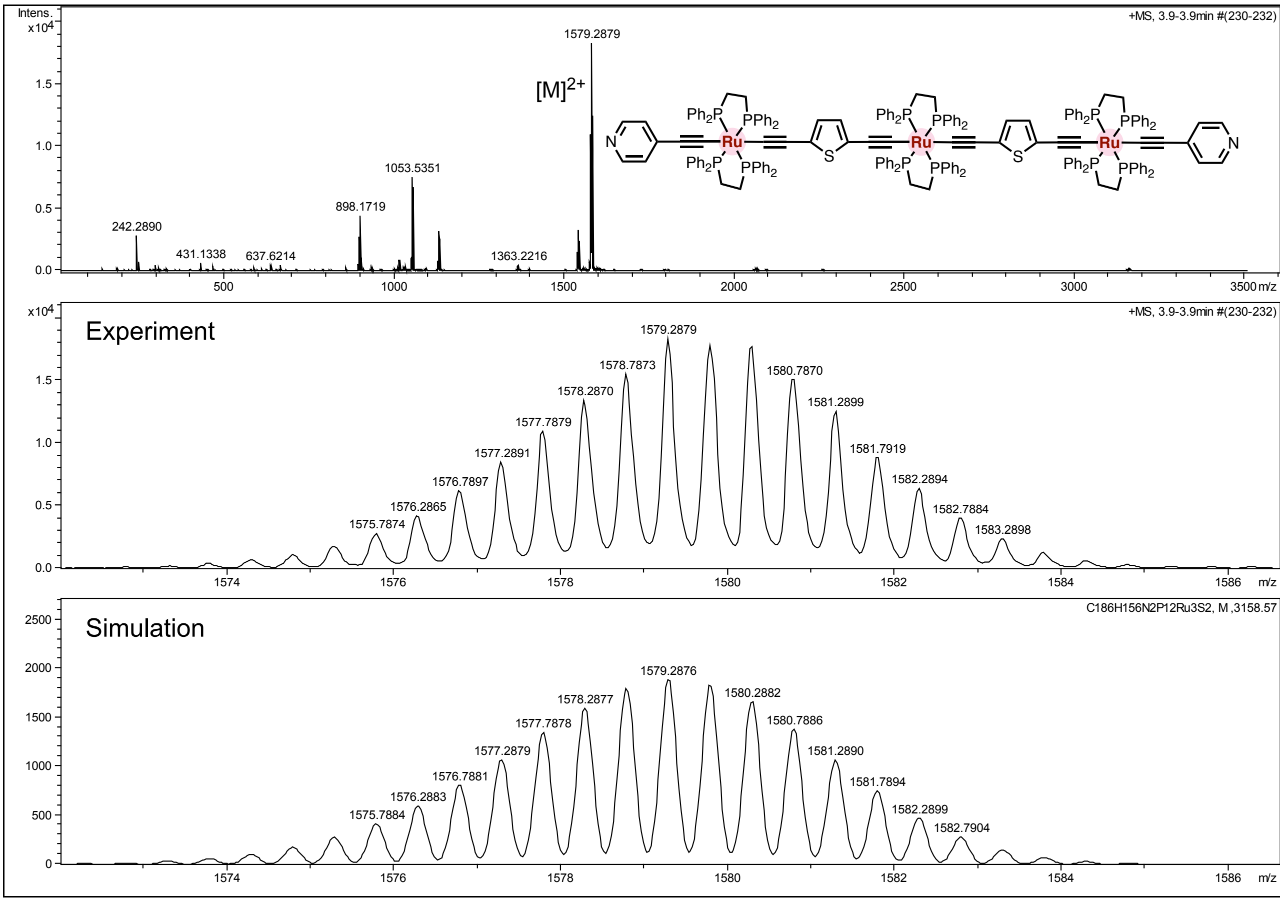

Figure S4d. HR-ESI-TOF-MS spectra of **3**$^{\mathbf{Py}}$ (CH_2Cl_2).

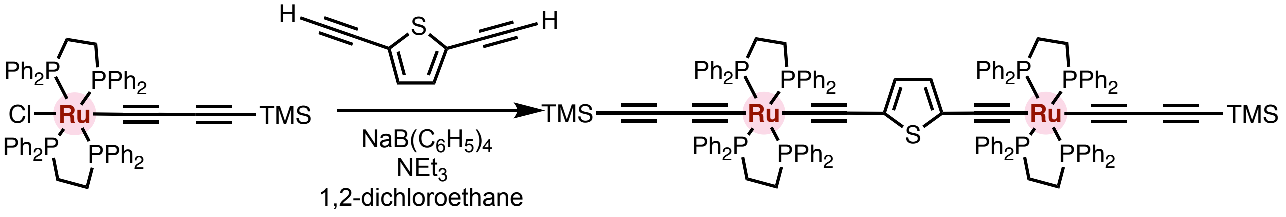

Synthesis of 2^TMS^. To a mixture of ClRu(dppe)$_2${(C≡C)$_2$TMS} (200 mg, 0.189 mmol, 2.0 eq) and $NaBPh_4$ (100 mg, 0.292 mmol, 3.0 eq) in 1,2-dichloroethane (10 mL) was added NEt_3 (1.0 mL) and 2,5-diethynylthiophene in 1,2-dichloroethane (1.8 mL, 0.05 M, 0.096 mmol, 1.0 eq), and the resultant mixture was sttirred overnight at room temperature. The reaction mixture was evaporated *in vacuo*, and the residue was washed MeOH, suspended in diethyl ether, and added hexane to give precipitates. The precipitates were washed with hexane and passed through a short alumina pad (eluted with CH_2Cl_2 / hexane = 1:1), and the filtrate was dried under vacuum to give a yellow solid (86.0 mg, 0.039 mmol, 42%).

IR (KBr, cm^{-1}): ν(C≡C) 2168 (w), 2113 (m), 2058 (m), 1996(w). ^{1}H NMR (400 MHz, $CDCl_3$, r.t.): δ 0.224 (s, 18H, TMS), 2.52–2.73 (m, 16H, dppe-CH_2), 6.15 (br, 2H, Th), 6.99-7.71 (m, 80H, dppe-Ph). ^{31}P NMR (162 MHz, $CDCl_3$, r.t.): δ 53.0. ^{13}C NMR (126 MHz, $CDCl_3$, r.t.): δ 1.12, 31.5 (*t*, *J* = 12.0 Hz, dppe-CH_2), 67.8, 95.8, 99.1, 110.9, 124.3, 126.4, 127.1, 127.2, 128.5, 128.9, 133.8, 134.5, 135.9 (quint. *J* = 10.1 Hz), 136.7 (quint. *J* = 10.1 Hz). Quaternary carbon atoms of acetylene α carbon are not observed. HR-ESI-TOF-MS (CH_2Cl_2, CsI): *m/z* Calcd. for $C_{126}H_{116}P_8Ru_2SSi_2$: 2168.4367, Found 2168.4362 [M]$^+$.

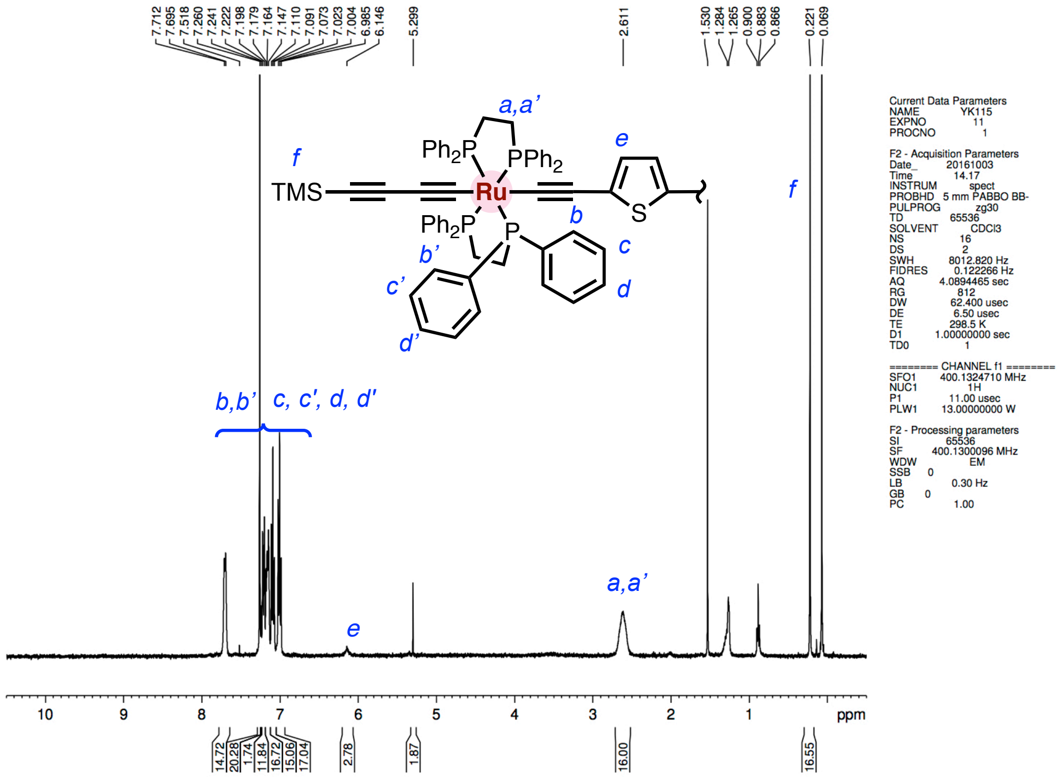

Figure S5a. ^{1}H NMR spectrum of $\mathbf{2}^{\mathbf{TMS}}$ (400MHz, $CDCl_3$, r.t.).

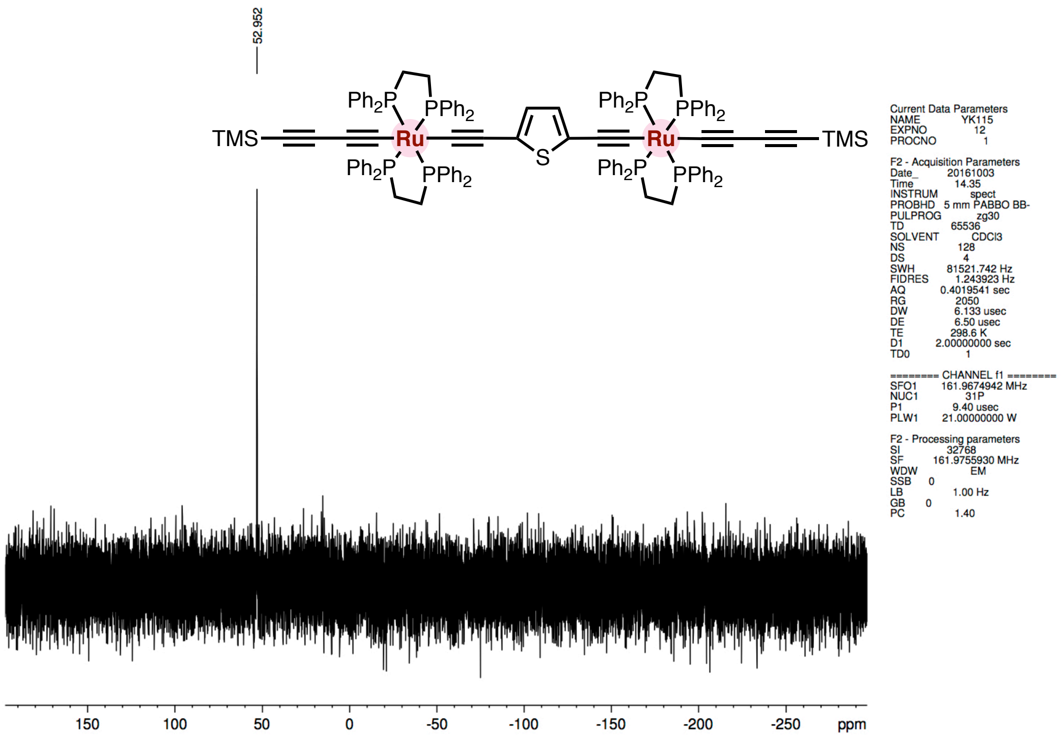

Figure S5b. ^{31}P NMR spectrum of $\mathbf{2}^{\mathbf{TMS}}$ (162MHz, $CDCl_3$, r.t.).

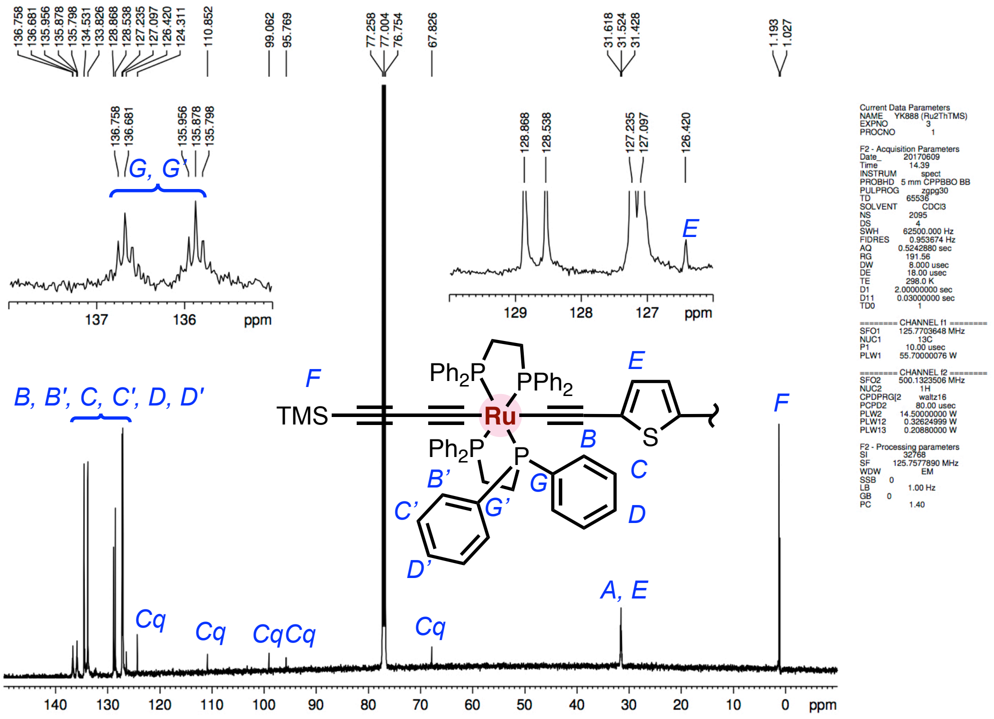

Figure S5c. ^{13}C NMR spectrum of $\mathbf{2^{TMS}}$ (126MHz, $CDCl_3$, r.t.).

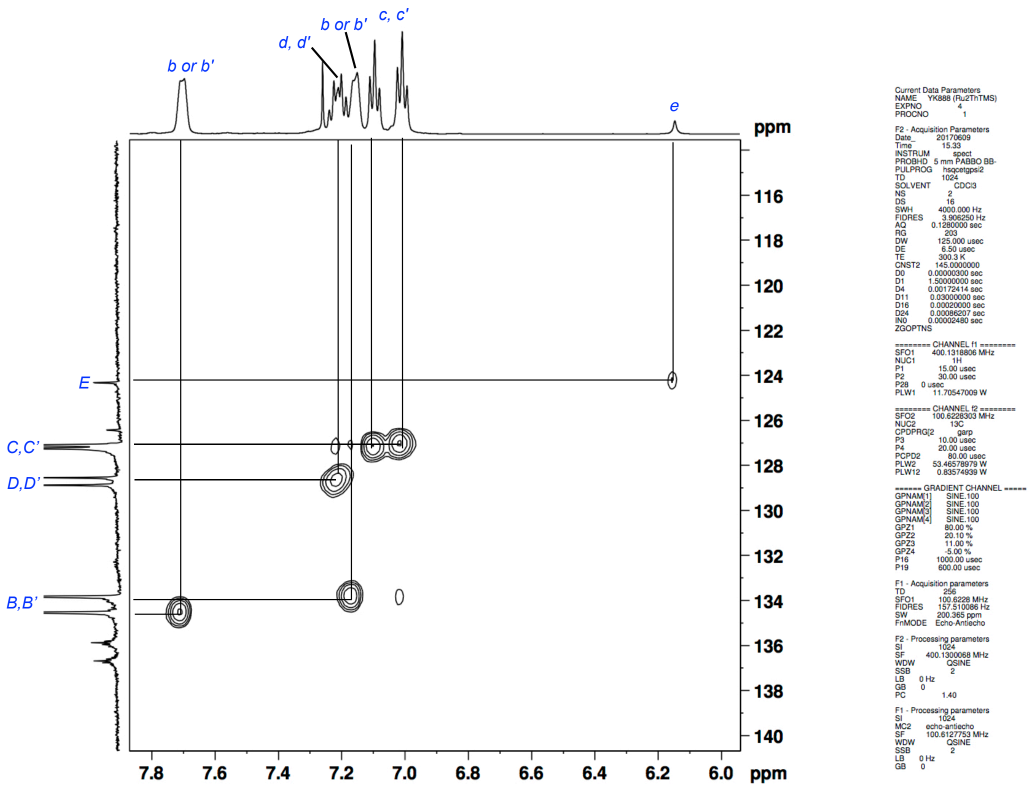

Figure S5d. A part of HSQC spectrum of $\mathbf{2^{TMS}}$ (400MHz, $CDCl_3$, r.t.).

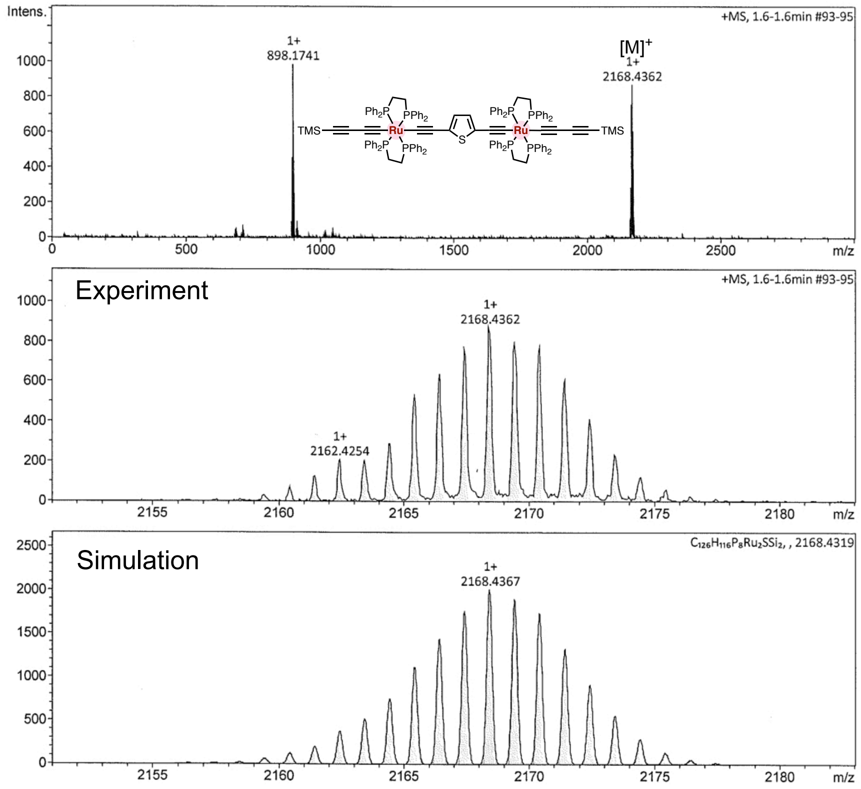

Figure S5e. HR-ESI-TOF-MS spectra of $\mathbf{2^{TMS}}$ (CH_2Cl_2).

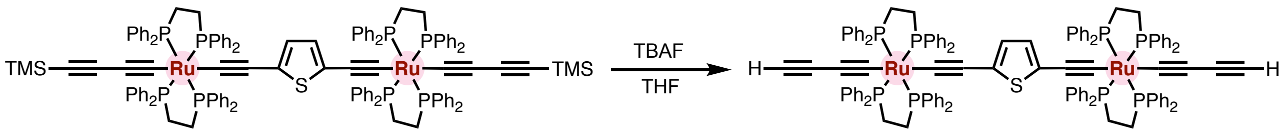

Synthesis of $\mathbf{2^{H}}$. To a THF solution (10 mL) of $\mathbf{2^{TMS}}$ (104 mg, 0.048 mmol, 1.0 eq) was added TBAF (138 μL, 1 M in THF, 0.138 mmol, 2.9 eq), and the resultant mixture was sttirred overnight at room temperature. The reaction mixture was evaporated *in vacuo*, and the residue was dissolved in CH_2Cl_2, and MeOH was added to the solution to give precipitates, which was washed with MeOH and dried under vacuum to give a yellow solid (51.0 mg, 0.025 mmol, 53%).

IR (KBr, cm^{-1}): ν(C≡C-H) 3303 (m), ν(C≡C) 2111 (s), 2053 (s), 1969 (m). ^{1}H NMR (400 MHz, C_6D_6, r.t.): δ 1.40 (s, 2H, C≡C-H), 2.51-2.83 (m, 16H, dppe-CH_2), 6.17 (s, 2H, Th), 6.96-7.35 (m, 64H, dppe-Ph), 7.70-7.73 (m, 16H, *o*-phenyl). ^{31}P NMR (162 MHz, C_6D_6, r.t.): δ 52.9. HR-ESI-TOF-MS (CH_2Cl_2, CsI): *m/z* Calcd. for $C_{120}H_{100}P_8Ru_2S$: 2024.3577, Found 2024.3571 $[M]^+$.

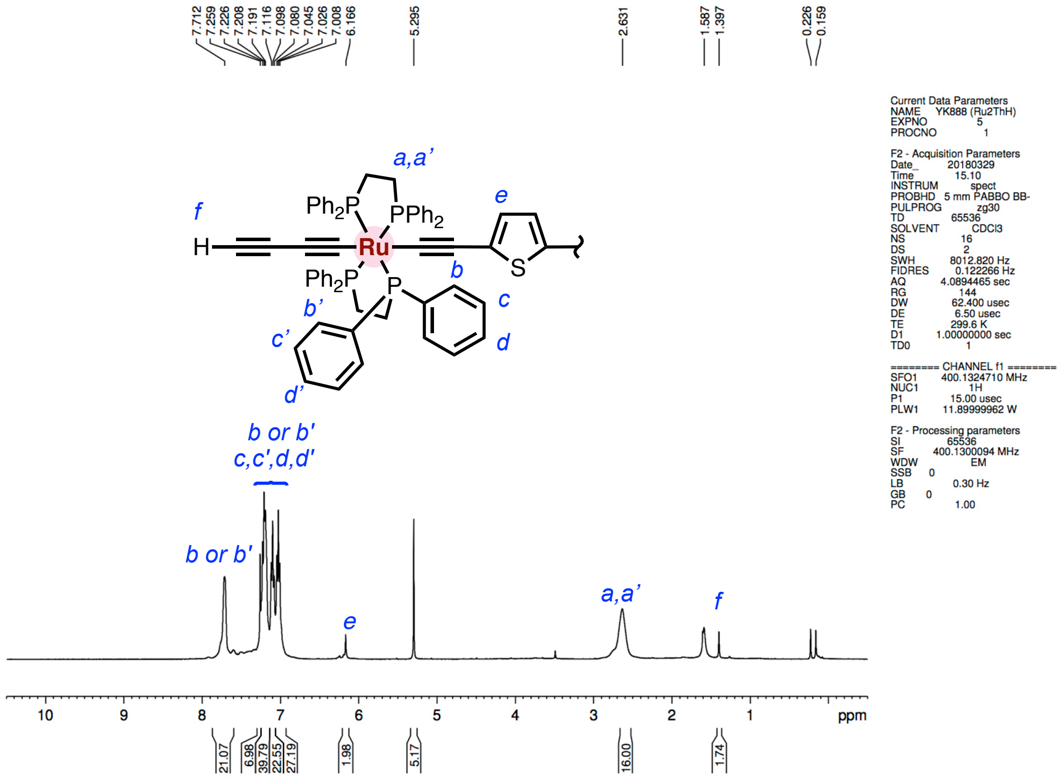

Figure S6a. ^{1}H NMR spectrum of $\mathbf{2^{H}}$ (400MHz, $CDCl_3$, r.t.).

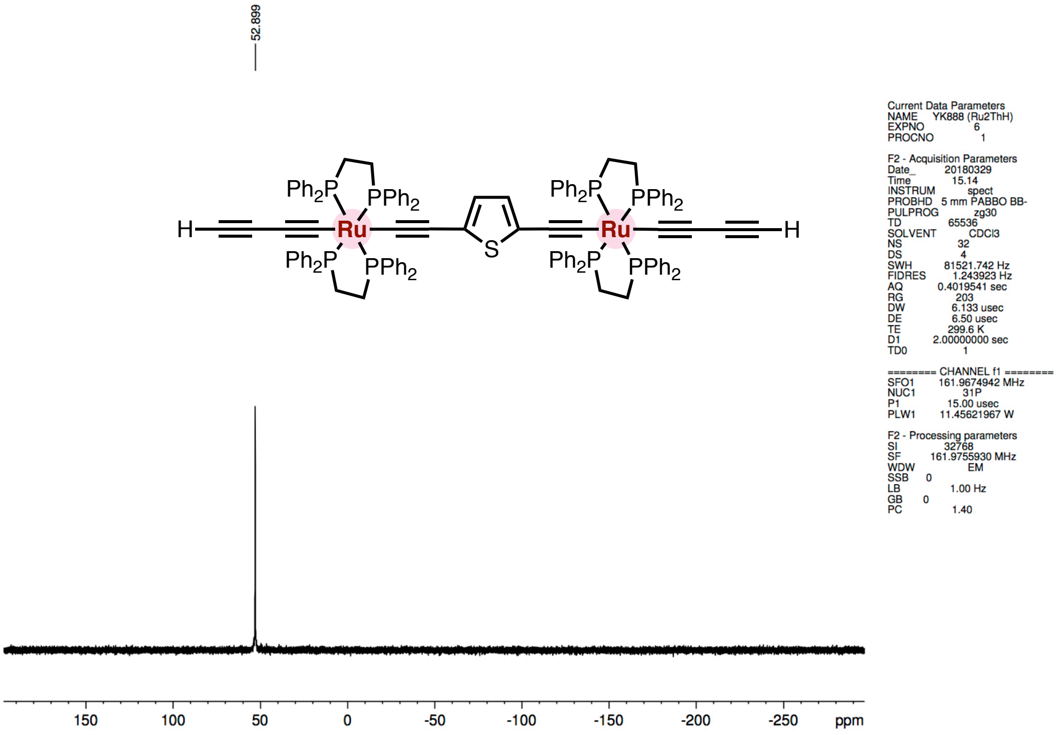

Figure S6b. ^{31}P NMR spectrum of $\mathbf{2^H}$ (162MHz, $CDCl_3$, r.t.).

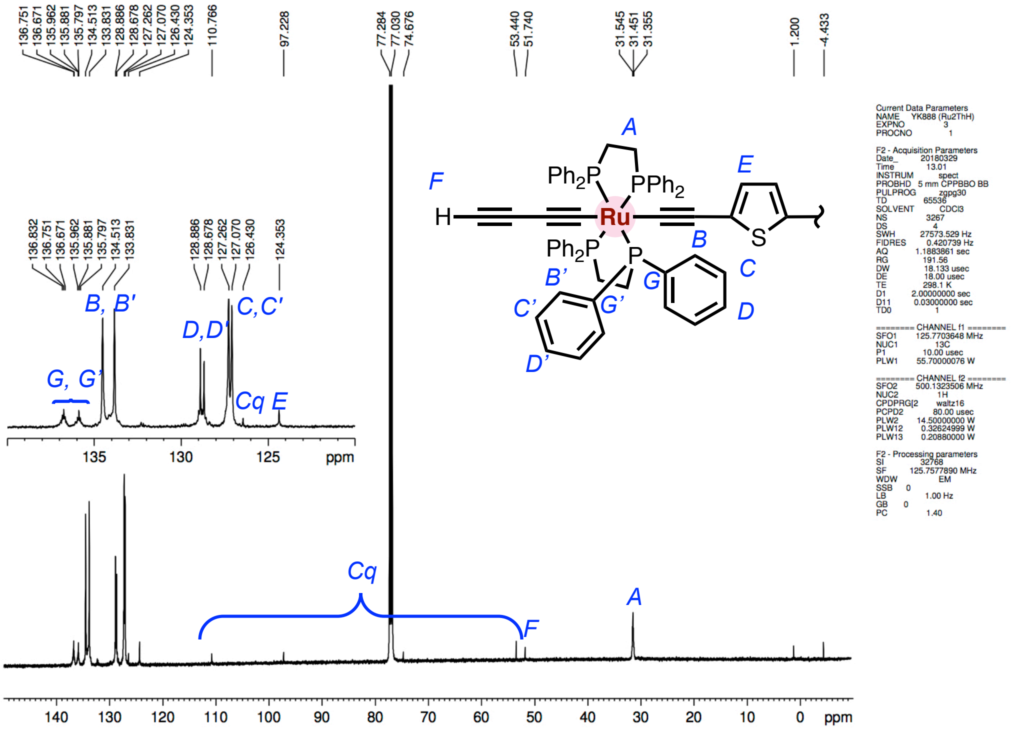

Figure S6c. ^{13}C NMR spectrum of $\mathbf{2^H}$ (126MHz, $CDCl_3$, r.t.).

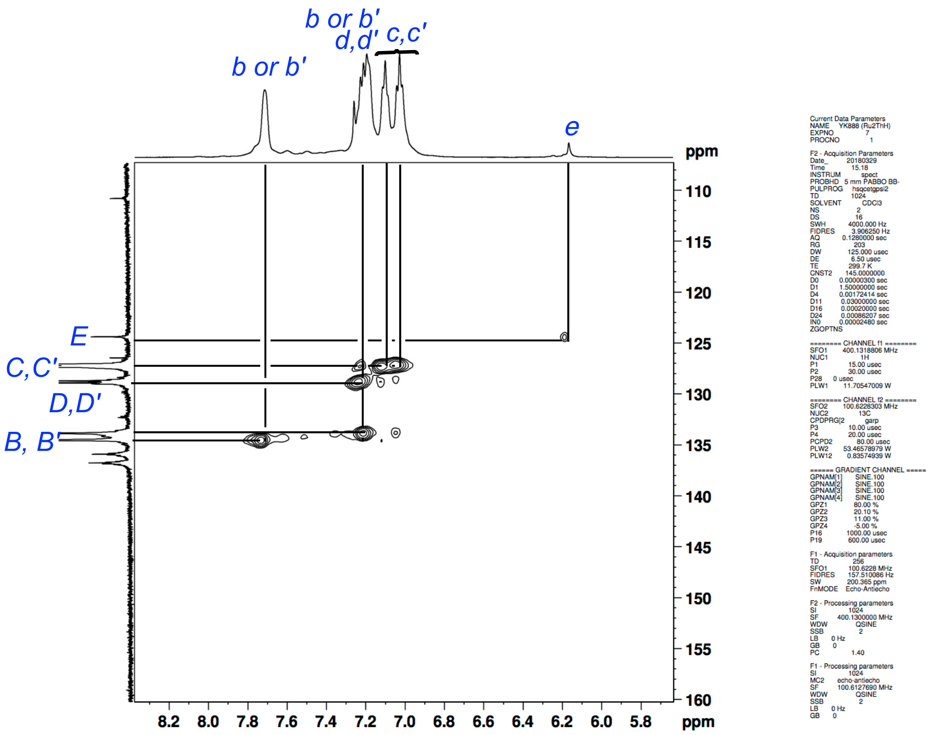

Figure S6d. A part of HSQC spectrum of **2^H** (400MHz, $CDCl_3$, r.t.).

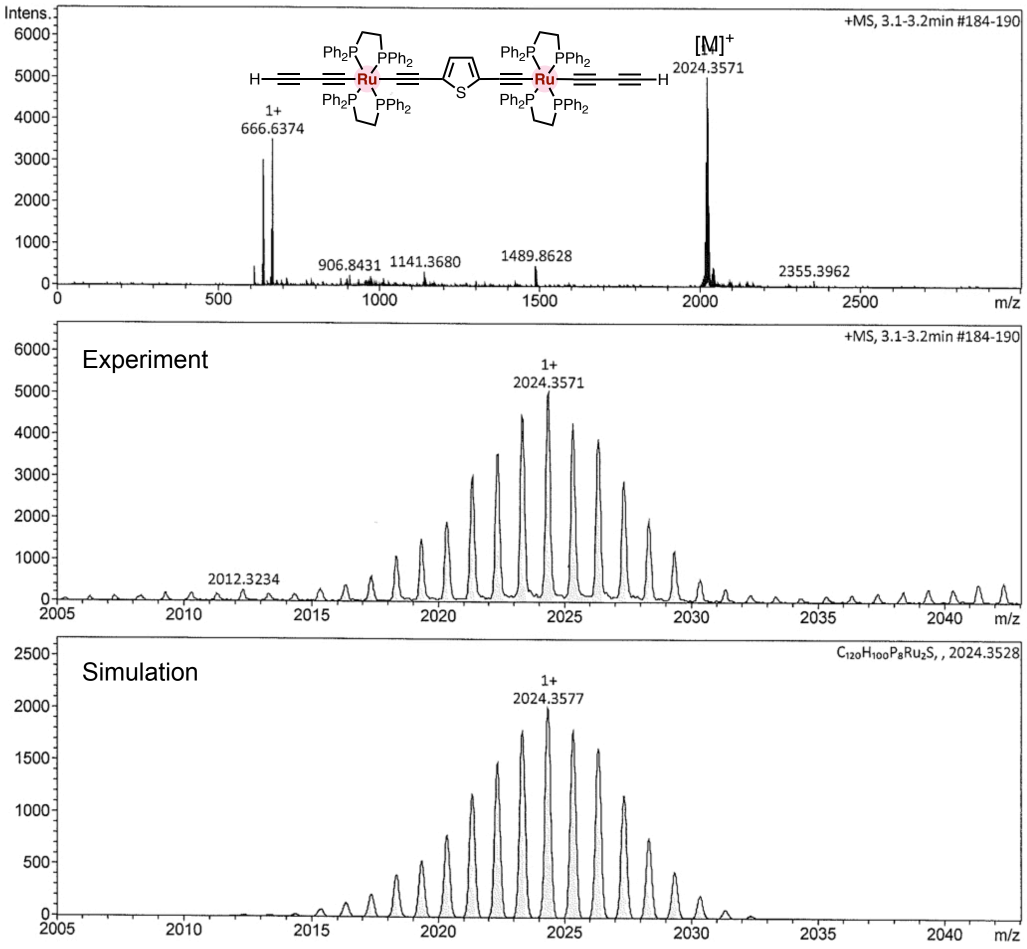

Figure S6e. HR-ESI-TOF-MS spectra of **2^H** (CH_2Cl_2).

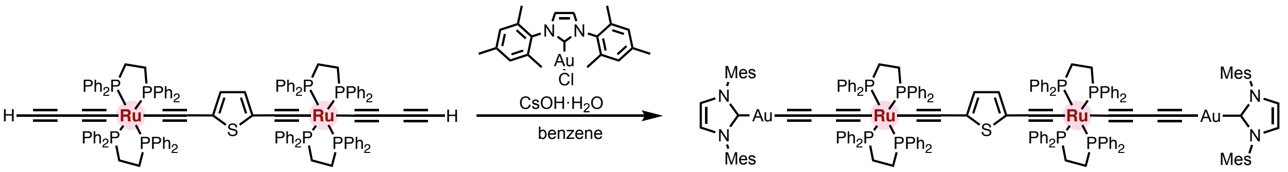

Synthesis of 2^{Au}. To a benzene solution (5 mL) of 2^{H} (104 mg, 0.048 mmol, 1.0 eq) and AuNHC(Mes)Cl (66.5 mg, 0.124 mmol, 2.3 eq) was added CsOH・H_2O (84.0 mg, 1.20 mmol, 22.0 eq) and the resultant mixture was stirred for 2 days at 70℃. The supernatant was transfered *via* cannula, evaporated *in vacuo*, and the residue was reprecipitated with CH_2Cl_2 / hexane. The filtrate was collected and evaporated *in vacuo* and washed with hexane to give a yellow solid (56.4 mg, 0.019 mmol, 34%).

IR (KBr, cm^{-1}): ν(C≡C) 2111 (s), 2053 (s), 1969 (m),ν(C≡C-H) 3303(m). ^{1}H NMR (500 MHz, $CDCl_3$, r.t.): δ 2,30 (s, 24H, Mes-CH_3), 2,47 (s, 12H, Mes-CH_3), 2.48–2.83 (m, 16H, dppe-CH_2), 6,07 (s, 2H, Th), 6.83 (br, 4H, Mes-CH), 7.00-7.60 (m, 80H, dppe-Ph and Mes-Ar). ^{31}P NMR (162 MHz, $CDCl_3$, r.t.): δ 52.5 (s, 8P, Ru(dppe)$_2$). ^{13}C NMR (126 MHz, $CDCl_3$, r.t.): δ 18.0, 21.3, 31.3 (*t*, *J* = 12.1 Hz), 95.7, 101.5, 105.9, 110.1, 121.9, 123.9, 126.4, 127.0, 127.1, 128.3, 128.4, 129.3, 134.1, 134.4, 135.1, 135.3, 136.6 (quint, *J* = 10.3 Hz), 137.2 (quint, *J* = 10.3 Hz), 139.3, 190.4. Quaternary carbon atoms of acetylene α carbon are not observed. HR–ESI-TOF-MS (CH_2Cl_2): *m/z* Calcd. for $C_{162}H_{146}Au_2N_4P_8Ru_2S$: 3024.6643, Found 3025.6641 $[M]^+$. Anal. Calcd. for $C_{162}H_{146}Au_2N_4P_8Ru_2S$: C, 64.33 ; H, 4.87; N, 1.85, Found : C, 63.39 ; H, 5.18; N, 2.03.

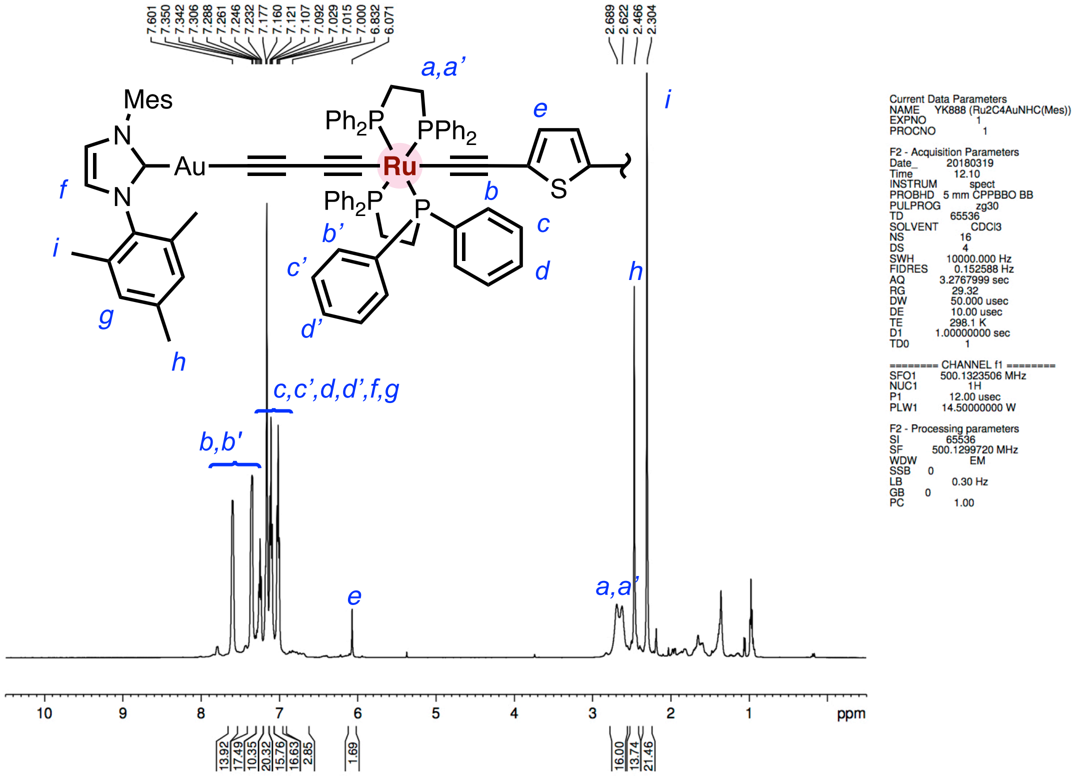

Figure S7a. ^{1}H NMR spectrum of 2^{Au} (500MHz, $CDCl_3$, r.t.).

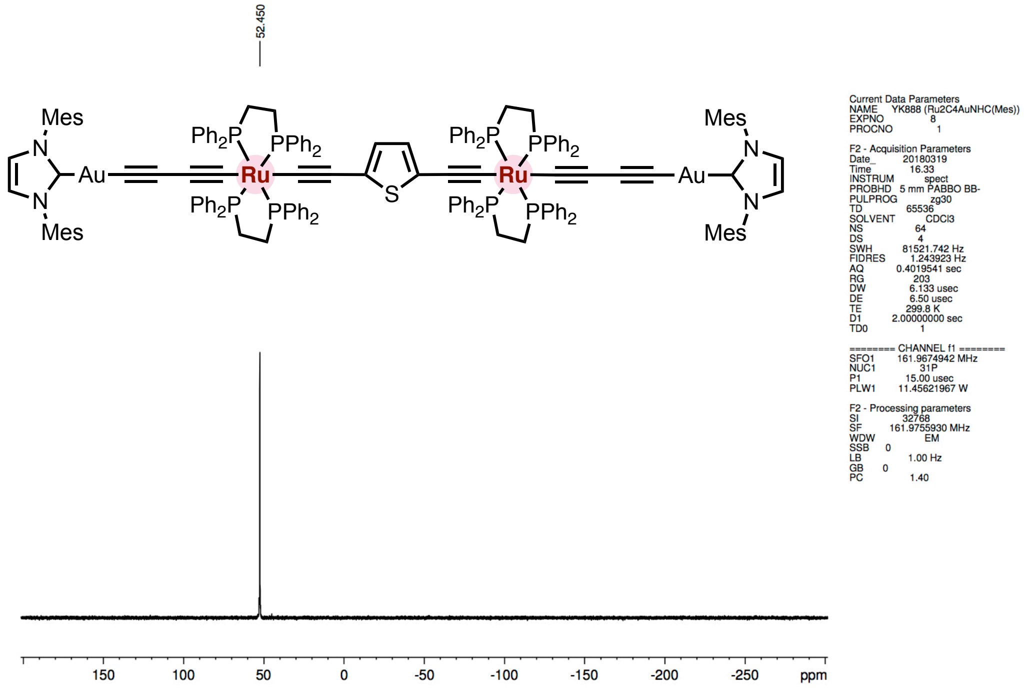

Figure S7b. ^{31}P NMR spectrum of $\mathbf{2^{Au}}$ (162 MHz, $CDCl_3$, r.t.).

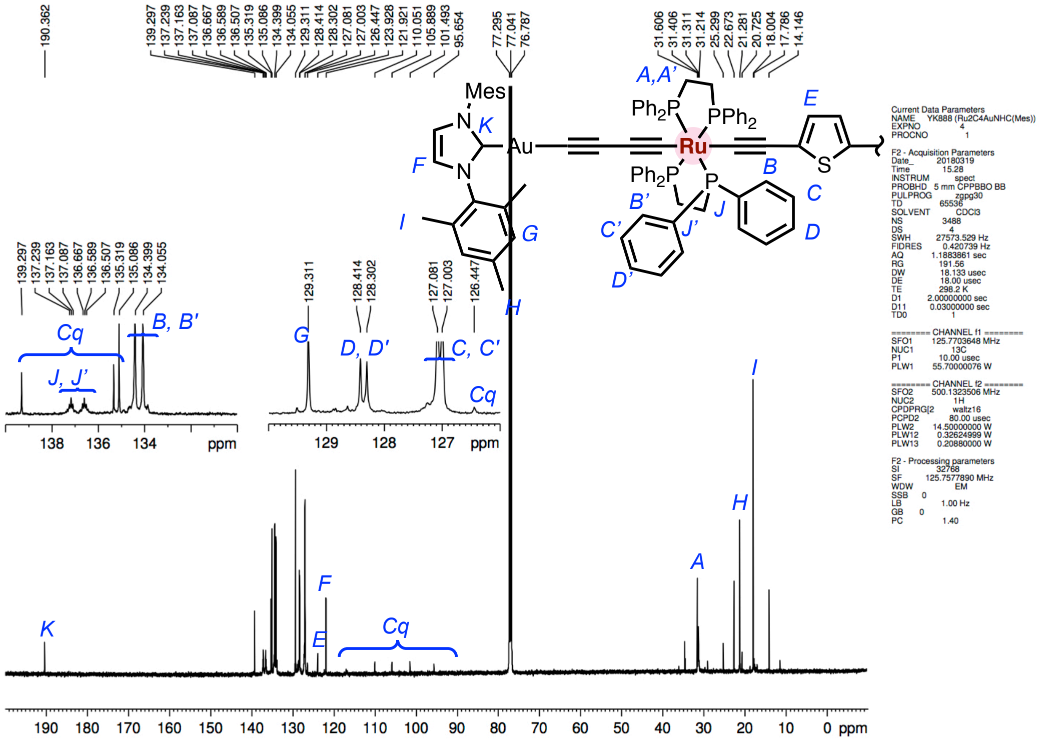

Figure S7c. ^{13}C NMR spectrum of $\mathbf{2^{Au}}$ (126MHz, $CDCl_3$, r.t.).

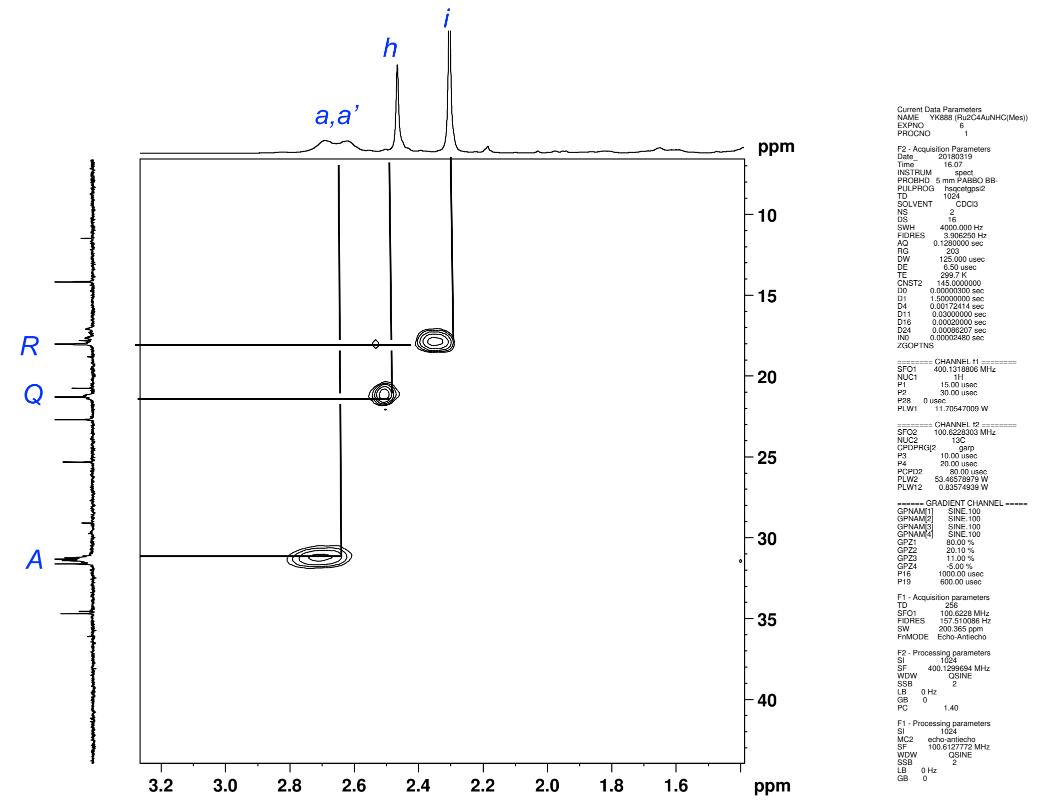

Figure S7d. A part of HSQC spectrum of $\mathbf{2}^{\mathbf{Au}}$ (400MHz, $CDCl_3$, r.t.).

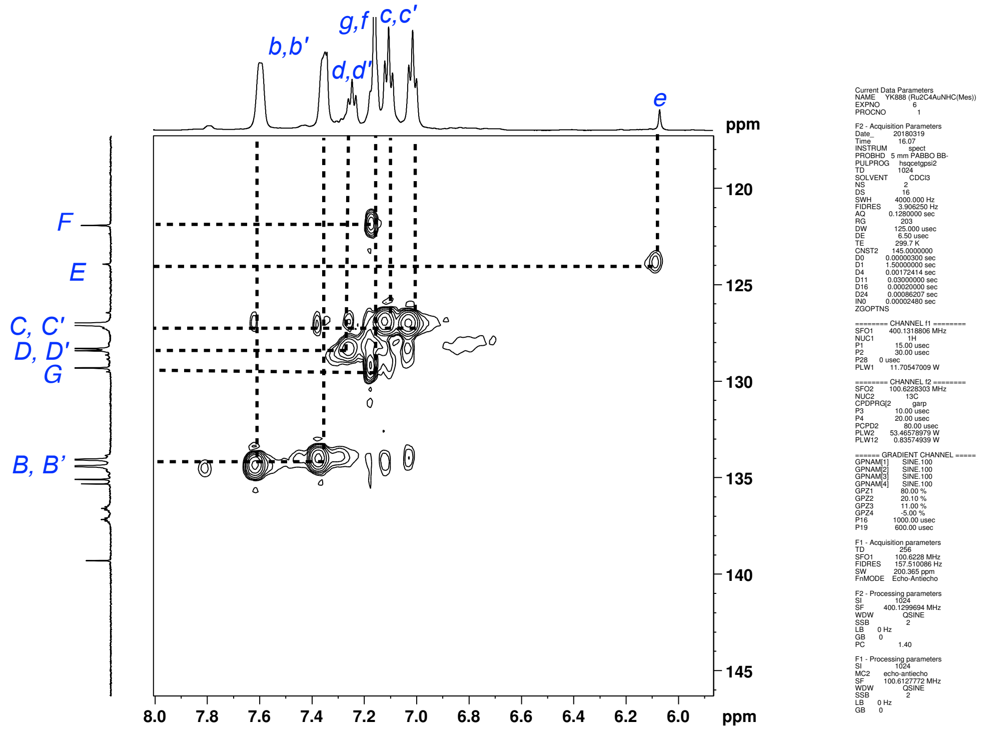

Figure S7e. A part of HSQC spectrum of $\mathbf{2}^{\mathbf{Au}}$ (400MHz, $CDCl_3$, r.t.).

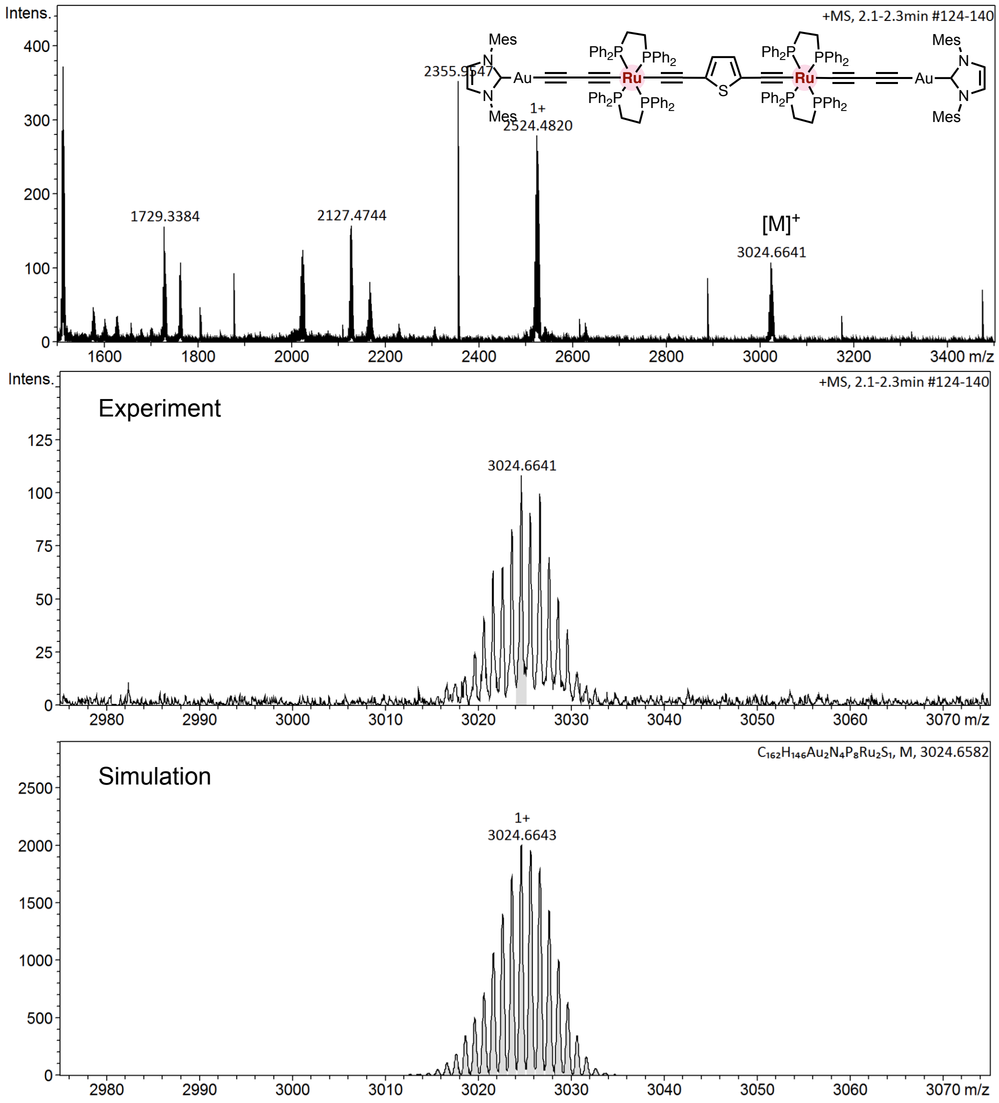

Figure S7f. HR-ESI-TOF-MS spectra of $\mathbf{2^{Au}}$ (CH_2Cl_2).

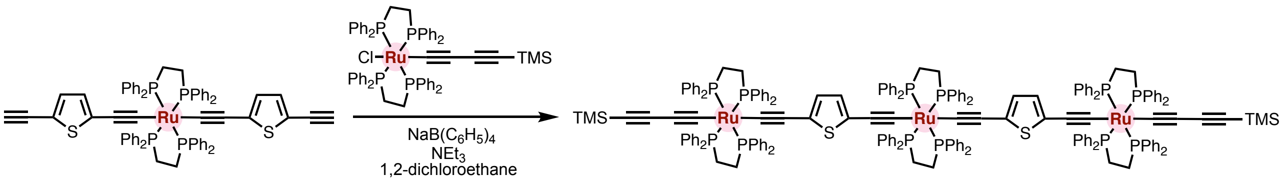

Synthesis of 3^{TMS}. To a mixture of Cl-Ru(dppe)$_2${(C≡C)$_2$TMS} (272 mg, 0.259 mmol, 2.0 eq) and $NaBPh_4$ (220.0 mg, 0.645 mmol, 5.0 eq) in 1,2-dichloroethane (10 mL) was added NEt_3 (1.0 mL) and (HC≡C-C_4SH_2-C≡C)$_2$Ru(dppe)$_2$ (150 mg, 0.129 mmol, 1.0 eq), and the resultant mixture was sttirred overnight at room temperature. The reaction mixture was filtered *via* cannula and washed with MeOH to give a yellow solid (377 mg, 0.118 mmol, 91%).

IR (KBr, cm^{-1}): ν(C≡C) 2166 (w), 2111 (m), 2052 (m), 1995(w). ^{1}H NMR (500 MHz, $CDCl_3$, r.t.): δ 0.245 (s, 18H, TMS), δ 2.50–2.82 (m, 24H, dppe-CH_2), 7.02-7.79 (m, 124H, dppe-Ph and Th). ^{31}P NMR (202 MHz, C_6D_6, r.t.): δ 56.6 (br). HR-ESI-TOF-MS (CH_2Cl_2, CsI): *m/z* Calcd. for $C_{186}H_{166}P_{12}Ru_3S_2Si_2$:1598.3009, Found 1598.2930 $[M]^{2+}$. Anal. Calcd. for $C_{186}H_{166}Au_2P_{12}Ru_3S_2Si_2$: C, 69.89 ; H, 5.23, Found : C, 68.59 ; H, 4.97.

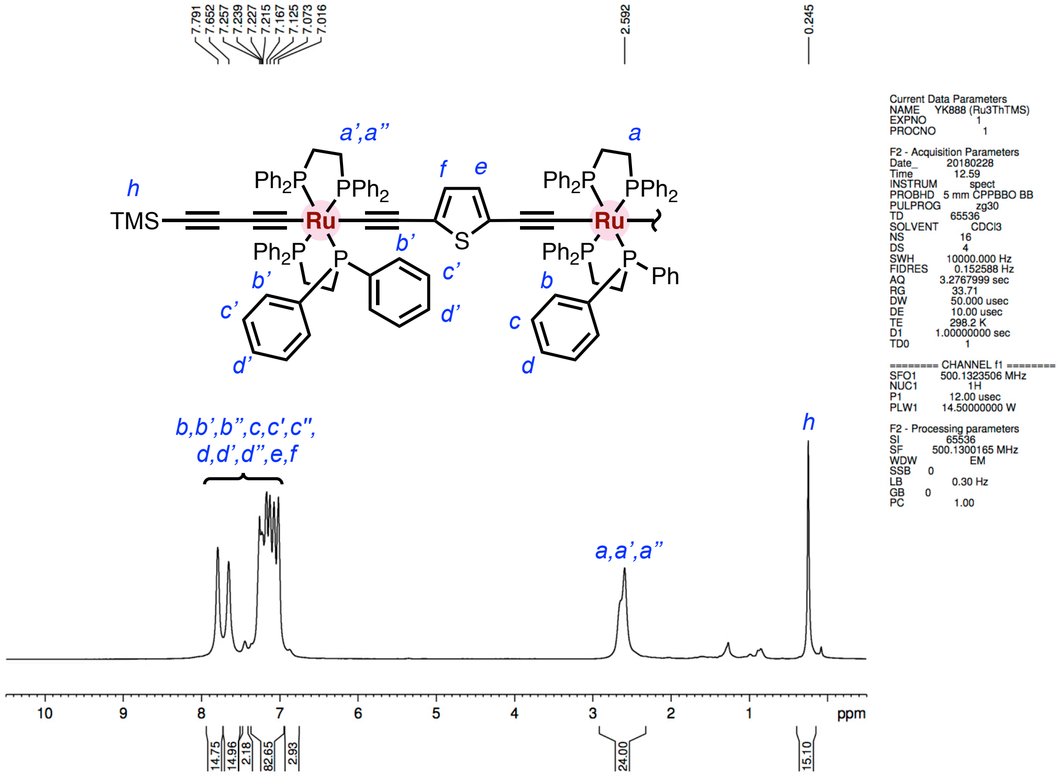

Figure S8a. ^{1}H NMR spectrum of $\mathbf{3^{TMS}}$ (400MHz, $CDCl_3$, r.t.).

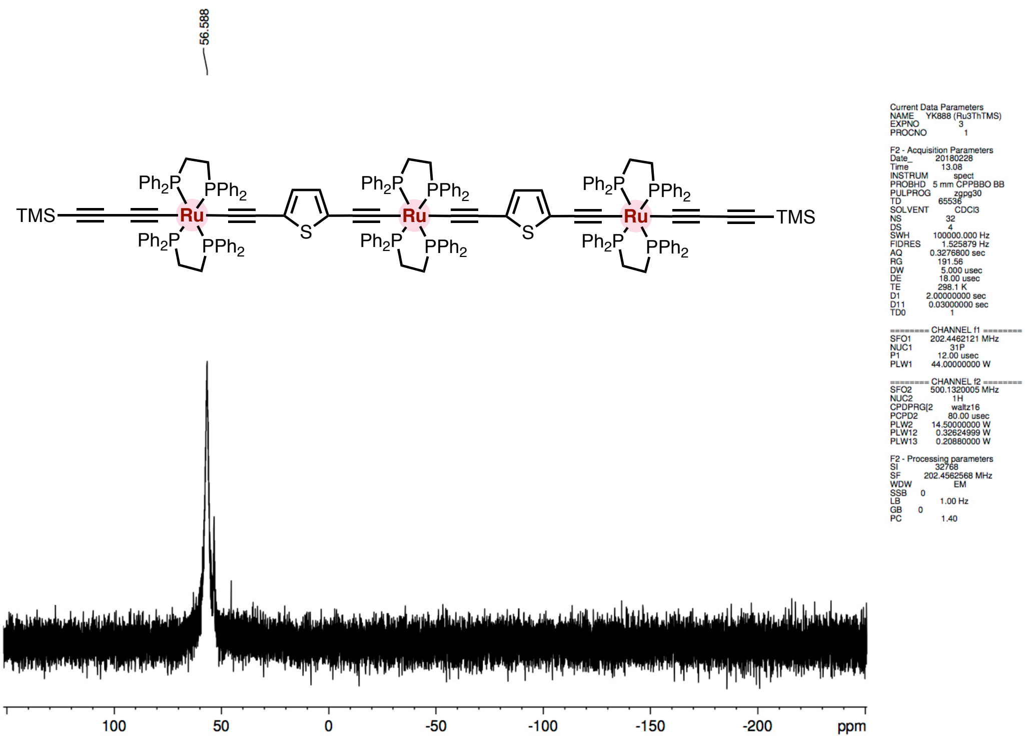

Figure S8b. ^{31}P NMR spectrum of **3^{TMS}** (162MHz, $CDCl_3$, r.t.).

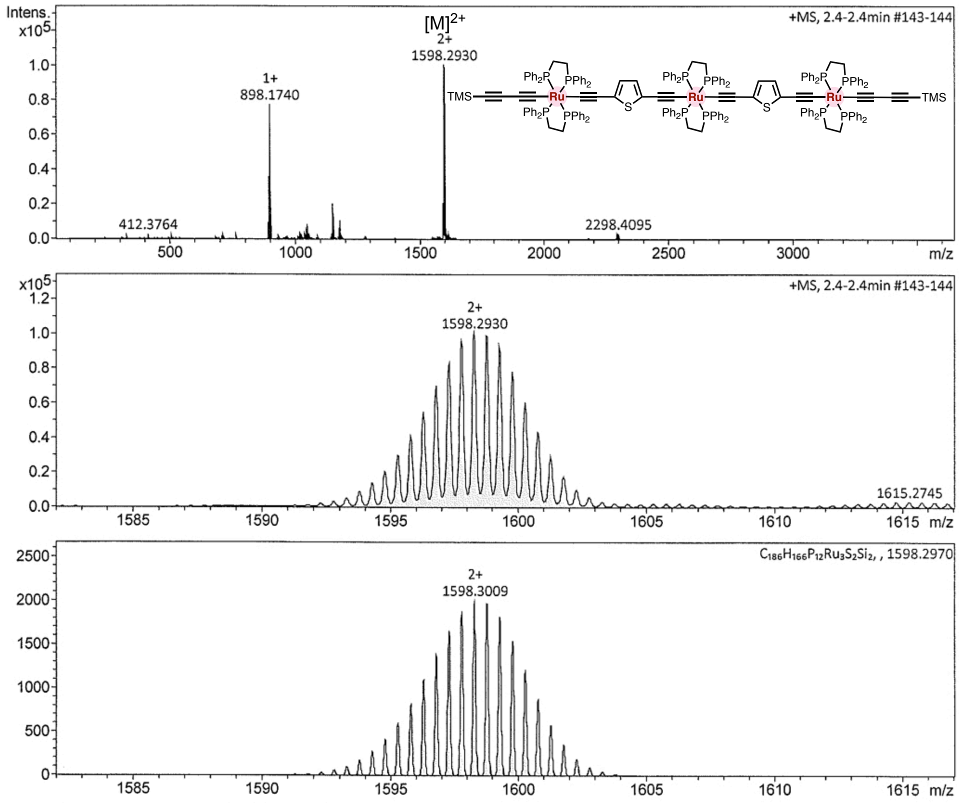

Figure S8c. HR-ESI-TOF-MS spectra of **3^{TMS}** (CH_2Cl_2).

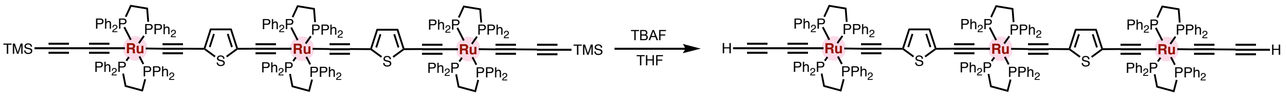

Synthesis of 3^{H}. To a THF solution (7 mL) of 3^{TMS} (98.5 mg, 0.030 mmol, 1.0 eq) was added TBAF (0.1 mL, 1M in THF, 0.1 mmol, 3.3 eq), and the resultant mixture was sttirred for 2 hours at room temperature. The reaction mixture was concentrated, and diethyl ether was added to the solution to give precipitates, which was washed with MeOH to give a yellowish green solid (66.0 mg, 0.022 mmol, 70%).

IR (KBr, cm^{-1}): ν(C≡C-H) 3304(m), ν(C≡C) 2111 (s), 2047 (s), 1970 (w). ^{1}H NMR (400 MHz, C_6D_6, r.t.): δ 1.39 (s, 2H, C≡C-H), 2.55–2.73 (m, 24H, dppe-CH_2), 6.05-6.20 (m, 4H, Th), 6.96-7.90 (m, 120H, Ph). ^{31}P NMR (162 MHz, C_6D_6, r.t.): δ 53.0, 53.4. HR-ESI-TOF-MS (CH_2Cl_2, CsI): *m/z* Calcd. for $C_{180}H_{150}P_{12}Ru_3S_2$: 3052.5234, Found 3052.5127 $[M]^{+}$.

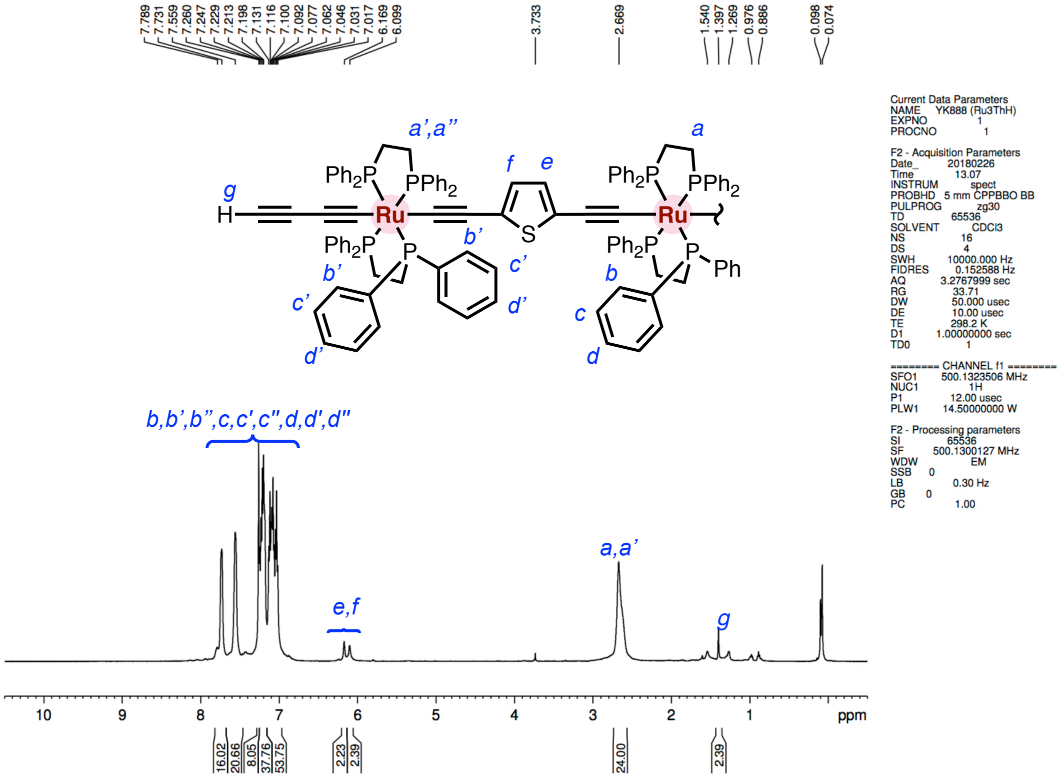

Figure S9a. ^{1}H NMR spectrum of 3^{H} (500MHz, $CDCl_3$, r.t.).

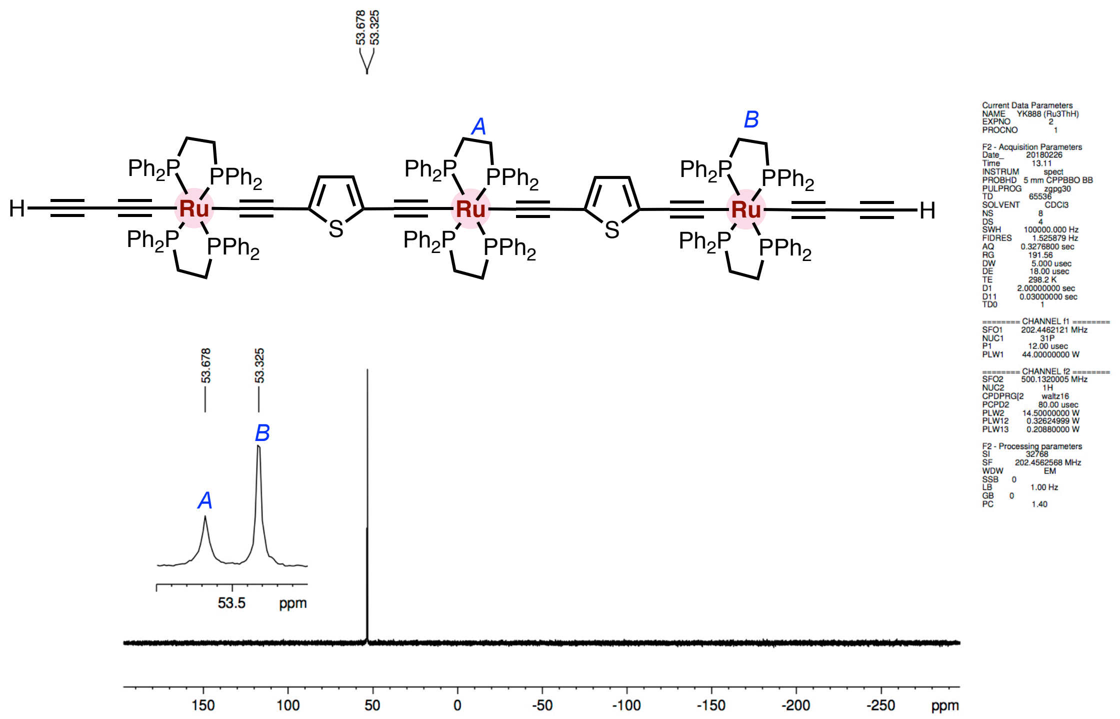

Figure S9b. ^{31}P NMR spectrum of $\mathbf{3^H}$ (202MHz, $CDCl_3$, r.t.).

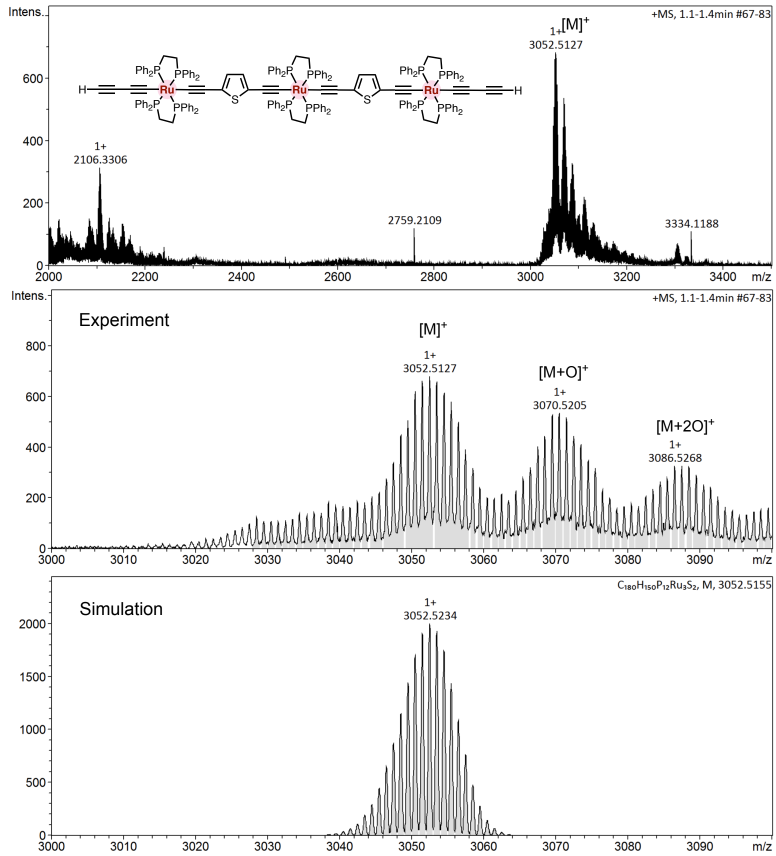

Figure S9c. HR-ESI-TOF-MS spectra of $\mathbf{3^H}$ (CH_2Cl_2).

Oxygen atom adducts were also observed under the MS condition.

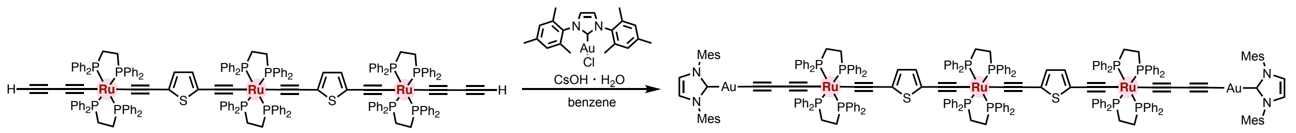

Synthesis of 3^{Au}. To a benzene solution (5 mL) of 3^{H} (51.5 mg, 0.017 mmol, 1.0 eq) and AuNHC(Mes)Cl (22.0 mg, 0.041 mmol, 2.4 eq) was added CsOH・H_2O (60.0 mg, 0.36 mmol, 21.0 eq) and the resultant mixture was sttirred for 2 days at 70℃. The reaction mixture was filtered through *via* cannula and was recrystallized in benzene / hexane and to give a yellow crystalline solid (32.5 mg, 0.008 mmol, 48%).

IR (KBr, cm^{-1}): ν(C≡C) 2115 (w), 2046 (br). ^{1}H NMR (400 MHz, C_6D_6, r.t.): δ 1,98 (s, 24H, CH_3), 2,15 (s, 12H, Mes-CH_3), 2.4-2.65 (m, 24H, dppe-CH_2), 5,97 (s, 4H, NHC-CH), 6.64 (s, 4H, Th), 6,73 (s, 8H, Mes-Ar), 6.89-7.20 (m, 72H, dppe-Ph), 7.47-7.53 (m, 16H, *o*-phenyl), 7.71-7.83 (m, 32H, *o*-phenyl). ^{31}P NMR (162 MHz, C_6D_6, r.t.): δ 52.9, 53.5, HR-ESI-TOF-MS (CH_2Cl_2): *m/z* Calcd. for $C_{222}H_{196}Au_2N_4P_{12}Ru_3S_2$: 4052.8301, Found 4052.8328 $[M]^{+}$. Anal. Calcd. for $C_{222}H_{196}Au_2N_4P_{12}Ru_3S_2$: C, 65.79 ; H, 4.87; N, 1.38, Found : C, 63.62 ; H, 4.89; N, 1.54.

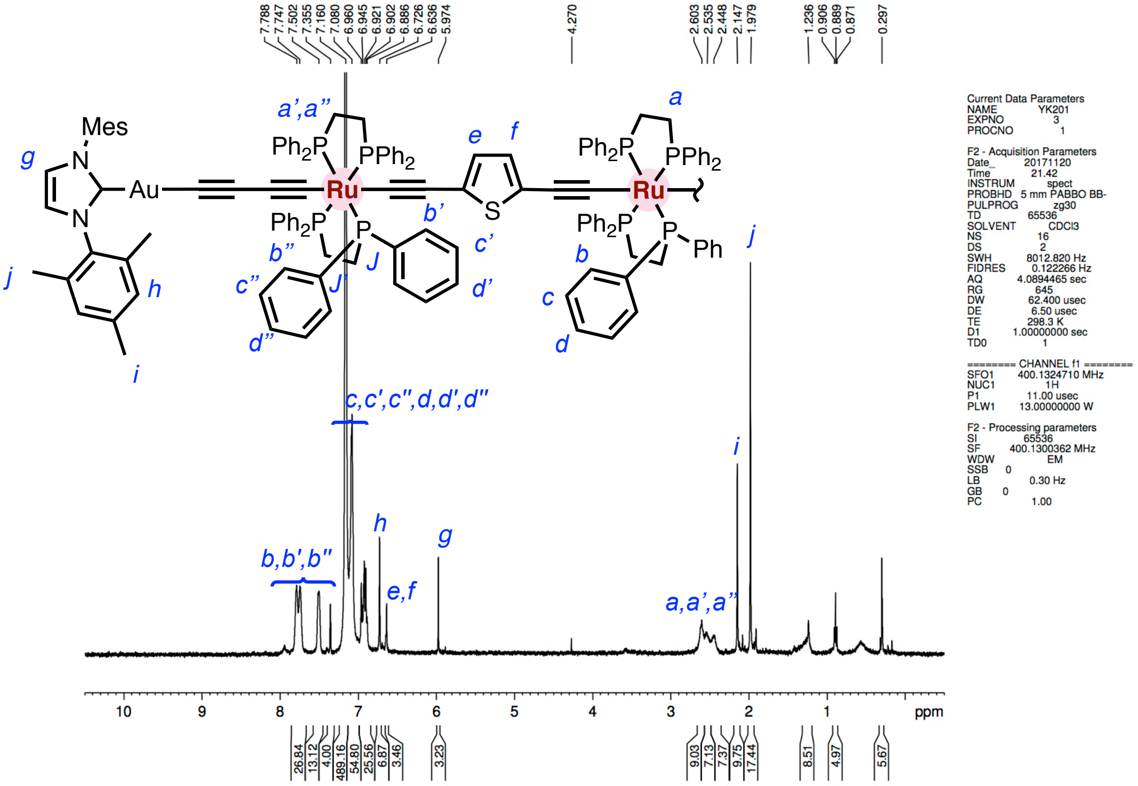

Figure S10a. ^{1}H NMR spectrum of 3^{Au} (400MHz, C_6D_6, r.t.).

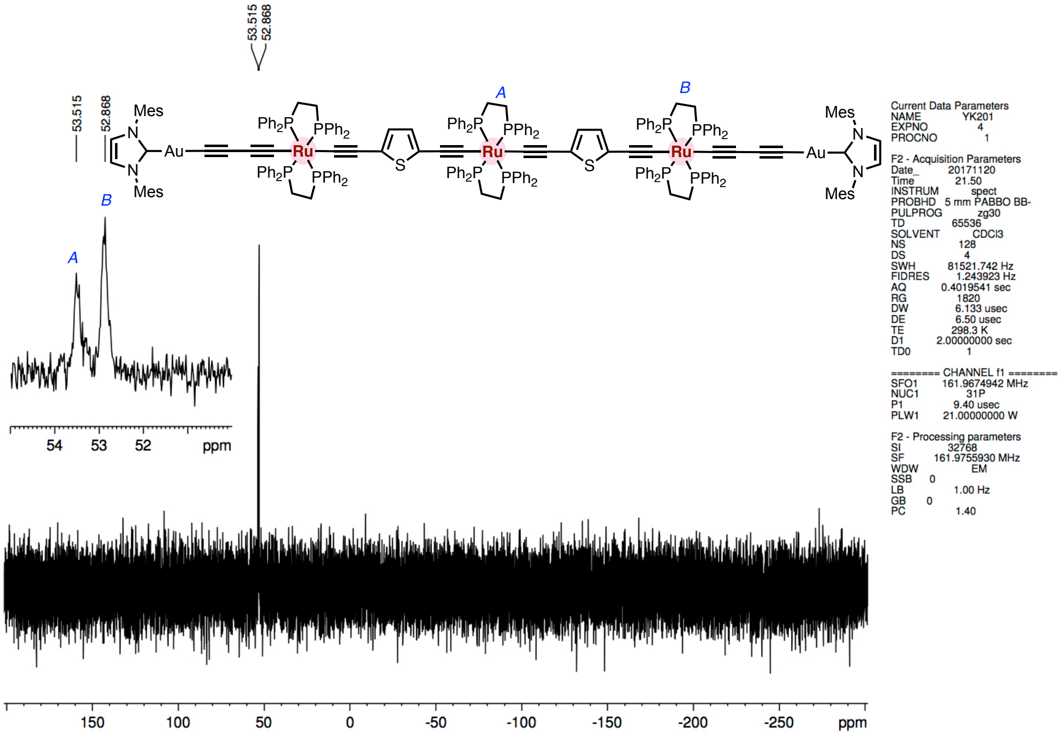

Figure S10b. ^{31}P NMR spectrum of $\mathbf{3^{Au}}$ (162MHz, C_6D_6, r.t.).

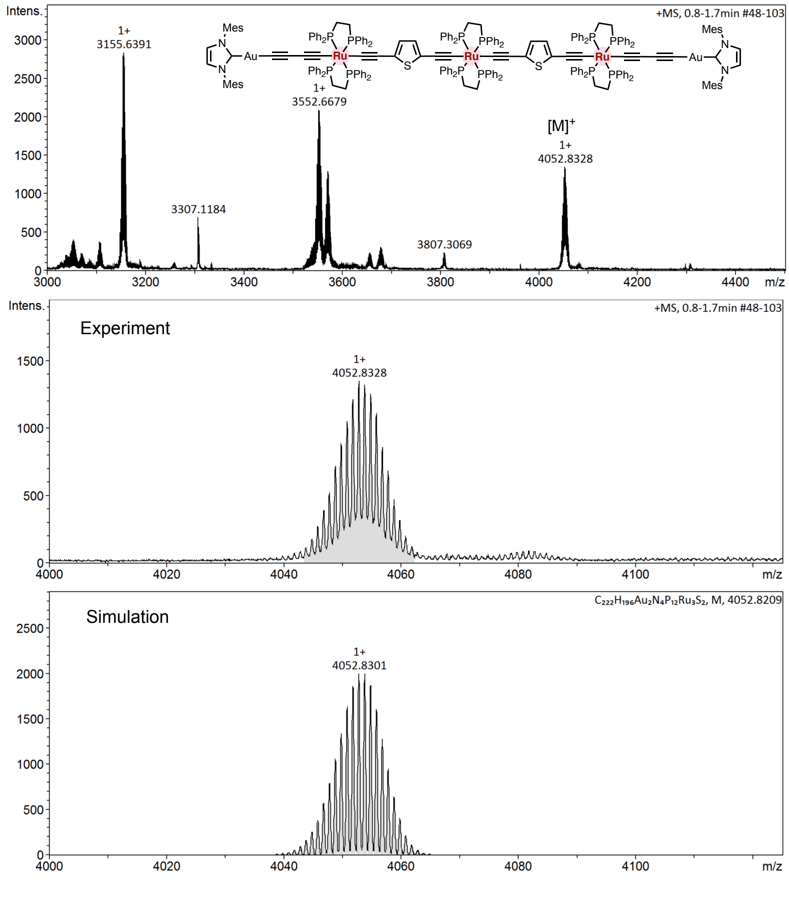

Figure S10c. HR-ESI-TOF-MS spectra of $\mathbf{3^{Au}}$ **(**CH_2Cl_2).

III. X-ray crystallographic data

Table S1. Crystal data and structure refinement for $\mathbf{2^{Py}}$, $\mathbf{2^{TMS}}$, and $\mathbf{3^{TMS}}$.

complex	$\mathbf{2^{Py}}$	$\mathbf{2^{TMS}}$	$\mathbf{3^{TMS}}$
Empirical formula	$C_{144}H_{124}N_2P_8Ru_2S$	$C_{126}H_{116}P_8Ru_2SSi_2$	$C_{198}H_{178}P_{12}Ru_3S_2Si_2$
Formula weight	2364.42	2168.33	3352.56
Temperature/K	90(2)	90(2)	93(2)
Crystal system	orthorhombic	triclinic	triclinic
Space group	$Pna2_1$	P-1	P-1
a/Å	25.3115(7)	10.1070(12)	12.3659(3)
b/Å	44.5483(14)	15.3835(18)	17.9279(5)
c/Å	10.2350(2)	37.020(4)	20.5933(5)
α/°	90	83.510(2)	108.974(2)
β/°	90	89.395(2)	100.290(2)
γ/°	90	86.371(2)	100.600(2)
Volume/Å^3	11540.8(5)	5707.5(11)	4101.7(2)
Z	4	2	1
ρ_{calc}g/cm^3	1.361	1.2617	1.357
μ/mm^{-1}	3.757	0.464	4.117
F(000)	4904.0	2248.0	1738.0
Crystal size/mm^3	0.15 × 0.06 × 0.05	0.87 × 0.09 × 0.06	0.16 × 0.07 × 0.04
Radiation	CuKα (λ = 1.54184)	Mo Kα (λ = 0.71073)	Cu Kα (λ = 1.54184)
2Θ range for data collection/°	6.902 to 149.748	2.78 to 50.06	4.694 to 149.542
Index ranges	-31 ≤ h ≤ 31, -54 ≤ k ≤ 55, -12 ≤ l ≤ 4	-12 ≤ h ≤ 11, -12 ≤ k ≤ 18, -44 ≤ l ≤ 43	-15 ≤ h ≤ 14, -22 ≤ k ≤ 22, -25 ≤ l ≤ 25
Reflections collected	36780	27399	56464
Independent reflections	23723 [R_{int} = 0.0469]	20140 [R_{int} = 0.0236]	16862 [R_{int} = 0.0740]
Data/restraints/parameters	23723/2831/1588	20140/1284/1463	16862/42/1001
Goodness-of-fit on F^2	1.038	1.033	1.077
Final R indexes [I>=2σ (I)]	R_1 = 0.0518, wR_2 = 0.1377	R_1 = 0.0664, wR_2 = 0.1577	R_1 = 0.0890, wR_2 = 0.2561
Final R indexes [all data]	R_1 = 0.0716, wR_2 = 0.1520	R_1 = 0.0904, wR_2 = 0.1684	R_1 = 0.1114, wR_2 = 0.2693
Largest diff. peak/hole / e Å^{-3}	1.91/-0.72	1.61/-1.71	1.91/-1.06

IV. STM-BJ study

The conductance measurements were performed using the scanning tunneling microscope (STM) break junction technique with electrochemical STM (Pico-SPM, Molecular Imaging Co.) and a PicoScan 3000 PicoSPM II CONTROLLER (Molecular Imaging Co.). Cells, beakers, ceramic tweezers, and Schlenk tubes used for STM-BJ measurements were cleaned by soaking with mixed acid (an equivolume mixture of sulfuric acid and nitric acid) prior to use. The STM-tips were made from an Au wire (0.30 mm diameter, ca. 1.3 cm long, and 99,99% purity, obtained from The Nilaco Corporation) coated with wax (Apiezon Wax W obtained from The Nilaco Corporation). The substrate of Au(111) was formed on surface of gold beads, which were prepared as follows. Au wires (0.90 mm diameter, ca. 5.0 cm long, and 99,99% purity, obtained from The Nilaco Corporation) were boiled in a concentrated HCl solution for more than 10 min, and then rinsed with ultrapure water. Then the Au wires were flame annealed until melted into a sphere to form Au beads. The Au beads were, then, gently remelted until they showed single-crystalline Au(111) surface. Solutions of molecular wires in tetraglyme (~1.0 mM) were used for the measurements. Conductance was measured during the breaking process under an applied bias of 100 mV.

Conductance histogram

Each break-junction measurement was repeated 100 times (1 set) and each set of the measurement was repeated 20 times (in total 2000 break-junction measurements for an individual experiment). The 20 sets of the measurements were repeated at least 3 times with different gold tips and substrates, and were acquired on different days. 1D linear histogram were made by 1 set of the measurements (100 traces), and 1D log and 2D histograms were constructed from successive 20 set of data. No preference or data selection was performed in making the histograms.

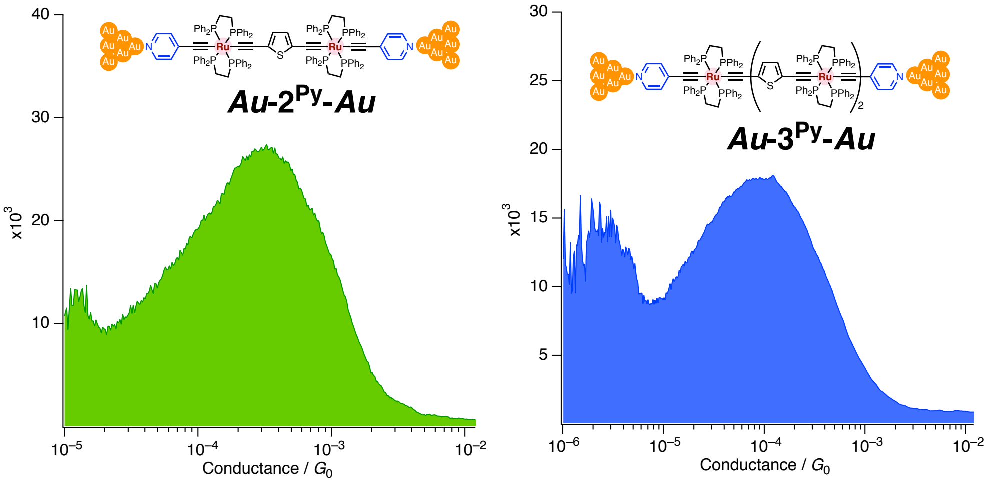

Figure S11. 1D log histograms of ***Au-n***$^{\mathbf{Py}}$***-Au*** (n = 2 and 3) constructed from 2000 traces without any data selection.

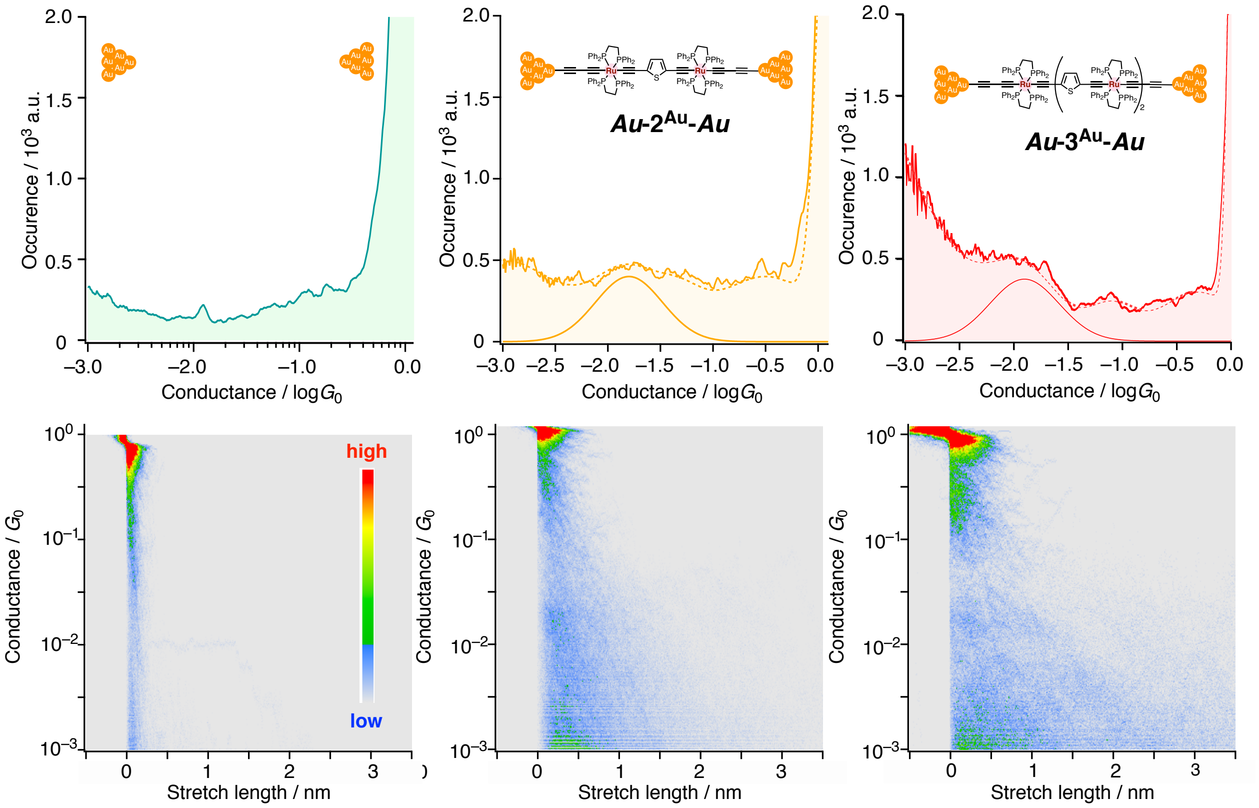

Figure S12. 1D (top) and 2D log histograms (bottom) of a tetraglyme solution (a blank experiment) and ***Au-n***$^{\mathbf{Au}}$***-Au*** (n = 2 and 3) complexes in tetraglyme. The histograms were constructed from 2000 traces without any data selection.

V. Discussion on metal-metal interactions in solution

Cyclic voltammograms of **2**^{R} and **3**^{R} (R = Py and TMS) show successive reversible redox waves, indicating strong metal-metal interactions through the diethynylthiophene bridging ligands (Figure S13). Then we performed spectroelectrochemical measurements. Because the results obtained for **2**^{R} and **3**^{R} (R = Py and TMS) were virtually the same (Figure S14-17), here, we focus on the TMS derivatives **2**^{TMS} and **3**^{TMS}. Upon applied bias voltages on a CH_2Cl_2 solution containing 1 mM of **2**^{TMS} and 0.1 M of $[NBu_4][PF_6]$ as electrolytes, intense NIR absorption bands appeared around 1000-2000 nm, which can be ascribed to the monocationic species [**2**^{TMS}]$^{+}$ (Figure S16). Further oxidation led to spectral changes, and the NIR bands of [**2**^{TMS}]$^{+}$ disappeared due to generation of dicationic [**2**^{TMS}]$^{2+}$ species. Similarly, we performed spectroelectrochemical measurements for the trinuclear analogue **3**^{TMS}. In this case, spectral changes due to generation of mono- ([**3**^{TMS}]$^{+}$), di- ([**3**^{TMS}]$^{2+}$) and tri-cationic species ([**3**^{TMS}]$^{3+}$) were observed (Figure S17). These NIR bands appeared to be a sum of overlapped multiple bands and their origin are due to presence of rotamers.[S12] Therefore, deconvolution analysis was performed for the NIR bands of [**2**^{TMS}]$^{+}$ and [**3**^{TMS}]$^{+}$, and three gaussian fitting curves were obtained (Band A-C).

To get some insight into the NIR bands origin, we performed DFT and TD-DFT study for the monocationic species (Figure S18,20-22). Monocationic species [**2**^{H}]$^{+}$ and [**3**^{H}]$^{+}$ are optimized with the BLYP35/DefSVP, CPCM(CH_2Cl_2) levels of theory, which is known to reproduce NIR bands of mixed-valence species.[S13] We calculated one of the most highly symmetrical conformers for each molecule. The spin density of [**2**^{H}]$^{+}$ is distributed over the Ru-CC-C_4H_2S-CC-Ru moiety. A large contribution on the thiophene part suggests the oxidation also occurs in the bridging ligand. Judging from the fact that the metal centers' spin density is nearly equal (Ru: 0.12-0.13), [**2**^{H}]$^{+}$ is classified as a Robin-day class III or a borderline of class II and class III. TD-DFT study suggests the lowest energy band of [**2**^{H}]$^{+}$ is derived from the transition from β-HOSO to β -LUSO, which can be ascribed to the π-π* transition of the delocalized orbital containing ruthenium centers. This character is typical for a fully delocalized class III compound with a significant bridging ligand oxidation state. Because of the large oscillator strength and the energy, band A in Figure S16 can be attributed to this rotamer.

Similarly, the trinuclear analogue [**3**^{H}]$^{+}$ was calculated. Spin density is localized on the central ruthenium centers together with the -CC-C_4H_2S-CC- linkers. On the other hand, there is little contribution to the terminal ruthenium centers, suggesting the class II system. A computed NIR band with large oscillator strength was assignable to band B in Figure S17. This NIR transition band is ascribed to the transition from β-HOSO to β-LUSO, which involves charge transfer character from terminal ruthenium centers to the central ruthenium center and π-π* character of the CC-C_4H_2S-CC-linkers. Thus, according to the theoretical study, there is no evidence for the intramolecular interaction between terminal ruthenium centers, whereas strong electronic interactions between adjacent ruthenium centers are supported.

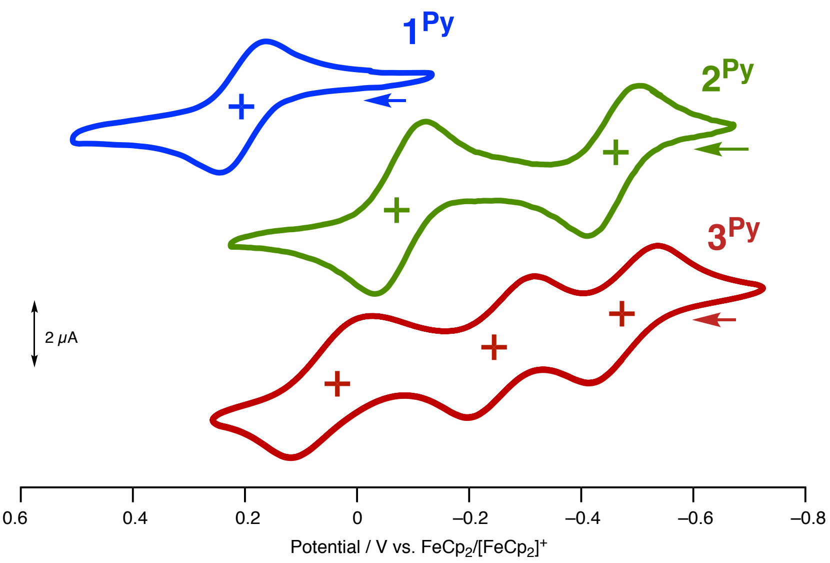

Figure S13. Cyclicvoltammograms of $\mathbf{1^{Py}}$-$\mathbf{3^{Py}}$ (0.1 mM in CH_2Cl_2, 0.1M [*n*-Bu_4N][PF_6]).)

Table S2. Electrochemical data for $\mathbf{1^R}$-$\mathbf{3^R}$
(R = Py and TMS, 0.1 mM in CH_2Cl_2, 0.1M [*n*-Bu_4N][PF_6]).

complex	E_{onset} [mV]	$E_{1/2}^1$ [mV]	$E_{1/2}^2$ [mV]	$E_{1/2}^3$ [mV]	ΔE_1 [mV]	ΔE_2 [mV]	Kc_1	Kc_2	HOMO[a] [eV]
$\mathbf{1^{Py}}$	130	190							–5.23
$\mathbf{2^{Py}}$	–440	–370	20		390		3.1×10^6		–4.66
$\mathbf{3^{Py}}$	–530	–460	–230	70	230	300	8.1×10^3	1.1×10^5	–4.57
$\mathbf{1^{TMS}}$	80	150							–5.18
$\mathbf{2^{TMS}}$	–430	–360	10		370		1.8×10^6		–4.67
$\mathbf{3^{TMS}}$	–510	–440	–200	100	240	300	1.2×10^4	1.2×10^5	–4.59

[a] $E_{HOMO} = -(5.1 + E_{onset}\,(V))$ / eV

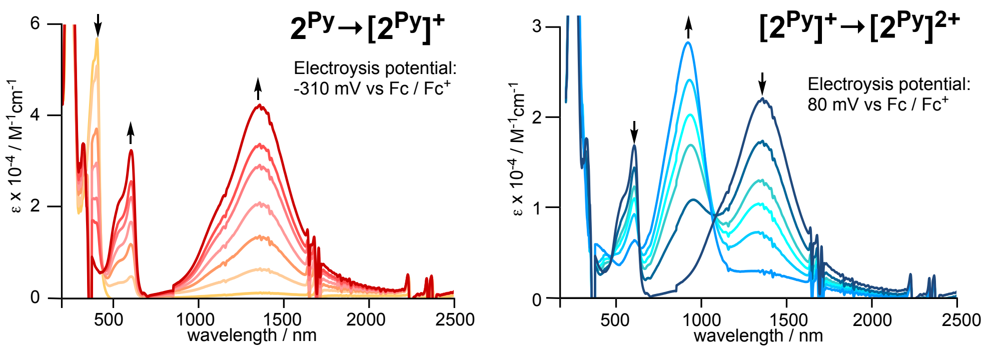

Figure S14. Absorption spectra of $[\mathbf{2^{Py}}]^+$ and $[\mathbf{2^{Py}}]^{2+}$ observed during the electrochemical oxidation (~0.1 mM in CH_2Cl_2, 0.1M [*n*-Bu_4N][PF_6]).

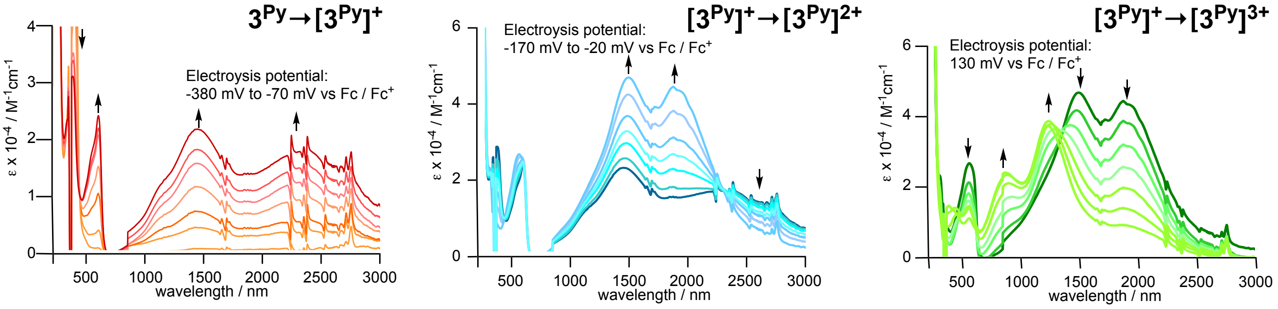

Figure S15. Absorption spectra of [$\mathbf{3^{Py}}$]$^+$, [$\mathbf{3^{Py}}$]$^{2+}$ and [$\mathbf{3^{Py}}$]$^{3+}$ observed during the electrochemical oxidation (~0.1 mM in CH_2Cl_2 , 0.1M [n-Bu_4N][PF_6]).

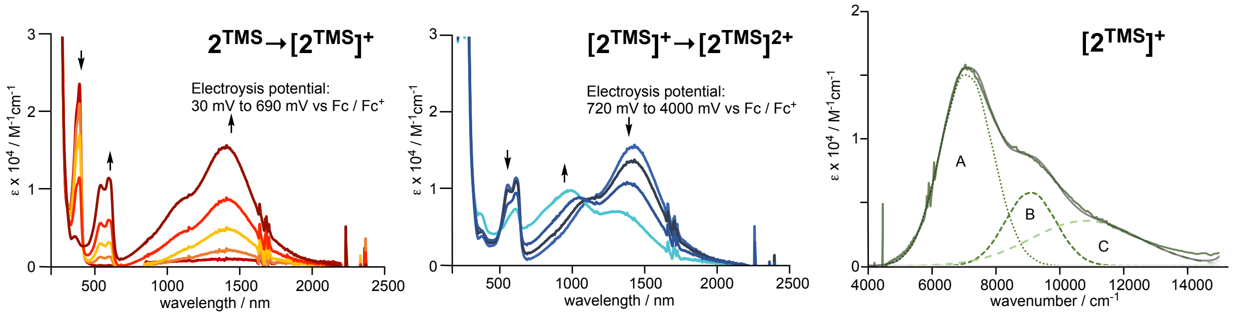

Figure S16. Absorption spectra of [$\mathbf{2^{TMS}}$]$^+$ and [$\mathbf{2^{TMS}}$]$^{2+}$ observed during the electrochemical oxidation and deconvoluted NIR bands for [$\mathbf{2^{TMS}}$]$^+$ (~0.1 mM in CH_2Cl_2 , 0.1M [n-Bu_4N][PF_6]).

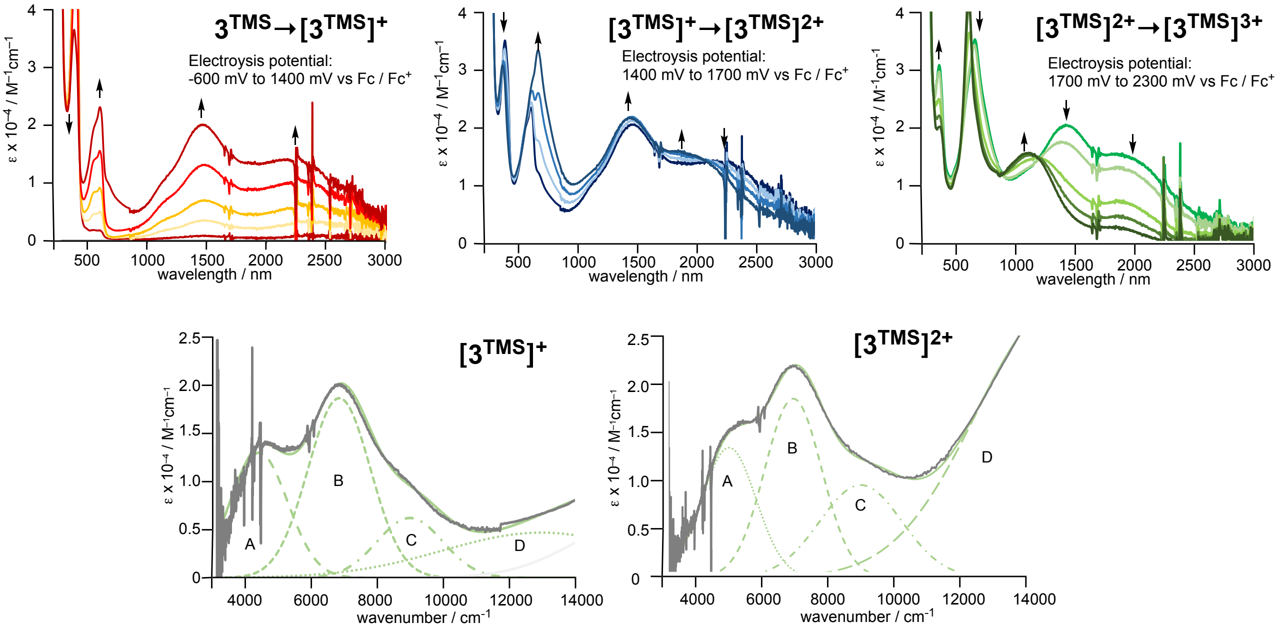

Figure S17. Absorption spectra of [$\mathbf{3^{TMS}}$]$^+$, [$\mathbf{3^{TMS}}$]$^{2+}$ and [$\mathbf{3^{TMS}}$]$^{3+}$ observed during the electrochemical oxidation and deconvoluted NIR bands for [$\mathbf{3^{TMS}}$]$^+$ and [$\mathbf{3^{TMS}}$]$^{2+}$ (~0.1 mM in CH_2Cl_2 , 0.1M [n-Bu_4N][PF_6]).

Table S2. Absorption spectral data of [**2**$^{\mathbf{Py}}$]$^{+}$, [**3**$^{\mathbf{TMS}}$]$^{+}$ and [**3**$^{\mathbf{TMS}}$]$^{2+}$ observed in the spectroelectrochemical condition.

complex	band	ν_{max} / cm^{-1}	$\nu_{1/2}^{exp.}$ / cm^{-}	ε_{max} / M^{-}	$\nu_{1/2}^{calcd.}$ /	Γ
[**2**$^{\mathbf{TMS}}$]$^{+}$	A	7081	1627	1.5×10^{4}	4044	0.60
	B	9086	1576	5.6×10^{3}	4581	0.66
	C	10810	3415	3.6×10^{3}	4997	0.32
[**3**$^{\mathbf{TMS}}$]$^{+}$	A	4408	1750	1.3×10^{4}		
	B	6850	1900	1.9×10^{4}		
	C	9000	2000	6.2×10^{3}		
	D	16000	4000	6.0×10^{3}		
[**3**$^{\mathbf{TMS}}$]$^{2+}$	A	5000	1638	1.3×10^{4}		
	B	6950	1730	1.8×10^{4}		
	C	9000	2500	9.6×10^{3}		
	D	15000	5000	2.8×10^{4}		

VI. Theoretical study

DFT study

DFT and TD-DFT calculations were performed by using the Gaussian 16 program package.[S14] Neutral complexes are optimized with the B3LYP/LanL2DZ (for Ru) and 6-31G(d) (for C, H, N, P) levels of theory, while open shell monocationic complexes are calculated at the UBLYP35/Def2SVP,CPCM(CH_2Cl_2) level of theory.[S13] Single point calculation and TD-DFT study were performed at the same level of theory.

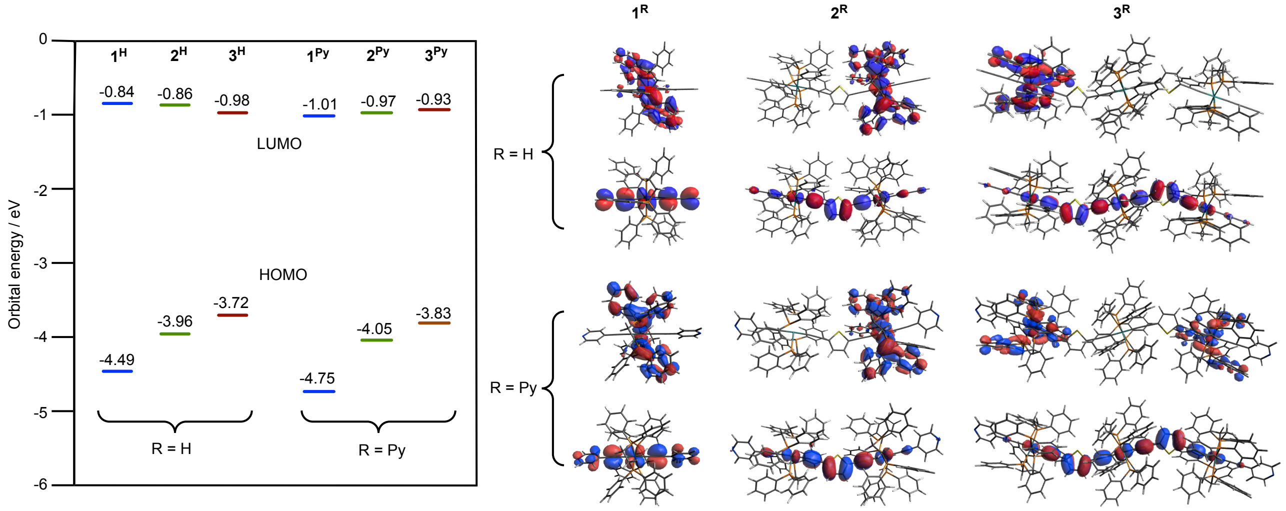

Figure S18. Energy diagrams and frontier orbitals for $\mathbf{1^R}$-$\mathbf{3^R}$ (R = H and Py). (top) LUMOs and (bottom) HOMOs.

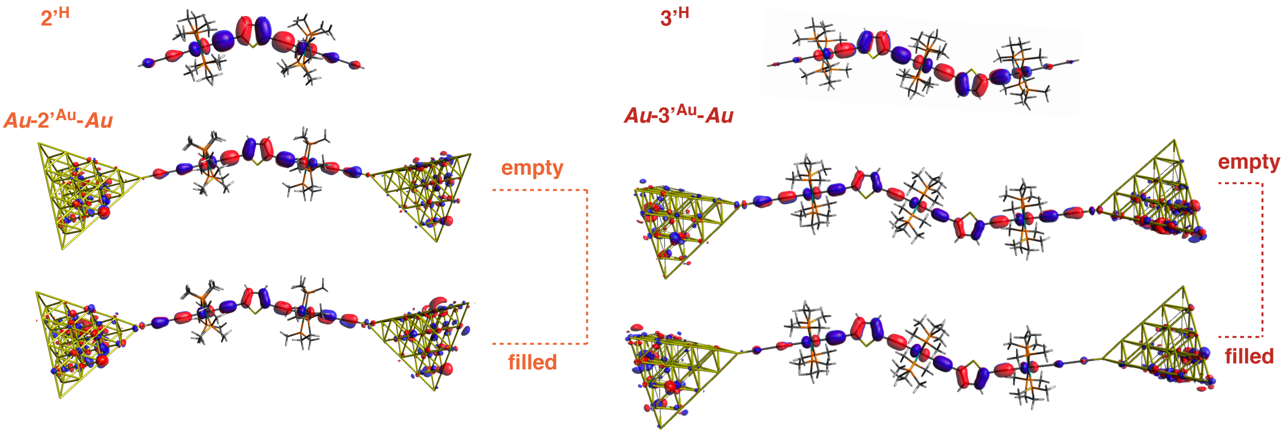

Figure S19. Comparisons of HOMO of $\boldsymbol{n'^{H}}$ and the conduction orbitals of $\boldsymbol{Au\text{-}\ n'^{Au}\text{-}Au}$ (n = 2, 3).

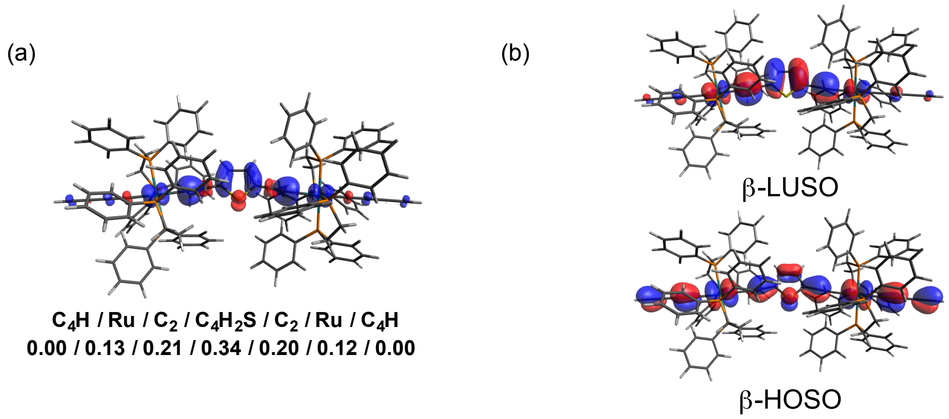

Figure S20. (a) Spin density map and spin densities, and (b) *β*-HOSO and *β*-LUSO of [**2^H**]$^+$

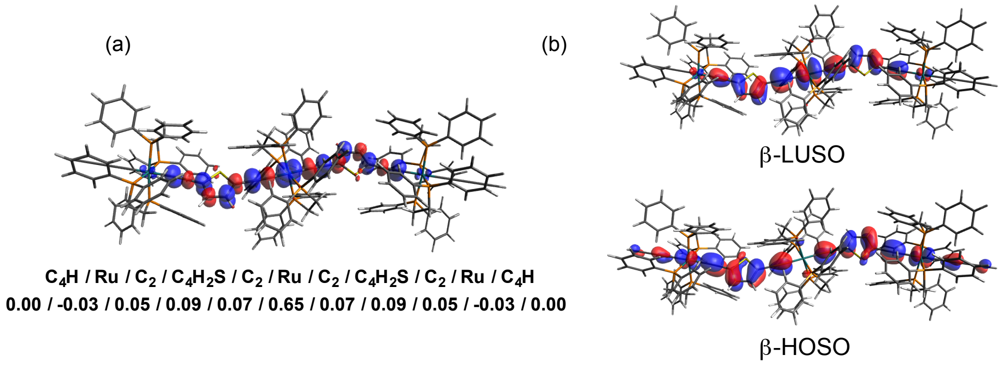

Figure S21. (a) Spin density map and spin densities, and (b) *β*-HOSO and *β*-LUSO of [**3^H**]$^+$

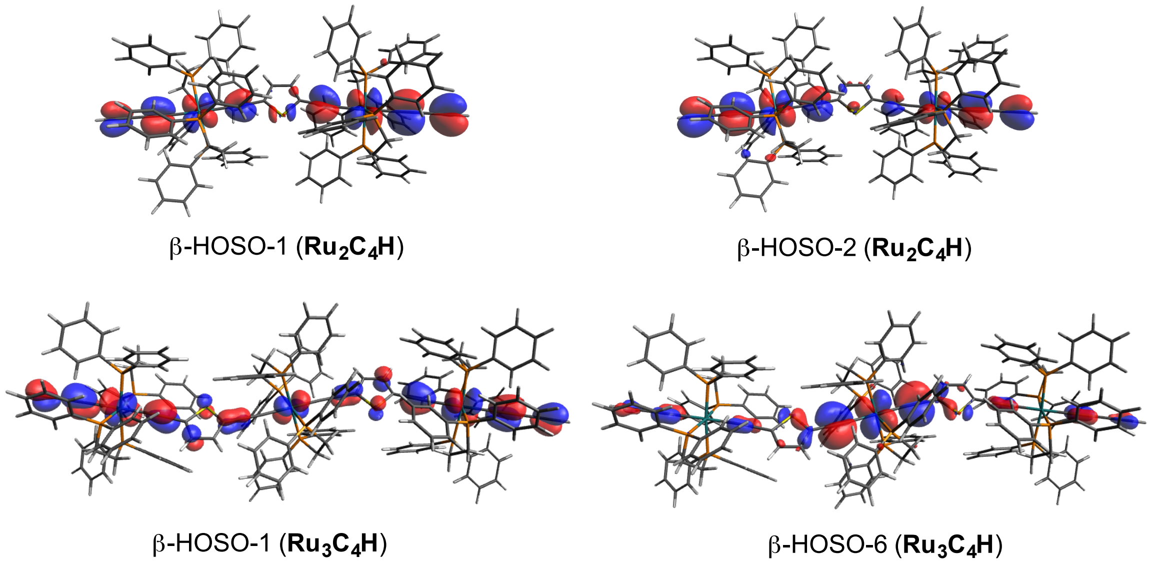

Figure S22. Selected spin orbitals of [**2^H**]$^+$ and [**3^H**]$^+$ related to NIR absorption bands.

Table S3. UV-Vis-NIR transition data obtained by TD-DFT calculation of [**2^H**]$^+$

band	ν / cm^{-1}	oscillator strength (*f*)	major contributions	assignment
band A	7918	0.9530	β-HOSO → β-LUSO	IVCT
band B	9184	0.0000	β-HOSO-1 → β-LUSO	Ru-C≡C→Ru-Ligand
band C	10327	0.0025	β-HOSO-2 → β-LUSO	Ru-C≡C→Ru-Ligand

Table S4. UV-Vis-NIR transition data obtained by TD-DFT calculation of [**3**$^{\mathbf{H}}$]$^{+}$

band	ν / cm^{-1}	oscillator strength (*f*)	major contributions	assignment
band A	5652	0.0000	β-HOSO-6 → β-LUSO	MLCT and d-d
band B	6975	1.0496	β-HOSO → β-LUSO	IVCT
band C	10322	0.0000	β-HOSO-1 → β-LUSO	Ru-C≡C→Ru-Ligand

DFT-NEGF study

DFT calculations for electron transport were performed by using the Gaussian 16 ad 09 program package [S14,15] and the non-equilibrium Green's function (NEGF) method in a level-broadening approach [S15]. For the molecular junction models, B3LYP/LanL2DZ (for Ru) and 6-31G(d) (for C, H, N, P) levels of theory were adopted for the transport calculations. The details of computational procedure in the level-broadening approach are described in elsewhere.[S16]

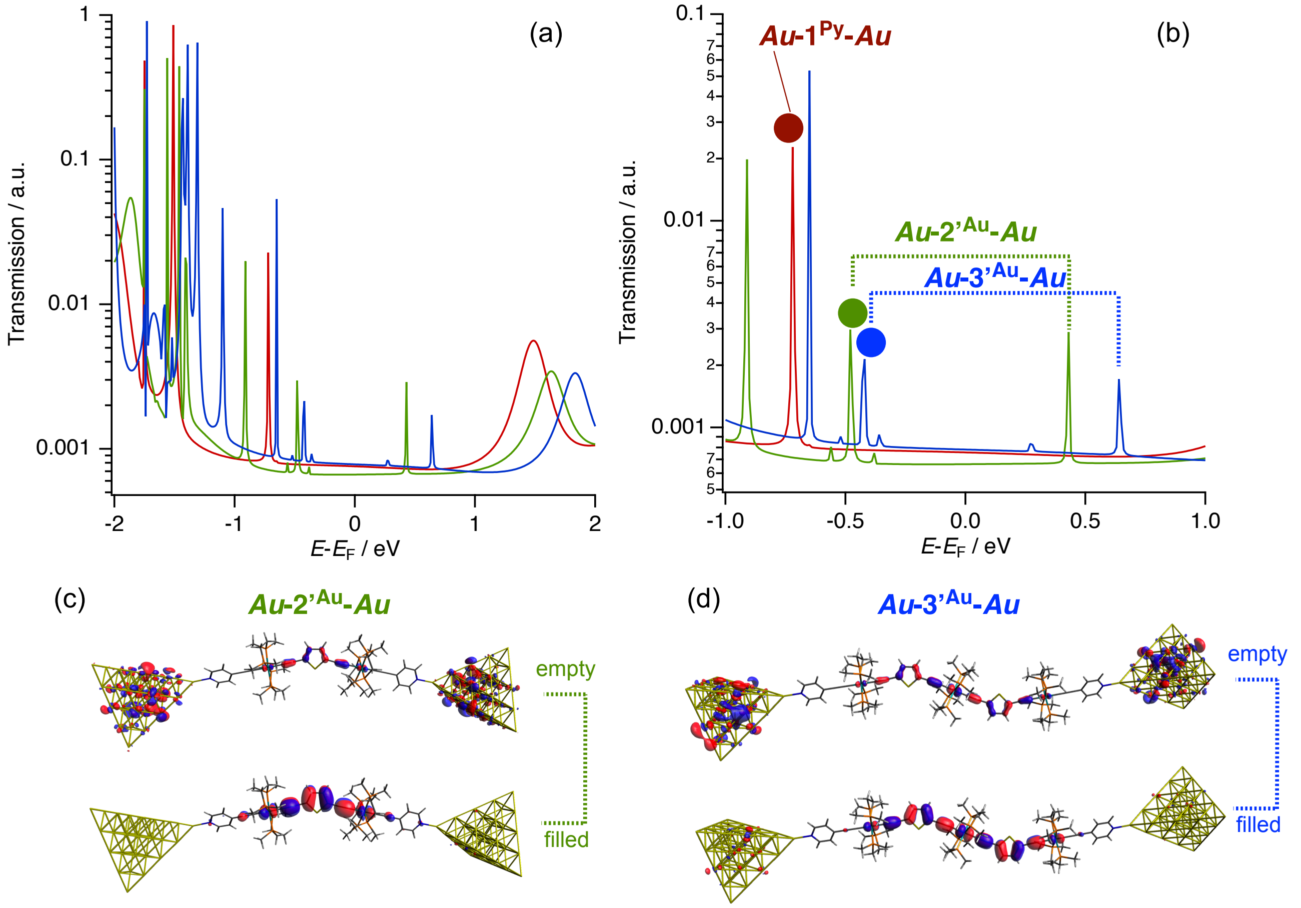

Figure S23. (a) Whole and (b) expanded transmission spectra for ***Au-n'*$^{\mathbf{Py}}$*-Au*** (n = 1-3) obtained by the DFT-NEGF method. (red) ***Au*-1'$^{\mathbf{Py}}$-*Au***; (green) ***Au*-2'$^{\mathbf{Py}}$-*Au***; (blue) ***Au*-3'$^{\mathbf{Py}}$-*Au***. Filled circles and dotted lines indicate HOMO conduction peaks and pairs of splitted conduction peaks derived from the HOMOs of the corresponding metal complexes, respectively. Conduction orbitals for (c) ***Au*-2'$^{\mathbf{Py}}$-*Au*** and (d) ***Au*-3'$^{\mathbf{Py}}$-*Au*** derived from the HOMOs of the corresponding metal complexes.

Table S5. Cartesian Coordinates of $\mathbf{2^H}$.

P	6.60375	-0.38814	-2.38130	C	10.55976	-2.76605	-3.11974
P	5.81717	2.38686	-0.95609	C	9.32215	-3.40469	-3.21745
P	6.07865	-2.14554	0.84150	C	8.14282	-2.68845	-3.00698
P	5.15845	0.64516	2.17500	C	4.30975	2.96045	-1.86354
P	-6.01389	-2.25778	-0.96751	C	4.37627	3.59658	-3.11509
P	-5.80172	0.70721	-2.19925	C	3.21823	4.03978	-3.75651
P	-6.01607	-0.89030	2.41349	C	1.97304	3.86078	-3.15292
P	-5.68821	2.09359	1.21167	C	1.89368	3.23814	-1.90675
C	5.51198	-1.20161	-3.63925	C	3.05060	2.78942	-1.26795
S	0.06394	0.21434	0.00784	C	6.26601	3.88180	0.04980
C	5.84990	-1.12843	-5.00401	C	7.36144	3.81716	0.92838
C	5.05650	-1.74585	-5.96829	C	7.74289	4.93670	1.66962
C	3.91603	-2.45729	-5.58367	C	7.03513	6.13534	1.55320
C	3.57957	-2.54374	-4.23431	C	5.94454	6.20782	0.68577
C	4.37061	-1.91863	-3.26441	C	5.56196	5.09104	-0.06088
C	8.17930	-1.31989	-2.69252	C	6.94339	1.25809	-3.24426
C	9.42969	-0.68799	-2.60066	C	7.14687	2.41332	-2.26212
C	10.60888	-1.40549	-2.81247	C	5.16518	-3.53770	0.01986
C	3.09881	-4.73191	-0.45628	C	3.77521	-3.67729	0.16114
C	3.79541	-5.65657	-1.23628	C	7.93581	1.11700	5.90235
C	5.17623	-5.52341	-1.38992	C	6.56409	1.36472	6.01000
C	5.85553	-4.47680	-0.76385	C	5.74364	1.26073	4.88820
C	7.66847	-3.00955	1.25034	C	5.27928	-2.09927	2.55169
C	7.66378	-4.09614	2.14578	C	4.31316	-0.92695	2.72399
C	8.84437	-4.76633	2.45904	C	-7.66502	-2.88886	-1.51316
C	10.05122	-4.36838	1.87566	C	-8.69578	-2.99041	-0.56539
C	10.06497	-3.30192	0.97898	C	-9.94869	-3.48727	-0.92420
C	8.88103	-2.62550	0.66668	C	-10.19892	-3.88474	-2.23851
C	3.87262	1.94194	2.47056	C	-9.18366	-3.79175	-3.19087
C	4.26368	3.25709	2.77583	C	-7.92629	-3.30226	-2.83006
C	3.31027	4.25140	2.99654	C	-5.21763	-3.78971	-0.28031
C	1.94921	3.95136	2.91620	C	-5.79740	-5.05865	-0.43790
C	1.54717	2.65096	2.60967	C	-5.16185	-6.19825	0.06037
C	2.50055	1.65411	2.38741	C	-3.93795	-6.08968	0.72208
C	6.28005	0.90882	3.63486	C	-3.35142	-4.83212	0.88138
C	7.65494	0.65577	3.54042	C	-3.98549	-3.69007	0.38841
C	8.47636	0.76185	4.66786	C	-7.23774	1.51714	-3.04788
C	-9.39834	2.62371	-2.99611	C	-8.32982	2.01492	-2.32910
C	-9.38770	2.74118	-4.38435	C	-4.75327	-1.70652	4.82737
C	-8.29860	2.25175	-5.11255	C	-3.67609	-1.85420	5.69936
C	-7.23169	1.64921	-4.44995	C	-2.38698	-1.50099	5.28903
C	-4.42947	1.82427	-2.76016	C	-2.18623	-0.99769	4.00489
C	-3.14609	1.32308	-3.03163	C	-3.26580	-0.84732	3.12784
C	-2.11919	2.17654	-3.44165	C	-6.93517	3.40379	0.79400
C	-2.35686	3.54484	-3.58312	C	-6.58604	4.48053	-0.03768
C	-3.62767	4.05547	-3.31403	C	-7.52589	5.45447	-0.37910
C	-4.65533	3.20258	-2.90829	C	-8.83150	5.37276	0.10768
C	-5.06985	-2.03566	-2.55892	C	-9.18981	4.31000	0.93850
C	-5.56804	-0.80174	-3.31441	C	-8.25173	3.33294	1.27742
C	-7.12336	-2.30946	2.84665	C	-4.15375	3.12992	1.30734
C	-8.49618	-2.13119	3.08815	C	-3.04944	2.88037	0.48530
C	-9.31396	-3.22350	3.38778	C	-1.90813	3.68540	0.57049
C	-8.77889	-4.51086	3.44554	C	-1.85660	4.74334	1.47651
C	-7.41718	-4.70069	3.20333	C	-2.95450	5.00077	2.30356
C	-6.59698	-3.61146	2.90807	C	-4.09393	4.20403	2.21605
C	-4.56088	-1.20413	3.52641	C	-6.79717	0.53901	3.32836
C	11.67184	1.31122	0.55041	C	-5.99856	1.81232	3.05176
C	10.48124	1.06843	0.45718	H	-0.96933	5.36874	1.53570

C	9.14818	0.79206	0.34198	H	-2.92613	5.82576	3.01084
C	7.94286	0.52838	0.22787	H	-4.94665	4.43188	2.84932
C	3.90758	-0.25719	-0.39293	H	1.34010	-2.65526	-1.80798
C	2.70284	-0.50370	-0.52124	H	-1.27743	-2.68634	-1.71004
C	1.33730	-0.81417	-0.67587	H	-7.81920	0.63814	2.95816
C	0.74827	-1.89127	-1.31722	H	-6.82934	0.32367	4.40226
C	-0.66781	-1.90579	-1.26661	H	-5.00879	1.74048	3.51539
C	-1.22883	-0.84032	-0.58276	H	-6.49946	2.68723	3.47915
C	-2.58706	-0.55682	-0.33877	H	7.80728	1.15154	-3.90829
C	-3.78505	-0.33612	-0.12416	H	6.06827	1.44963	-3.87281
C	-7.89399	0.06943	0.32464	H	7.18002	3.37768	-2.78039
C	-9.11303	0.14007	0.52545	H	8.08404	2.30287	-1.71067
C	-10.45429	0.22311	0.76829	H	4.77475	-3.05079	2.74862
C	-11.64801	0.29936	1.00391	H	6.10388	-2.00945	3.26665
Ru	5.93475	0.12733	-0.07444	H	3.99234	-0.83726	3.76796
Ru	-5.84562	-0.09181	0.11416	H	3.43387	-1.05677	2.09023
H	6.74518	-0.59891	-5.31776	H	-4.03076	-1.90783	-2.24531
H	5.33071	-1.67683	-7.01790	H	-5.12284	-2.93533	-3.18203
H	3.29663	-2.94093	-6.33475	H	-3.82553	5.11820	-3.42842
H	2.69505	-3.09458	-3.92704	H	-5.64313	3.61144	-2.72334
H	4.08774	-1.96847	-2.22129	H	-8.94614	-1.14832	3.02661
H	9.49670	0.36272	-2.34583	H	-10.37244	-3.06036	3.57220
H	11.56424	-0.89470	-2.72912	H	-9.41742	-5.35933	3.67730
H	11.47789	-3.32343	-3.28603	H	-6.98669	-5.69757	3.24034
H	9.27110	-4.46188	-3.46515	H	-5.53999	-3.77943	2.73195
H	7.18857	-3.19599	-3.10296	H	-5.74719	-1.99193	5.15923
H	5.33134	3.76290	-3.60170	H	-3.84324	-2.24605	6.69950
H	3.29345	4.52604	-4.72574	H	-1.54732	-1.61883	5.96935
H	1.07033	4.20480	-3.65143	H	-1.18917	-0.72117	3.67236
H	0.92915	3.07878	-1.43386	H	-3.09891	-0.46822	2.12616
H	2.97102	2.29176	-0.31034	H	-5.57091	4.56879	-0.41031
H	7.91472	2.89046	1.03194	H	-7.23290	6.28117	-1.02121
H	8.59461	4.86643	2.34098	H	-9.56259	6.13261	-0.15567
H	7.33231	7.00550	2.13298	H	-10.20315	4.23103	1.32275
H	5.38513	7.13469	0.58676	H	-8.56239	2.50669	1.90483
H	4.70841	5.16628	-0.72503	H	-3.07240	2.04919	-0.20731
H	3.20747	-2.95775	0.73889	H	-1.06186	3.47684	-0.07707
H	2.02388	-4.82300	-0.32578	H	-8.52543	-2.67179	0.45440
H	3.26741	-6.47649	-1.71610	H	-10.73166	-3.54726	-0.17373
H	5.73116	-6.24102	-1.98880	H	-11.17854	-4.26244	-2.51958
H	6.93234	-4.40200	-0.87335	H	-9.36485	-4.10109	-4.21699
H	6.73324	-4.43191	2.59433	H	-7.15294	-3.25651	-3.58939
H	8.82193	-5.60102	3.15506	H	-6.75073	-5.16329	-0.94373
H	10.97270	-4.89051	2.12058	H	-5.62891	-7.17130	-0.06943
H	10.99486	-2.98194	0.51773	H	-3.44492	-6.97750	1.10994
H	8.90909	-1.78718	-0.01491	H	-2.39856	-4.73266	1.39454
H	5.31604	3.50797	2.85358	H	-3.52443	-2.71775	0.52032
H	3.63791	5.26072	3.22984	H	-8.36017	1.90874	-1.25341
H	1.20539	4.72381	3.09355	H	-10.23831	2.99938	-2.41881
H	0.49122	2.40686	2.53596	H	-10.22070	3.21132	-4.90094
H	2.16032	0.65595	2.14044	H	-8.27866	2.34339	-6.19558
H	8.08846	0.39141	2.58365	H	-6.38403	1.29247	-5.02869
H	9.54110	0.56805	4.56963	H	-2.93152	0.26807	-2.91045
H	8.57533	1.20121	6.77735	H	-1.13361	1.76684	-3.64552
H	6.13191	1.64045	6.96864	H	-1.55960	4.20882	-3.90774
H	4.68150	1.46308	4.98826	H	-12.69462	0.36280	1.18892
H	-6.55110	-0.99797	-3.75250	H	12.70964	1.53156	0.64123
H	-4.88948	-0.54615	-4.13476				

Table S6. Cartesian Coordinates of $\mathbf{3^H}$.

Ru	-11.73583	-0.04933	-0.13153	H	8.88544	4.25563	5.18987
Ru	-0.02265	0.12109	0.07234	H	6.72442	3.99162	3.95051
P	-11.41156	-1.85609	-1.80675	H	6.67422	2.67504	1.82288
P	-12.33699	1.70196	1.54720	H	8.75130	1.63071	0.95217
P	-11.83196	-1.57903	1.82758	H	11.32860	4.43763	1.34288
P	-11.37034	1.46490	-2.08834	H	12.87636	6.36422	1.44385
P	0.57230	-1.66676	1.69423	H	15.23311	6.04947	2.22805
P	0.80061	1.58261	1.87846	H	16.01567	3.76371	2.89014
S	-5.84647	0.07991	-0.03939	H	14.48845	1.83719	2.76015
C	-14.75888	-4.31758	-2.43547	H	3.36909	-3.27942	-3.61320
C	-9.76680	-2.71808	-2.18386	H	2.71312	-2.99768	-6.01557
C	-13.96125	2.66070	1.39655	H	0.50562	-1.95995	-6.57553
C	-15.19242	1.97632	1.49978	H	-1.02535	-1.24826	-4.78087
C	-13.37804	-2.53113	2.37584	H	1.86025	-2.51624	-1.81197
C	-14.34208	-5.55972	-1.92179	H	-0.80592	4.44842	-3.16860
C	-8.63657	-2.52912	-1.36634	H	0.69833	5.54463	-4.79801
C	-12.59206	-3.33079	-1.87200	H	2.95841	4.58168	-5.27490
C	-13.97126	4.04745	1.13049	H	3.68135	2.49112	-4.09792
C	-11.60900	-0.40207	3.30905	H	2.17433	1.39349	-2.46571
C	-11.16053	3.06274	2.14146	H	-3.59165	2.30006	-1.17568
C	-9.74495	0.16409	0.38296	H	-4.95341	4.12693	-0.24127
C	-8.20347	-3.48723	2.73466	H	-3.85820	6.21590	0.60792
C	-14.47868	-2.69521	1.51334	H	-1.36284	6.41961	0.52198
C	-1.99964	0.26942	0.64985	H	0.00504	4.59188	-0.42258
C	-0.34081	2.06641	3.30059	H	-0.85789	-4.09603	-3.01682
C	-0.62673	-2.58865	2.84215	H	-2.16104	-6.15695	-2.59169
C	-10.17055	4.09135	-2.10847	H	-3.84675	-6.22485	-0.74226
C	-15.18784	4.74154	0.99553	H	-4.19898	-4.19376	0.67984
C	-9.19048	-2.50894	2.51361	H	-2.89442	-2.13114	0.25631
C	-7.41925	-3.16886	-1.67857	H	11.37759	1.33737	-4.16248
C	-9.93616	2.69880	-2.12356	H	10.28197	0.37573	-3.15018
C	-13.89270	-3.20848	-2.40703	H	11.90866	1.07703	4.36660
C	-7.32273	-4.00191	-2.80636	H	13.11847	0.22431	3.37690
C	-11.73055	-0.98501	-3.47080	H	-3.01900	-0.26729	-1.83683
C	-0.83915	2.31898	5.68688	H	-2.58926	-0.82853	-3.45586
C	-11.49839	3.83748	3.27712	H	11.96573	-1.13145	-4.29199
C	-13.71592	-0.22408	-0.64018	H	13.28761	-0.20708	-3.56010
C	-10.79081	-4.25534	1.95837	H	11.44564	-1.41776	4.32559
C	-12.18063	-4.57951	-1.35482	C	2.29767	4.09760	-4.55894
C	-2.74509	-2.65844	4.06033	C	8.85037	3.68076	4.26710
C	-13.43164	-3.09577	3.67300	C	8.80500	5.30797	-1.87066
C	-16.40848	2.67109	1.36813	C	7.47660	-3.12694	2.11400
C	-4.58392	0.47071	1.24496	C	-3.09455	3.20235	-0.83500
C	-10.49650	-2.88437	2.13012	C	17.49578	0.04458	1.38839
C	2.16907	0.54570	2.70776	C	-3.87382	4.24380	-0.29654
C	1.73564	3.20054	1.56012	H	12.13345	4.63654	-1.47530
C	-13.98898	2.66377	-2.28389	H	10.56016	6.54330	-1.55688
C	-12.51728	3.07180	-4.17675	H	8.11445	6.14731	-1.91282
C	-1.89350	-2.03852	3.12499	H	7.27073	3.80098	-2.16321
C	-9.94627	3.31641	1.47748	H	8.83056	1.89544	-2.04379
C	-12.74186	2.48196	-2.90943	H	12.98403	-4.55006	-1.78081
C	-15.62046	-3.40239	1.94246	H	15.17099	-5.69813	-1.90401
C	-12.61835	0.74725	3.18401	H	17.27762	-4.37387	-2.18007
C	-9.66731	-3.56437	-3.31462	H	17.15659	-1.87421	-2.28278
C	-1.59521	2.63934	2.99675	H	14.98302	-0.72474	-2.13035
C	-14.57110	-3.79673	4.10207	H	9.57501	-2.73719	-0.78957
C	-14.91533	-0.32433	-0.98674	H	7.99385	-4.46378	-1.58870
C	-10.89000	0.29784	-3.52846	H	8.41806	-5.66166	-3.74714

C	-15.67332	-3.95067	3.23567
C	-3.21222	0.37042	0.94000
C	-8.55902	0.31454	0.75139
C	1.62824	-3.08263	1.03453
C	-14.99970	3.41714	-2.91443
C	0.02722	1.91104	4.65494
C	-9.07883	4.32573	1.94195
C	3.21745	5.56454	1.07483
C	-6.66270	0.82963	2.36190
C	-9.41583	5.08888	3.07298
C	1.67033	-0.86246	3.06351
C	-16.23483	-0.43849	-1.34873
C	-10.63295	4.84208	3.74140
C	-7.76911	4.52553	-2.14960
C	2.69187	3.24418	0.52089
C	1.00306	-4.25035	0.54174
C	1.77061	-5.29505	-0.00369
C	-5.23098	0.83718	2.41975
C	-9.09489	4.99838	-2.12391
C	-1.07181	-4.37968	4.45145
C	-9.80125	-5.23184	2.17457
C	-8.60374	2.23128	-2.12593
C	-13.04892	-5.68567	-1.37930
C	2.26222	5.52627	2.10837
C	3.42880	4.41892	0.28355
C	-2.08275	2.90053	5.37552
C	-13.52532	3.82020	-4.80702
C	-7.21307	0.45347	1.14097
C	-2.45583	3.06020	4.02744
C	-0.21957	-3.76209	3.51833
C	-16.41161	4.05689	1.11916
C	3.17286	-5.18270	-0.07464
C	1.52578	4.35078	2.35150
C	-14.77450	3.99401	-4.17589
C	-2.34002	-3.83026	4.72502
C	-8.45401	-4.20080	-3.62514
C	-8.50356	-4.85192	2.56641
C	-7.52853	3.13819	-2.14566
C	3.03206	-2.97278	0.95192
C	-17.42075	-0.55067	-1.67395
C	3.80042	-4.01647	0.40224
H	-11.79126	-4.56665	1.67303
H	-10.04658	-6.28317	2.04148
H	-7.73923	-5.60694	2.73644
H	-7.20365	-3.17569	3.02739
H	-8.92523	-1.46353	2.61822
H	-13.03683	4.59221	1.03397
H	-15.17634	5.80996	0.79256
H	-17.35255	4.59279	1.01689
H	-17.34515	2.12468	1.44287
H	-15.21691	0.90283	1.65484
H	-9.66289	2.71776	0.62247
H	-8.14305	4.50088	1.41784
H	-8.74350	5.86523	3.43164
H	-10.90813	5.43033	4.61392
H	-12.44472	3.67974	3.78977
H	-12.58056	-3.01235	4.34490
H	-14.59675	-4.22454	5.10176
H	-16.55607	-4.49362	3.56625
H	-16.46025	-3.51579	1.26173
H	10.47076	-5.11785	-5.07572
H	12.07170	-3.42494	-4.25221
H	12.68676	2.99188	-4.27520
H	14.77570	4.09006	-4.99836
H	16.71092	4.28313	-3.42208
H	16.51450	3.34361	-1.10726
H	14.43579	2.20538	-0.39785
H	8.51434	-1.24215	2.11130
H	6.50332	-2.65620	2.22518
H	6.69411	-5.14994	2.05114
H	8.94975	-6.19027	1.73158
H	10.97542	-4.77474	1.65224
H	11.73687	-3.63561	4.19752
H	13.51567	-5.17245	4.95456
H	15.60247	-5.45129	3.59792
H	15.87743	-4.14651	1.47717
H	14.11778	-2.57392	0.74639
H	10.95927	3.23381	4.30524
C	1.54997	-2.40933	-2.84482
C	14.70593	3.67911	-3.99373
C	14.95934	-0.01905	0.78619
C	11.18970	-0.77988	3.47404
C	15.79450	3.79065	-3.10447
C	3.16389	-0.13147	-0.80770
C	8.51645	-0.24984	-0.72441
C	-1.69005	3.31676	-0.89783
C	14.97630	-4.04064	2.07540
C	-0.06632	-1.67901	-4.50805
C	8.88146	-4.24194	-2.17525
C	-3.27465	-5.31877	-0.92939
C	6.57257	-0.61710	-2.31601
C	9.11926	-4.91163	-3.38779
C	-1.70477	1.09891	-2.92731
C	16.29550	0.00877	1.10105
C	10.27322	-4.60378	-4.13780
C	7.58441	-4.52667	2.00406
C	-2.73426	-3.00342	-0.36852
C	-1.07549	4.48550	-0.39451
C	-1.85383	5.52733	0.14114
C	5.14095	-0.57840	-2.34485
C	8.85295	-5.11147	1.83029
C	1.02586	4.64058	-4.28936
C	10.18023	5.53024	-1.66933
C	8.62774	-2.32052	2.06283
C	13.22910	5.37768	1.73591
C	-2.32597	-5.28024	-1.96909
C	-3.47534	-4.17571	-0.13146
C	2.04314	-2.67457	-5.22190
C	13.64882	-4.61046	4.03293
C	7.15791	-0.31953	-1.08996
C	2.41242	-2.83336	-3.87266
C	0.17122	4.01529	-3.36352
C	16.31602	-3.86872	-2.12355
C	-3.25673	5.41144	0.19048
C	-1.58578	-4.10702	-2.21179
C	14.82195	-4.76986	3.26671
C	12.78539	2.99063	2.05148
C	13.88701	-3.95901	-1.90024
C	11.32176	0.68926	-3.28186
C	10.94034	-2.95633	-2.45067

H	-14.45713	-2.26594	0.52034	C	9.71719	-0.17003	-0.38175
H	-8.39076	1.16813	-2.08744	C	8.33114	3.99029	-2.01472
H	-6.51167	2.75429	-2.14352	C	14.50019	2.62112	-1.39480
H	-6.93688	5.22579	-2.17064	C	1.95354	-0.02756	-0.50888
H	-9.29473	6.06741	-2.11558	C	0.29787	-1.83350	-3.15253
H	-11.18671	4.47423	-2.09378	C	0.57953	2.84021	-2.69101
H	-11.55196	2.97221	-4.66815	C	10.00553	-4.30488	1.78459
H	-13.33603	4.26752	-5.78021	C	15.13124	-4.61326	-1.97067
H	-15.55740	4.57074	-4.66340	C	9.22228	2.90277	-1.96010
H	-15.95784	3.53832	-2.41617	C	7.60433	2.79596	2.37209
H	-14.18722	2.20538	-1.32485	C	9.90633	-2.90282	1.91708
H	-10.53672	-3.74746	-3.94217	C	14.11740	2.81814	2.48685
H	-8.39244	-4.84940	-4.49621	C	7.63388	3.53341	3.56835
H	-6.38019	-4.49003	-3.04421	C	12.05885	0.48474	3.45752
H	-6.55304	-3.00378	-1.04396	C	0.80163	-2.09061	-5.53720
H	-8.69941	-1.88028	-0.50107	C	11.17599	-3.63369	-3.67132
H	-11.17983	-4.69822	-0.94978	C	13.74517	-0.03767	0.48015
H	-12.71391	-6.64051	-0.98033	C	11.07458	4.44474	-1.61909
H	-15.01350	-6.41515	-1.94307	C	12.35112	4.28068	1.67341
H	-15.75729	-4.20164	-2.84992	C	2.70392	2.92398	-3.89814
H	-14.24910	-2.25782	-2.78643	C	13.52239	3.04266	-3.58175
H	-3.41370	3.50583	3.77132	C	16.24812	-2.46398	-2.19183
H	-2.75129	3.22130	6.17136	C	4.52736	-0.25058	-1.14096
H	-0.54029	2.18758	6.72430	C	10.60509	3.12088	-1.76867
H	0.98808	1.48231	4.92452	C	-2.20931	-0.30670	-2.56998
H	-1.90845	2.74644	1.96473	C	-1.78507	-2.95935	-1.41401
H	0.75507	-4.19965	3.32066	C	13.96950	-3.15166	1.64802
H	-0.74515	-5.28248	4.96285	C	12.64412	-3.72691	3.60456
H	-2.99858	-4.30792	5.44731	C	1.84973	2.29619	-2.97048
H	-3.71927	-2.21995	4.26372	C	9.78632	-3.26705	-1.70846
H	-2.21761	-1.13730	2.61716	C	12.79608	-2.98743	2.40670
H	3.53705	-2.07218	1.28521	C	15.68519	3.26437	-1.80534
H	4.87893	-3.90132	0.32918	C	12.24793	-0.52447	-3.42597
H	3.76572	-5.98872	-0.50133	C	10.02397	3.09112	3.76853
H	1.27153	-6.18620	-0.37656	H	10.12816	-0.52148	3.55255
H	-0.07729	-4.35337	0.58449	H	7.17814	-0.87151	-3.17980
H	0.79296	4.34008	3.15206	H	-1.05903	1.06052	-3.81081
H	2.08980	6.40479	2.72635	H	-2.53621	1.77486	-3.15676
H	3.78710	6.47220	0.88832	H	4.55555	-0.79069	-3.23298
H	4.15874	4.43616	-0.52199	H	18.53080	0.06017	1.63992
H	2.85949	2.37029	-0.09988	H	-18.44298	-0.63609	-1.96129
H	-11.75444	-0.95409	4.24327	P	11.59001	1.53239	1.93791
H	-10.58224	-0.02457	3.28371	P	12.16041	-1.63382	-1.86013
H	-11.47023	-1.66349	-4.29003	P	11.80852	1.66037	-1.71904
H	-12.79788	-0.75170	-3.53616	P	11.43565	-1.78682	1.86140
H	2.97527	0.50936	1.97058	P	-0.61978	1.90844	-1.55120
H	2.55132	1.06609	3.59353	P	-0.84557	-1.34384	-1.73381
H	-12.52042	1.44741	4.01949	S	5.82583	0.04043	0.13457
H	-13.64424	0.36342	3.18288	C	14.99349	3.91779	2.55323
H	-11.04555	0.82426	-4.47519	C	9.99776	2.34285	2.56729
H	-9.82256	0.06745	-3.44144	C	13.81054	-2.55213	-1.98706
H	-7.29293	1.10792	3.20048	C	15.00540	-1.80991	-2.11676
H	1.03379	-0.82947	3.95382	C	13.41121	2.49883	-2.28003
H	2.50549	-1.53790	3.28062	C	14.55436	5.20107	2.17753
H	-4.67058	1.10775	3.30818	C	8.78197	2.20306	1.87181
Ru	11.73703	-0.08011	0.05600				

Table S7. Cartesian Coordinates of $\mathbf{2^{Py}}$.

P	6.59840	0.70524	2.42547	H	-9.39415	5.28777	-3.77164
P	5.85980	-2.19805	1.25323	H	-6.96729	5.63716	-3.32296
P	6.02464	2.17691	-0.91954	H	-5.51906	3.72928	-2.78107
P	5.18136	-0.73353	-2.02165	H	-5.70924	1.90598	-5.18475
P	-6.01678	2.25624	0.92882	H	-3.79597	2.14572	-6.71537
P	-5.81760	-0.69051	2.20749	H	-1.50301	1.53623	-5.96157
P	-5.99311	0.84178	-2.42599	H	-1.15740	0.67015	-3.65042
P	-5.66342	-2.12133	-1.18540	H	-3.07665	0.43117	-2.11389
C	5.49722	1.61925	3.60517	H	-5.54392	-4.57787	0.46978
S	0.07723	-0.19606	0.04021	H	-7.20166	-6.29220	1.08686
C	5.83508	1.67182	4.97093	H	-9.52476	-6.16768	0.20027
C	5.03966	2.37165	5.87525	H	-10.16404	-4.28848	-1.30630
C	3.89830	3.04349	5.42735	H	-8.52395	-2.56139	-1.89568
C	3.56189	3.00539	4.07582	H	-3.08038	-2.06547	0.28978
C	4.35313	2.29434	3.16714	H	-1.07252	-3.50159	0.22529
C	8.16121	1.67844	2.67732	H	-0.94371	-5.40428	-1.37335
C	9.41552	1.05139	2.73474	H	-2.85656	-5.85764	-2.90502
C	10.57600	1.79733	2.95712	H	-4.87595	-4.45647	-2.80720
C	10.50673	3.18362	3.10400	H	1.29731	2.71216	1.83323
C	9.26609	3.81945	3.03566	H	-1.31859	2.70978	1.70603
C	8.10457	3.07326	2.83119	H	-7.78612	-0.69911	-2.95320
C	4.35479	-2.70886	2.20093	H	-6.79781	-0.39993	-4.40357
C	4.41950	-3.21114	3.51187	H	-4.97009	-1.79628	-3.49043
C	3.26347	-3.61453	4.18265	H	-6.45636	-2.74979	-3.45101
C	2.02288	-3.53120	3.54944	H	7.79643	-0.67536	4.10484
C	1.94570	-3.04422	2.24396	H	6.06401	-1.00061	4.06443
C	3.10031	-2.63329	1.57603	H	7.23714	-2.99697	3.15974
C	6.33858	-3.77929	0.40533	H	8.12002	-1.99656	2.00125
C	7.44878	-3.78532	-0.45596	H	4.67352	2.89343	-2.87278
C	7.86298	-4.96749	-1.07148	H	6.03064	1.86000	-3.33011
C	7.17151	-6.16016	-0.84642	H	3.95805	0.58210	-3.71496
C	6.06488	-6.16251	0.00354	H	3.40463	0.91570	-2.05344
C	5.65088	-4.98269	0.62635	H	-4.04662	1.92591	2.22996
C	6.94753	-0.85104	3.43646	H	-5.14768	2.96644	3.14147
C	7.18361	-2.08185	2.55999	H	-6.57707	1.03737	3.73163
C	5.08496	3.61338	-0.21033	H	-4.91781	0.59147	4.13006
C	3.68830	3.69411	-0.33254	C	10.50376	-1.00038	-0.67611
C	2.98763	4.77785	0.20153	C	10.81236	-1.96007	-1.66712
C	3.66707	5.79390	0.87597	H	6.83844	4.61680	0.55550

C	5.05467	5.72221	1.00754	H	6.62555	4.32897	-2.85509
C	5.75756	4.64449	0.46565	H	8.68943	5.48744	-3.52179
C	7.59449	3.03548	-1.40913	H	10.85868	4.89972	-2.44953
C	7.56475	4.04708	-2.38786	H	10.92694	3.12602	-0.70372
C	8.73100	4.71150	-2.76170	H	8.86279	1.93556	-0.06282
C	9.94817	4.38295	-2.15721	H	5.43194	-3.64370	-2.39764
C	9.98677	3.39118	-1.17905	H	3.81201	-5.47383	-2.62930
C	8.81743	2.72021	-0.80560	H	1.36417	-4.99783	-2.60243
C	3.93811	-2.08970	-2.20628	H	0.57303	-2.65848	-2.29798
C	4.37170	-3.41713	-2.36709	H	2.18349	-0.82768	-2.04882
C	3.45158	-4.45630	-2.50657	H	8.04638	-0.20206	-2.53791
C	2.08144	-4.18945	-2.48669	H	9.48483	-0.43282	-4.52877
C	1.63675	-2.87732	-2.32231	H	8.58082	-1.44663	-6.61764
C	2.55724	-1.83549	-2.18235	H	6.20949	-2.20339	-6.68776
C	6.30389	-1.06163	-3.46933	H	4.77114	-1.96154	-4.70754
C	7.63748	-0.62906	-3.44526	H	-8.50001	2.69239	-0.53355
C	8.45206	-0.76740	-4.57418	H	-10.70538	3.60336	0.05505
C	7.94619	-1.33623	-5.74210	H	-11.18393	4.32380	2.39193
C	6.61597	-1.76263	-5.78099	H	-9.40313	4.13694	4.12176
C	5.80161	-1.62452	-4.65765	H	-7.19104	3.26569	3.53247
C	5.21163	1.97613	-2.61282	H	-6.73773	5.16686	0.87809
C	4.28264	0.76397	-2.68414	H	-5.60327	7.16022	-0.01140
C	-7.67158	2.90118	1.44857	H	-3.41832	6.94409	-1.18509
C	-8.68500	3.01343	0.48315	H	-2.38355	4.69110	-1.44733
C	-9.93714	3.52844	0.81930	H	-3.52175	2.69044	-0.55712
C	-10.20537	3.93051	2.12931	H	-8.32574	-1.98279	1.25092
C	-9.20849	3.82386	3.09926	H	-10.21277	-3.06399	2.41296
C	-7.95083	3.31876	2.76015	H	-10.25844	-3.16815	4.90123
C	-5.21150	3.77818	0.22915	H	-8.36947	-2.20505	6.20718
C	-5.78425	5.05183	0.37485	H	-6.46687	-1.16356	5.04660
C	-5.14144	6.18352	-0.13201	H	-2.95247	-0.24116	2.92743
C	-3.91693	6.06242	-0.79042	H	-1.15960	-1.72654	3.69907
C	-3.33698	4.80037	-0.93725	H	-1.58784	-4.16432	4.00225
C	-3.97834	3.66613	-0.43564	H	-3.85337	-5.07971	3.53411
C	-7.26213	-1.48771	3.05527	H	-5.66660	-3.58538	2.79242
C	-8.32367	-2.04096	2.33064	H	-8.91382	1.08638	-3.06323
C	-9.39706	-2.64457	2.99486	H	-10.34485	2.98862	-3.64479
C	-9.42178	-2.70211	4.38698	C	9.15723	-0.68097	-0.34056
C	-8.36280	-2.15904	5.12113	C	7.96674	-0.41592	-0.13957
C	-7.29105	-1.56166	4.46110	C	3.91241	0.34417	0.47468

C	-4.45124	-1.79885	2.79982
C	-3.16874	-1.29341	3.06801
C	-2.14442	-2.13985	3.49850
C	-2.38362	-3.50524	3.66423
C	-3.65406	-4.01956	3.40025
C	-4.67932	-3.17370	2.97404
C	-5.08856	2.05754	2.53239
C	-5.59164	0.83460	3.30211
C	-7.09828	2.25426	-2.88597
C	-8.46887	2.07082	-3.13428
C	-9.28815	3.15622	-3.45392
C	-8.75536	4.44400	-3.52441
C	-7.39565	4.63982	-3.27567
C	-6.57453	3.55653	-2.96133
C	-4.53142	1.14392	-3.53344
C	-4.71675	1.62818	-4.84230
C	-3.63426	1.76779	-5.70902
C	-2.34679	1.42449	-5.28536
C	-2.15304	0.93890	-3.99335
C	-3.23794	0.79655	-3.12157
C	-6.90422	-3.43565	-0.76057
C	-6.55612	-4.50012	0.08703
C	-7.49376	-5.47499	0.43238
C	-8.79580	-5.40693	-0.06598
C	-9.15338	-4.35642	-0.91275
C	-8.21713	-3.37886	-1.25492
C	-4.12274	-3.15053	-1.24861
C	-3.04079	-2.90013	-0.39760
C	-1.90061	-3.70990	-0.44569
C	-1.82826	-4.77295	-1.34441
C	-2.90226	-5.02939	-2.20253
C	-4.04071	-4.22820	-2.15123
C	-6.76514	-0.60102	-3.32691
C	-5.96203	-1.86743	-3.03135
C	11.61089	-0.37796	-0.05937
C	12.14220	-2.23788	-1.96708
H	10.01380	-2.46654	-2.20028
C	12.90131	-0.73425	-0.43913
H	11.45244	0.37819	0.70188
H	12.38068	-2.97797	-2.73088

C	2.70142	0.56642	0.58697
C	1.32998	0.85819	0.72328
C	0.72068	1.93593	1.34399
C	-0.69451	1.93151	1.27906
C	-1.23489	0.85001	0.60380
C	-2.58794	0.55004	0.35135
C	-3.78372	0.32369	0.13062
C	-7.89974	-0.09344	-0.32052
C	-9.12245	-0.17692	-0.46959
Ru	5.94580	-0.01591	0.17942
Ru	-5.84166	0.07535	-0.12064
H	6.73219	1.17658	5.33182
H	5.31369	2.39870	6.92680
H	3.27857	3.59354	6.13090
H	2.67882	3.52814	3.71936
H	4.06970	2.24576	2.12433
H	9.49743	-0.02174	2.60246
H	11.53490	1.28924	3.02037
H	11.41046	3.76154	3.27775
H	9.19754	4.89747	3.15604
H	7.14515	3.57974	2.81182
H	5.37175	-3.30358	4.02324
H	3.33676	-3.99598	5.19781
H	1.12229	-3.84445	4.07166
H	0.98434	-2.96265	1.74504
H	3.02214	-2.24081	0.57051
H	7.98279	-2.86008	-0.64274
H	8.72899	-4.95345	-1.72816
H	7.49428	-7.07968	-1.32785
H	5.51819	-7.08415	0.18649
H	4.78663	-5.00519	1.28033
H	3.13478	2.90533	-0.82751
H	1.90755	4.81923	0.08990
H	3.12050	6.63674	1.29089
H	5.59611	6.51116	1.52324
C	-12.56980	-0.77917	-1.79749
H	-10.59675	-0.95591	-2.63785
C	-12.76349	-0.08490	0.36054
H	-10.95957	0.36431	1.45293
H	-13.06745	-1.10237	-2.71198

H	13.75647	-0.25422	0.03687	H	-13.42082	0.15428	1.19638
C	-10.53526	-0.27763	-0.57352	N	13.19228	-1.64979	-1.37477
C	-11.18106	-0.69716	-1.75889	N	-13.37438	-0.48309	-0.76672
C	-11.38597	0.03413	0.51164				

Table S8. Cartesian Coordinates of **3**^{Py}.

Ru	-11.62105	0.04773	0.05498	H	11.06464	4.51056	0.99384
Ru	0.00000	0.00019	-0.00016	H	12.62758	6.40583	1.18067
P	-11.24365	-1.64394	-1.67146	H	14.88048	6.07786	2.18944
P	-12.28198	1.69596	1.74142	H	15.54998	3.81507	2.98836
P	-11.63983	-1.51065	1.93819	H	14.01327	1.91642	2.76410
P	-11.28724	1.62475	-1.78276	H	3.02141	-3.60670	-3.68822
P	0.51807	-1.79105	1.58760	H	2.37505	-3.21620	-6.06463
P	0.95430	1.37966	1.74746	H	0.26962	-1.99116	-6.57744
S	-5.76914	0.16958	0.08753	H	-1.17470	-1.19740	-4.76186
C	-14.56863	-3.97284	-2.54959	H	1.60881	-2.75584	-1.86363
C	-9.62639	-2.50339	-1.98407	H	-0.54398	4.34705	-3.15068
C	-13.80104	2.72981	1.50242	H	0.97385	5.29614	-4.83658
C	-15.08395	2.16688	1.59603	H	3.08919	4.12287	-5.42561
C	-13.05741	-2.63981	2.35553	H	3.64927	1.96544	-4.30553
C	-14.19421	-5.23971	-2.09906	H	2.12913	1.01470	-2.61332
C	-8.57797	-2.45335	-1.05810	H	-3.40985	2.38901	-1.11577
C	-12.41401	-3.06723	-1.87058	H	-4.58353	4.29369	-0.11470
C	-13.69010	4.09007	1.16826	H	-3.30757	6.27544	0.69619
C	-11.62813	-0.39721	3.43784	H	-0.82393	6.30261	0.49067
C	-11.14390	2.95607	2.50177	H	0.35757	4.39939	-0.52042
C	-9.60719	0.29388	0.51502	H	-1.20684	-4.07117	-3.07171
C	-7.92173	-3.04933	2.96318	H	-2.57826	-6.05398	-2.57313
C	-14.03942	-2.95540	1.40897	H	-4.10418	-6.06341	-0.60546
C	-1.96542	0.29154	0.61868	H	-4.24157	-4.04468	0.85193
C	-0.10448	1.91771	3.16552	H	-2.87615	-2.05186	0.34423
C	-0.68657	-2.61041	2.74584	H	11.79551	0.97041	-4.35405
C	-10.43023	4.31269	-1.47832	H	10.63856	-0.06645	-3.50781
C	-14.82809	4.86623	0.94476	H	11.12924	1.32071	4.12378
C	-8.99414	-2.17892	2.75810	H	12.50555	0.46941	3.40593
C	-7.36935	-3.10977	-1.31748	H	-3.02246	-0.19467	-1.74832
C	-10.02759	2.97841	-1.65620	H	-2.72228	-0.78043	-3.38729
C	-13.68870	-2.89472	-2.43341	H	12.77900	-1.31628	-4.13695
C	-7.19464	-3.82395	-2.50079	H	13.68177	-0.20911	-3.09673
C	-11.44487	-0.70613	-3.27494	H	10.65356	-1.13608	4.12741
C	-0.56290	2.16790	5.54553	H	9.57307	-0.34342	2.97553
C	-11.55154	3.65741	3.65364	H	7.14799	-1.39142	-3.21422
C	-13.63893	-0.18590	-0.37381	H	-1.07770	0.99664	-3.80221
C	-10.32821	-4.02163	1.95119	H	-2.48026	1.79874	-3.10784
C	-12.04868	-4.34786	-1.42192	C	7.70988	-3.71696	1.58601
C	-2.73233	-2.49271	4.05562	C	-2.83209	3.24509	-0.78805
C	-13.13892	-3.23062	3.63126	C	-3.50158	4.32710	-0.21186
C	-16.22201	2.94529	1.37650	H	11.26558	4.41643	-1.57073
C	-4.51154	0.60839	1.25515	H	9.36553	5.94436	-1.91545
C	-10.21463	-2.65353	2.25151	H	7.21210	5.08290	-2.82142
C	2.25842	0.29255	2.52278	H	6.98720	2.65763	-3.35364
C	1.94158	2.91341	1.39861	H	8.86019	1.12649	-2.97181
C	-13.94793	2.60145	-2.12846	H	12.71035	-4.55158	-1.09218
C	-12.39548	3.17801	-3.88388	H	14.71951	-5.91742	-0.68903
C	-1.87185	-1.94866	3.09790	H	16.98540	-4.90191	-0.88021
C	-9.89009	3.24600	1.95196	H	17.20407	-2.48693	-1.45109
C	-12.65570	2.52844	-2.66125	H	15.20408	-1.11347	-1.81592
C	-15.08641	-3.82610	1.73217	H	9.55206	-2.71264	-1.07449
C	-12.70434	0.67569	3.25284	H	8.08450	-4.40163	-2.10228
C	-9.44382	-3.24035	-3.17094	H	8.81313	-5.61526	-4.15506
C	-1.30061	2.60058	2.88934	H	11.05323	-5.13599	-5.13331
C	-14.18518	-4.09206	3.95474	H	12.53493	-3.48465	-4.07994
C	-14.83911	-0.31890	-0.63036	H	12.37059	3.03817	-4.37449
C	-10.62130	0.58044	-3.18571	H	14.23210	4.53424	-4.94656

C	-15.16582	-4.39068	3.00374	H	15.98234	5.06269	-3.25540
C	-3.15099	0.45770	0.92739	H	15.83655	4.05480	-0.98047
C	-8.42399	0.44956	0.83432	H	13.99863	2.50809	-0.42552
C	1.43404	-3.24976	0.91098	H	8.30581	-1.67091	1.79090
C	-14.95986	3.29167	-2.80586	H	6.65193	-3.47236	1.62846
C	0.25266	1.71023	4.50800	H	7.38711	-5.83668	1.33784
C	-9.05540	4.19912	2.54684	H	9.81930	-6.35967	1.22716
C	3.50579	5.18435	0.82906	H	11.48558	-4.55989	1.44686
C	-6.52623	1.05950	2.38925	H	11.39293	-3.17246	4.30446
C	-9.46378	4.87595	3.69401	H	13.18092	-4.35512	5.50305
C	1.68485	-1.07602	2.89545	H	15.47990	-4.45128	4.54897
C	-10.71965	4.60463	4.24624	H	15.95424	-3.33234	2.37290
C	-8.12352	5.04117	-1.41868	H	14.17239	-2.10287	1.19703
C	2.80955	2.92609	0.29394	H	10.25303	3.32890	3.89021
C	0.72352	-4.36635	0.43677	H	8.11747	4.45365	4.35045
C	1.39438	-5.44898	-0.13407	H	6.25149	4.32748	2.69737
C	-5.10803	1.05930	2.42050	H	6.56396	3.04932	0.59164
C	-9.48708	5.33434	-1.35914	H	8.69659	1.88563	0.14464
C	-1.23318	-4.35892	4.34948	C	14.83906	0.31834	0.63082
C	-9.25345	-4.88902	2.15157	C	10.62116	-0.57988	3.18596
C	-8.65308	2.69367	-1.69580	C	15.16682	4.38925	-3.00426
C	-12.93126	-5.42309	-1.53309	C	3.15099	-0.45732	-0.92768
C	2.64864	5.17946	1.93129	C	8.42399	-0.44937	-0.83448
C	3.58444	4.05298	0.01319	C	-1.43402	3.25011	-0.91127
C	-1.74287	2.85471	5.25754	C	14.95921	-3.29195	2.80652
C	-13.40373	3.86177	-4.55963	C	-0.25265	-1.70989	-4.50830
C	-7.07542	0.60634	1.20041	C	9.05503	-4.19958	-2.54589
C	-2.10540	3.07379	3.92688	C	-3.50567	-5.18402	-0.82927
C	-0.37253	-3.81910	3.39321	C	6.52625	-1.05905	-2.38951
C	-16.09842	4.29718	1.05116	C	9.46346	-4.87684	-3.69279
C	2.78586	-5.43284	-0.24936	C	-1.68484	1.07638	-2.89574
C	1.87157	4.05385	2.21489	C	10.71943	-4.60584	-4.24497
C	-14.69333	3.91782	-4.02166	C	8.12236	-5.04027	1.41961
C	-2.41894	-3.69930	4.68128	C	-2.80948	-2.92574	-0.29420
C	-8.23910	-3.89062	-3.42873	C	-0.72351	4.36674	-0.43714
C	-8.04653	-4.40597	2.66015	C	-1.39437	5.44937	0.13369
C	-7.71071	3.71801	-1.58542	C	5.10805	-1.05880	-2.42079
C	2.83212	-3.24478	0.78782	C	9.48586	-5.33379	1.36024
C	3.50160	-4.32678	0.21161	C	1.23309	4.35923	-4.34985
H	-11.26442	-4.41667	1.57017	C	9.25474	4.88927	-2.15218
H	-9.36391	-5.94410	1.91464	C	8.65250	-2.69283	1.69623
H	-7.21076	-5.08215	2.82080	C	12.93144	5.42323	1.53231
H	-6.98658	-2.65692	3.35344	C	-2.64854	-5.17914	-1.93151
H	-8.86004	-1.12626	2.97187	C	-3.58434	-4.05264	-0.01342
H	-12.71032	4.55112	1.09288	C	1.74289	-2.85438	-5.25782
H	-14.71937	5.91721	0.68995	C	13.40296	-3.86155	4.56035
H	-16.98534	4.90190	0.88114	C	7.07543	-0.60606	-1.20060
H	-17.20423	2.48689	1.45181	C	2.10542	-3.07343	-3.92716
H	-15.20435	1.11318	1.81638	C	0.37246	3.81938	-3.39357
H	-9.55241	2.71261	1.07501	C	16.09842	-4.29730	-1.05034
H	-8.08495	4.40143	2.10317	C	-2.78585	5.43320	0.24904
H	-8.81348	5.61430	4.15644	C	-1.87150	-4.05351	-2.21514
H	-11.05342	5.13445	5.13479	C	14.69256	-3.91789	4.02240
H	-12.53505	3.48329	4.08105	C	2.41891	3.69969	-4.68158
H	-12.36971	-3.03942	4.37405	C	8.23919	3.89128	3.42803
H	-14.23090	-4.53601	4.94591	C	8.04767	4.40649	-2.66066
H	-15.98119	-5.06434	3.25479	C	11.62801	0.39654	-3.43772
H	-15.83583	-4.05583	0.98011	C	11.14366	-2.95677	-2.50099
H	-13.99823	-2.50863	0.42538	C	9.60719	-0.29381	-0.51509
H	-8.30614	1.67185	-1.79073	C	7.92246	3.04983	-2.96345

H	-6.65270	3.47369	-1.62802	C	14.03999	2.95460	-1.40923
H	-7.38846	5.83775	-1.33678	C	1.96542	-0.29117	-0.61898
H	-9.82078	6.36010	-1.22580	C	0.10450	-1.91735	-3.16581
H	-11.48661	4.55996	-1.44579	C	0.68658	2.61075	-2.74612
H	-11.39358	3.17300	-4.30385	C	10.42926	-4.31235	1.47926
H	-13.18179	4.35551	-5.50226	C	14.82813	-4.86646	-0.94394
H	-15.48077	4.45115	-4.54815	C	8.99461	2.17914	-2.75823
H	-15.95489	3.33184	-2.37222	C	7.36947	3.11010	1.31690
H	-14.17282	2.10254	-1.19653	C	10.02694	-2.97794	1.65682
H	-10.25299	-3.32824	-3.89072	C	13.68878	2.89496	2.43299
H	-8.11738	-4.45280	-4.35126	C	7.19476	3.82450	2.50007
H	-6.25136	-4.32685	-2.69821	C	11.44488	0.70661	3.27492
H	-6.56380	-3.04909	-0.59225	C	0.56292	-2.16758	-5.54582
H	-8.69648	-1.88557	-0.14496	C	11.55136	-3.65852	-3.65259
H	-11.06450	-4.51042	-0.99447	C	13.63887	0.18556	0.37417
H	-12.62736	-6.40572	-1.18158	C	10.32925	4.02160	-1.95167
H	-14.88027	-6.07770	-2.19034	C	12.04881	4.34803	1.42131
H	-15.54986	-3.81483	-2.98892	C	2.73239	2.49316	-4.05584
H	-14.01323	-1.91614	-2.76440	C	13.13976	3.22940	-3.63167
H	-3.02138	3.60707	3.68795	C	16.22190	-2.94543	-1.37580
H	-2.37503	3.21652	6.06436	C	4.51154	-0.60802	-1.25540
H	-0.26961	1.99146	6.57714	C	10.21526	2.65347	-2.25173
H	1.17472	1.19774	4.76155	C	-2.25841	-0.29220	-2.52309
H	-1.60879	2.75623	1.86335	C	-1.94153	-2.91306	-1.39888
H	0.54387	-4.34683	3.15027	C	13.94740	-2.60164	2.12902
H	-0.97401	-5.29588	4.83615	C	12.39483	-3.17770	3.88449
H	-3.08923	-4.12247	5.42530	C	1.87193	1.94908	-3.09812
H	-3.64916	-1.96493	4.30537	C	9.88976	-3.24637	-1.95122
H	-2.12899	-1.01424	2.61315	C	12.65518	-2.52835	2.66178
H	3.40988	-2.38872	1.11560	C	15.08717	3.82502	-1.73255
H	4.58355	-4.29339	0.11450	C	12.70401	-0.67656	-3.25273
H	3.30758	-6.27508	-0.69652	C	9.44388	3.24090	3.17041
H	0.82393	-6.30219	-0.49111	C	1.30063	-2.60021	-2.88962
H	-0.35756	-4.39898	0.51999	C	14.18622	4.09057	-3.95527
H	1.20689	4.07150	3.07144	H	4.52068	-1.38086	-3.27380
H	2.57838	6.05429	2.57293	C	16.22070	0.46892	0.92233
H	4.10433	6.06372	0.60528	C	16.75837	0.15985	2.19259
H	4.24168	4.04503	-0.85215	C	17.14493	0.93731	-0.03952
H	2.87622	2.05221	-0.34450	C	18.12041	0.32752	2.42260
H	-11.79566	-0.97128	4.35403	H	16.11062	-0.21784	2.97776
H	-10.63880	0.06599	3.50818	C	18.48774	1.06525	0.30173
H	-11.12909	-1.32006	-4.12387	H	16.80255	1.20609	-1.03417
H	-12.50556	-0.46903	-3.40599	H	18.53643	0.08895	3.40148
H	3.02246	0.19501	1.74800	H	19.20136	1.42722	-0.43855
H	2.72231	0.78078	3.38698	C	-16.22070	-0.46983	-0.92196
H	-12.77972	1.31519	4.13718	C	-16.75843	-0.16039	-2.19211
H	-13.68194	0.20803	3.09647	C	-17.14480	-0.93899	0.03963
H	-10.65370	1.13681	-4.12706	C	-18.12039	-0.32845	-2.42226
H	-9.57320	0.34405	-2.97523	H	-16.11077	0.21788	-2.97708
H	-7.14797	1.39194	3.21394	C	-18.48754	-1.06725	-0.30174
H	1.07769	-0.99627	3.80191	H	-16.80237	-1.20808	1.03418
H	2.48027	-1.79839	3.10757	H	-18.53645	-0.08960	-3.40105
H	-4.52066	1.38148	3.27346	H	-19.20107	-1.42981	0.43835
Ru	11.62102	-0.04786	-0.05484	N	-18.99587	-0.77379	-1.50829
P	11.24369	1.64415	1.67128	N	18.99601	0.77213	1.50840
P	12.28178	-1.69647	-1.74101	C	13.80091	-2.73020	-1.50189
P	11.64013	1.51023	-1.93827	C	15.08377	-2.16716	-1.59548
P	11.28689	-1.62453	1.78316	C	13.05802	2.63894	-2.35580
P	-0.51805	1.79139	-1.58788	C	14.19439	5.23988	2.09830
P	-0.95428	-1.37930	-1.74776	C	8.57807	2.45356	1.05769

S	5.76914	-0.16942	-0.08768	C	12.41409	3.06745	1.87014
C	14.56876	3.97306	2.54901	C	13.69008	-4.09044	-1.16758
C	9.62645	2.50372	1.98369				

Table S9. Cartesian Coordinates of $[\mathbf{2^H}]^+$.

P	6.34276	-1.65550	-1.98718	H	-2.25705	5.95996	-2.36891
P	6.34922	1.65722	-2.04173	H	-4.46674	6.40775	-1.23013
P	5.59529	-1.53674	1.78009	H	-6.13218	4.57460	-0.94980
P	5.39763	1.76099	1.60725	H	-8.60646	-2.52656	2.83080
P	-5.98368	-1.64818	-1.75844	H	-9.89623	-4.63859	2.66223
P	-6.15072	1.65812	-1.68457	H	-8.85735	-6.69626	1.63036
P	-5.77289	-1.75749	2.01579	H	-6.47863	-6.59673	0.78212
P	-5.67389	1.54950	2.11297	H	-5.16657	-4.49093	0.96760
C	4.97366	-2.70107	-2.75109	H	-5.16919	-3.96402	3.99175
S	0.05969	0.25973	0.23952	H	-3.11487	-4.63929	5.22258
C	5.15566	-3.26274	-4.04757	H	-0.92943	-3.43822	4.81668
C	4.15667	-4.06884	-4.63085	H	-0.84533	-1.53498	3.15861
C	2.96021	-4.33843	-3.92654	H	-2.90163	-0.86332	1.90920
C	2.77575	-3.79937	-2.63696	H	-5.77756	4.53204	1.67779
C	3.77604	-2.98527	-2.05262	H	-7.53896	6.25750	2.03901
C	7.67441	-2.97214	-1.77260	H	-9.78874	5.60503	2.98379
C	9.04539	-2.67504	-1.99321	H	-10.24612	3.18539	3.54917
C	10.03376	-3.67227	-1.83375	H	-8.50740	1.45383	3.16646
C	9.67028	-4.98113	-1.44757	H	-3.40579	2.59240	0.42104
C	8.30980	-5.28739	-1.22402	H	-1.39335	3.97721	0.92253
C	7.32064	-4.29234	-1.38580	H	-0.92612	4.73508	3.28680
C	4.91518	2.18352	-3.14129	H	-2.52857	4.10260	5.13551
C	4.90523	1.98448	-4.54785	H	-4.55176	2.75080	4.63657
C	3.81918	2.43293	-5.33502	H	1.28956	-0.35514	-3.19711
C	2.73119	3.09909	-4.73253	H	-1.34932	-0.38388	-3.12270
C	2.73346	3.31035	-3.33539	H	-7.61897	-0.66996	3.22822
C	3.81053	2.85052	-2.54802	H	-6.49599	-1.48032	4.38471
C	7.29593	3.27476	-1.82664	H	-4.76733	0.30439	4.03353
C	8.23819	3.41844	-0.77457	H	-6.31023	1.09009	4.49660
C	8.99882	4.60217	-0.64551	H	7.71076	-1.30060	-4.06168
C	8.82809	5.66294	-1.56194	H	6.06417	-0.61525	-4.18769
C	7.89326	5.53043	-2.61213	H	7.68825	1.29694	-4.12483
C	7.13361	4.34683	-2.74566	H	8.46710	0.65627	-2.64580
C	6.95022	-0.69477	-3.52082	H	4.23008	-1.10052	3.84210
C	7.50179	0.71269	-3.19499	H	5.80691	-0.24813	3.86648
C	4.31007	-2.91067	1.65339	H	3.98313	1.45901	3.64124
C	2.92245	-2.61884	1.73956	H	3.30589	0.60256	2.20783
C	1.96028	-3.64889	1.64197	H	-4.23025	-0.53996	-3.00485
C	2.36707	-4.98807	1.45120	H	6.40996	4.27049	-3.57485
C	3.74344	-5.28953	1.35994	H	2.57234	-1.58286	1.88207
C	4.70661	-4.26090	1.46144	H	0.88617	-3.39846	1.71800
C	6.99973	-2.45643	2.63145	H	1.61345	-5.79419	1.37829
C	6.84155	-2.89854	3.97639	H	4.07551	-6.33462	1.21542
C	7.87305	-3.60521	4.62814	H	5.77664	-4.52308	1.40156
C	9.07892	-3.89103	3.94663	H	5.90488	-2.70497	4.52854
C	9.24037	-3.46913	2.61116	H	7.73220	-3.93732	5.67373
C	8.20746	-2.75628	1.95624	H	9.88835	-4.44461	4.45841
C	4.42238	3.30977	1.18196	H	10.17712	-3.68959	2.06676
C	5.11237	4.49940	0.82094	H	8.35360	-2.42691	0.91786
C	4.40465	5.67255	0.48331	H	6.21497	4.52123	0.81109
C	2.99205	5.68139	0.50224	H	4.96404	6.58548	0.20691
C	2.29569	4.50794	0.86256	H	2.43637	6.60122	0.24132
C	3.00249	3.33034	1.19865	H	1.19086	4.50199	0.88675
C	6.63829	2.42772	2.86436	H	2.42435	2.43249	1.47036
C	7.93593	1.87019	2.98136	H	8.26009	1.08333	2.28309
C	8.82555	2.31982	3.98659	H	9.83539	1.87428	4.05707
C	8.42931	3.32774	4.88916	H	9.12575	3.68066	5.67267
C	7.13269	3.88384	4.78856	H	6.80893	4.67111	5.49486

C	6.24460	3.43840	3.78779
C	4.92625	-0.48662	3.22839
C	4.24115	0.81929	2.76626
C	-7.66046	-2.26330	-2.35157
C	-8.49304	-2.96456	-1.43972
C	-9.74015	-3.48276	-1.84934
C	-10.18727	-3.29899	-3.17674
C	-9.37267	-2.60078	-4.09277
C	-8.11785	-2.09135	-3.68590
C	-4.92319	-3.20447	-1.87755
C	-5.25286	-4.24056	-2.79347
C	-4.42101	-5.37391	-2.92895
C	-3.24610	-5.49236	-2.15389
C	-2.90901	-4.46894	-1.24155
C	-3.74161	-3.33532	-1.10284
C	-7.74582	2.61974	-1.96178
C	-8.69925	2.80291	-0.93241
C	-9.88114	3.54542	-1.17040
C	-10.12303	4.11337	-2.43762
C	-9.17243	3.94453	-3.47149
C	-7.99337	3.20878	-3.23533
C	-4.88899	3.04133	-1.90743
C	-3.64252	2.80138	-2.54410
C	-2.70285	3.84395	-2.70858
C	-2.99083	5.14325	-2.23588
C	-4.22649	5.39309	-1.59907
C	-5.16748	4.35234	-1.43702
C	-5.31578	-0.65613	-3.21634
C	-6.02031	0.71447	-3.33978
C	-6.78131	-3.33885	1.90874
C	-8.11833	-3.40680	2.38326
C	-8.85773	-4.60783	2.28337
C	-8.27717	-5.75785	1.70682
C	-6.94743	-5.70217	1.23257
C	-6.20684	-4.50528	1.33348
C	-4.18656	-2.36519	2.84284
C	-4.22570	-3.42691	3.79136
C	-3.06316	-3.81017	4.49231
C	-1.84043	-3.13686	4.26668
C	-1.79253	-2.07595	3.33870
C	-2.95683	-1.69333	2.63060
C	-7.00341	2.85753	2.39037
C	-6.75784	4.22075	2.07711
C	-7.75374	5.20091	2.28593
C	-9.01125	4.83685	2.81486
C	-9.26653	3.48428	3.13177
C	-8.27354	2.50223	2.91880
C	-4.12394	2.55089	2.48376
C	-3.22214	2.91631	1.45562
C	-2.07988	3.70048	1.74336
C	-1.82129	4.12528	3.06263
C	-2.71713	3.76995	4.09766
C	-3.86026	2.99539	3.81098
C	-6.54996	-0.84185	3.47317
H	-5.40120	-1.23914	-4.16145
H	-7.06841	0.57867	-3.68519
H	-5.51023	1.35500	-4.09324
H	-12.75569	-0.56410	1.38155
H	5.23784	3.88887	3.73051
H	-8.17061	-3.10748	-0.39738
H	-10.36867	-4.02724	-1.12060
H	-11.16772	-3.69936	-3.49571
H	-9.70742	-2.45191	-5.13631
H	-7.50369	-1.56939	-4.43819
H	-6.16636	-4.17387	-3.40901
H	-4.69704	-6.16990	-3.64561
H	-2.59680	-6.38153	-2.25944
H	-1.99395	-4.55121	-0.62579
H	-3.46516	-2.55053	-0.38181
H	-8.53093	2.35841	0.05848
H	-10.61507	3.67352	-0.35348
H	-11.04854	4.69008	-2.62304
H	-9.34790	4.39150	-4.46790
H	-7.26055	3.10780	-4.05579
H	-3.38843	1.79747	-2.92276
H	-1.74147	3.63532	-3.21400
C	-5.81158	0.49632	3.69863
C	11.88176	0.04669	0.29343
C	10.65329	0.05373	0.21535
C	9.26601	0.05963	0.12620
C	8.01945	0.06114	0.06717
C	3.92417	0.06609	-0.50521
C	2.68058	0.07680	-0.72728
C	1.31718	0.03729	-1.01640
C	0.69230	-0.19435	-2.28402
C	-0.70727	-0.20672	-2.24329
C	-1.26428	0.01862	-0.94329
C	-2.61058	0.04181	-0.57494
C	-3.82901	0.02642	-0.24088
C	-7.89629	-0.17674	0.49588
C	-9.11859	-0.28270	0.72050
C	-10.48263	-0.38847	0.96585
C	-11.68996	-0.48192	1.18841
Ru	5.92849	0.06087	-0.16769
Ru	-5.83133	-0.05208	0.15772
H	6.08757	-3.08600	-4.61357
H	4.31639	-4.49296	-5.63980
H	2.17706	-4.97179	-4.38372
H	1.84766	-4.00830	-2.07372
H	3.61433	-2.57332	-1.04604
H	9.36185	-1.66112	-2.28855
H	11.09507	-3.41916	-2.01439
H	10.44466	-5.76139	-1.32621
H	8.01122	-6.31026	-0.92737
H	6.26280	-4.55863	-1.21962
H	5.74678	1.49029	-5.06080
H	3.83382	2.26431	-6.42795
H	1.88514	3.45520	-5.34953
H	1.88919	3.83419	-2.85004
H	3.78766	3.01993	-1.46006
H	8.38468	2.60051	-0.05238
H	9.72722	4.69313	0.18182
H	9.42144	6.59061	-1.45831
H	7.75017	6.35382	-3.33671
H	12.96580	0.04329	0.36306

Table S10. Cartesian Coordinates of [$\mathbf{3^H}$]$^+$.

Ru	11.74005	0.00852	-0.04918	H	-8.21529	-4.54837	4.58426
Ru	0.00019	-0.00028	-0.00020	H	-6.42439	-4.63472	2.80152
P	11.40771	1.72906	-1.85194	H	-6.80561	-3.46824	0.59849
P	12.37497	-1.68359	1.70581	H	-8.93366	-2.21658	0.18697
P	11.85220	1.62327	1.87690	H	-11.37405	-4.65447	1.18957
P	11.35704	-1.61504	-1.93120	H	-13.00164	-6.51424	1.50752
P	-0.59952	1.87948	1.63302	H	-15.21531	-6.08894	2.64938
P	-0.89310	-1.39265	1.88856	H	-15.77382	-3.75863	3.44903
S	5.80975	-0.12581	-0.05479	H	-14.17239	-1.89191	3.10521
C	14.80885	3.95980	-2.94636	H	-3.21711	3.55229	-3.80648
C	9.78826	2.65727	-2.14576	H	-2.69261	2.96430	-6.20708
C	13.85218	-2.83113	1.47700	H	-0.60014	1.65465	-6.73906
C	15.18498	-2.36566	1.63042	H	0.94804	0.96997	-4.92911
C	13.34448	2.71998	2.25570	H	-1.69525	2.82164	-1.97185
C	14.49711	5.26200	-2.49827	H	0.66895	-4.49330	-3.15994
C	8.78390	2.72567	-1.15071	H	-0.85533	-5.52451	-4.83310
C	12.64229	3.12703	-2.12313	H	-3.02736	-4.39391	-5.45201
C	13.64888	-4.19232	1.12166	H	-3.63488	-2.19126	-4.37408
C	11.83547	0.49515	3.39345	H	-2.10821	-1.15119	-2.69222
C	11.15940	-2.88120	2.52089	H	3.55712	-2.44775	-1.19824
C	9.70253	-0.16321	0.40591	H	4.77528	-4.34710	-0.16472
C	8.22053	3.36551	3.11541	H	3.51361	-6.33417	0.74954
C	14.29782	3.04004	1.25913	H	0.99014	-6.37588	0.61192
C	1.95780	-0.20365	0.58679	H	-0.24278	-4.48024	-0.42578
C	0.25809	-1.83716	3.30073	H	1.14081	4.00371	-3.42913
C	0.62004	2.74982	2.77906	H	2.45693	6.08065	-3.04141
C	10.49453	-4.33708	-1.58799	H	3.91899	6.30375	-0.99442
C	14.74395	-5.06364	0.93404	H	4.05019	4.39464	0.65604
C	9.25265	2.43398	2.86020	H	2.73483	2.30835	0.27138
C	7.58032	3.43383	-1.38740	H	-12.03707	-1.07149	-4.32198
C	10.08000	-2.99548	-1.80876	H	-10.81530	-0.05878	-3.48729
C	13.89268	2.90030	-2.75738	H	-11.23121	-1.34908	4.32760
C	7.36687	4.08462	-2.61917	H	-12.60779	-0.45617	3.60497
C	11.54141	0.73366	-3.45428	H	3.05367	0.31194	-1.90558
C	0.84375	-1.91616	5.69181	H	2.64072	0.80033	-3.57544
C	11.50413	-3.52182	3.74612	H	-12.98741	1.25164	-4.10247
C	13.77937	0.16835	-0.46811	H	-13.88436	0.18536	-2.97738
C	10.59869	4.20721	1.86243	H	-10.63118	1.10209	4.28096
C	12.34068	4.44236	-1.67704	C	-2.35221	-3.93202	-4.70759
C	2.69225	2.70194	4.10404	C	-8.36719	-4.03408	3.61671
C	13.50945	3.27858	3.55600	C	-8.37185	-4.71856	-2.74175
C	16.28037	-3.23996	1.44782	C	-7.74627	3.77913	1.84520
C	4.55137	-0.39701	1.19253	C	2.97532	-3.30229	-0.81520
C	10.45932	2.84213	2.23312	C	-17.55947	-0.46259	1.24522
C	-2.23681	-0.31440	2.65808	C	3.67196	-4.38048	-0.22343
C	-1.83603	-2.99815	1.60276	H	-11.52966	-4.55931	-1.38493
C	14.02565	-2.68501	-2.26864	H	-9.69882	-6.19332	-1.81670
C	12.49221	-3.10795	-4.11864	H	-7.56413	-5.44663	-2.94421
C	1.83112	2.11238	3.14973	H	-7.29305	-3.02543	-3.61219
C	9.91597	-3.19746	1.92288	H	-9.09864	-1.38260	-3.15199
C	12.74012	-2.52881	-2.84041	H	-12.62576	4.58713	-0.99594
C	15.39898	3.87981	1.55521	H	-14.56119	6.11974	-0.65859
C	12.88409	-0.62521	3.18974	H	-16.92358	5.27449	-0.95703
C	9.56680	3.33106	-3.38169	H	-17.30971	2.85640	-1.57733
C	1.44295	-2.56627	3.01274	H	-15.38789	1.31308	-1.88476
C	14.60807	4.11122	3.85161	H	-9.62690	2.71926	-0.97660
C	14.99934	0.26196	-0.71906	H	-8.06415	4.34680	-2.05379
C	10.66744	-0.53624	-3.32471	H	-8.68417	5.44732	-4.23964

C	15.56073	4.41237	2.85012
C	3.17285	-0.31424	0.87873
C	8.49877	-0.26945	0.72766
C	-1.55860	3.31577	0.89861
C	15.04486	-3.38344	-2.96086
C	-0.03132	-1.51969	4.65469
C	9.03069	-4.11613	2.53823
C	-3.34003	-5.37776	1.16646
C	6.59402	-0.65506	2.38849
C	9.37637	-4.73097	3.75857
C	-1.75025	1.12311	2.96057
C	16.35591	0.36759	-0.99780
C	10.62087	-4.43233	4.36112
C	8.17062	-5.10763	-1.63339
C	-2.66821	-3.12977	0.45937
C	-0.85766	4.43488	0.37153
C	-1.55593	5.51289	-0.21425
C	5.16941	-0.66445	2.43033
C	9.54961	-5.38247	-1.49846
C	1.13900	4.56585	4.35832
C	9.56442	5.13570	2.11106
C	8.69016	-2.72927	-1.92484
C	13.25924	5.49902	-1.86066
C	-2.52058	-5.25363	2.30997
C	-3.41380	-4.30996	0.24401
C	2.01268	-2.64842	5.39406
C	13.50775	-3.80033	-4.80896
C	7.13696	-0.37670	1.11724
C	2.30623	-2.97692	4.05127
C	0.27701	3.98065	3.40588
C	16.06483	-4.59126	1.09963
C	-2.96607	5.48916	-0.29139
C	-1.77431	-4.07381	2.52890
C	14.79213	-3.93737	-4.23189
C	2.35259	3.93159	4.70700
C	8.36788	4.03376	-3.61766
C	8.37091	4.71852	2.74097
C	7.74577	-3.77772	-1.84583
C	-2.97512	3.30168	0.81473
C	17.55952	0.46146	-1.24542
C	-3.67177	4.37986	0.22295
H	11.52880	4.55960	1.38432
H	9.69761	6.19334	1.81563
H	7.56303	5.44647	2.94323
H	7.29242	3.02537	3.61176
H	9.09837	1.38281	3.15201
H	12.62544	-4.58701	0.99763
H	14.56084	-6.11980	0.66095
H	16.92325	-5.27447	0.95905
H	17.30942	-2.85612	1.57829
H	15.38763	-1.31263	1.88506
H	9.62738	-2.71957	0.97610
H	8.06428	-4.34691	2.05307
H	8.68309	-5.44621	4.23987
H	10.90912	-4.91599	5.31328
H	12.47996	-3.33029	4.22718
H	12.76911	3.08283	4.35216
H	14.71792	4.53151	4.86894
H	16.42309	5.06540	3.08184
H	16.13266	4.11265	0.76130
H	-10.91102	4.91813	-5.31183
H	-12.48148	3.33218	-4.22550
H	-12.77094	-3.08048	-4.35276
H	-14.71992	-4.52900	-4.86930
H	-16.42384	-5.06439	-3.08147
H	-16.13199	-4.11330	-0.76043
H	-14.19056	-2.62630	-0.24627
H	-8.32366	1.70131	2.07239
H	-6.67018	3.54971	1.95469
H	-7.43103	5.92841	1.57512
H	-9.89813	6.41981	1.32974
H	-11.56865	4.57826	1.49559
H	-11.49221	3.03904	4.58492
H	-13.29261	4.23848	5.80251
H	-15.58943	4.47850	4.77525
H	-16.04238	3.48791	2.49730
H	-14.24241	2.25083	1.28371
H	-10.33912	-3.32812	4.17042
C	-1.44269	2.56557	-3.01324
C	-14.60946	-4.10943	-3.85174
C	-14.99934	-0.26211	0.71902
C	-10.66636	0.53647	3.32446
C	-15.56141	-4.41142	-2.84984
C	-3.17273	0.31381	-0.87895
C	-8.49873	0.26946	-0.72759
C	1.55880	-3.31632	-0.89904
C	-15.04469	3.38264	2.96229
C	0.03171	1.51903	-4.65511
C	-9.03092	4.11646	-2.53844
C	3.34034	5.37721	-1.16702
C	-6.59403	0.65490	-2.38852
C	-9.37728	4.73199	-3.75823
C	1.75057	-1.12364	-2.96093
C	-16.35589	-0.36827	0.99765
C	-10.62225	4.43392	-4.36011
C	-8.17163	5.10890	1.63287
C	2.66831	3.12935	-0.45971
C	0.85784	-4.43541	-0.37194
C	1.55609	-5.51344	0.21382
C	-5.16937	0.66417	-2.43045
C	-9.55074	5.38326	1.49826
C	-1.13850	-4.56614	-4.35906
C	-9.56543	-5.13565	-2.11190
C	-8.69027	2.73035	1.92438
C	-13.25967	-5.49842	1.86129
C	2.52099	5.25299	-2.31059
C	3.41395	4.30952	-0.24442
C	-2.01229	2.64769	-5.39460
C	-13.50723	3.79941	4.81012
C	-7.13689	0.37660	-1.11728
C	-2.30592	2.97619	-4.05183
C	-0.27657	-3.98094	-3.40657
C	-16.06515	4.59136	-1.09791
C	2.96623	-5.48975	0.29094
C	1.77469	4.07316	-2.52945
C	-14.79178	3.93632	4.23340
C	-12.64203	-3.12661	2.12365
C	-13.64920	4.19247	-1.12011
C	-11.83555	-0.49439	-3.39316
C	-11.15987	2.88198	-2.52031

H	14.19136	2.62554	0.24692	C	-9.70246	0.16345	-0.40572
H	8.32395	-1.70011	-2.07294	C	-8.22121	-3.36550	-3.11589
H	6.66979	-3.54791	-1.95554	C	-14.29762	-3.04008	-1.25870
H	7.42973	-5.92689	-1.57580	C	-1.95768	0.20315	-0.58713
H	9.89659	-6.41914	-1.32983	C	-0.25778	1.83651	-3.30117
H	11.56780	-4.57817	-1.49532	C	-0.61978	-2.75026	-2.77953
H	11.49284	-3.03951	-4.58440	C	-10.49527	4.33754	1.58797
H	13.29329	-4.23961	-5.80129	C	-14.74428	5.06369	-0.93212
H	15.58981	-4.47983	-4.77343	C	-9.25313	-2.43381	-2.86043
H	16.04242	-3.48880	-2.49561	C	-7.58043	-3.43455	1.38607
H	14.24240	-2.25109	-1.28273	C	-10.08023	2.99606	1.80858
H	10.34020	3.32828	-4.17062	C	-13.89215	-2.89957	2.75832
H	8.21628	4.54812	-4.58522	C	-7.36660	-4.08526	2.61781
H	6.42462	4.63393	-2.80320	C	-11.54016	-0.73352	3.45450
H	6.80517	3.46729	-0.60014	C	-0.84332	1.91546	-5.69227
H	8.93331	2.21603	-0.18787	C	-11.50528	3.52328	-3.74499
H	11.37360	4.65462	-1.18958	C	-13.77935	-0.16824	0.46826
H	13.00083	6.51478	-1.50701	C	-10.59950	-4.20700	-1.86302
H	15.21501	6.09000	-2.64807	C	-12.34090	-4.44199	1.67740
H	15.77436	3.75982	-3.44749	C	-2.69205	-2.70253	-4.10442
H	14.17328	1.89271	-3.10418	C	-13.51074	-3.27687	-3.55625
H	3.21739	-3.55304	3.80587	C	-16.28066	3.24020	-1.44668
H	2.69304	-2.96506	6.20650	C	-4.55132	0.39667	-1.19273
H	0.60063	-1.65536	6.73861	C	-10.45986	-2.84187	-2.23340
H	-0.94763	-0.97062	4.92875	C	2.23713	0.31385	-2.65840
H	1.69545	-2.82235	1.97133	C	1.83626	2.99761	-1.60318
H	-0.66842	4.49312	3.15914	C	-14.02551	2.68458	2.26967
H	0.85597	5.52435	4.83218	C	-12.49171	3.10739	4.11940
H	3.02778	4.39348	5.45138	C	-1.83098	-2.11297	-3.15005
H	3.63498	2.19055	4.37384	C	-9.91601	3.19768	-1.92295
H	2.10821	1.15047	2.69207	C	-12.73981	2.52849	2.84111
H	-3.55690	2.44712	1.19775	C	-15.39886	-3.87978	-1.55465
H	-4.77509	4.34645	0.16422	C	-12.88421	0.62592	-3.18939
H	-3.51347	6.33357	-0.74999	C	-9.56605	-3.33115	3.38117
H	-0.99000	6.37536	-0.61231	H	-9.61930	0.26101	3.06495
H	0.24295	4.47974	0.42539	H	-7.23485	0.84957	-3.26547
H	-1.14036	-4.00443	3.42852	H	1.14616	-1.13919	-3.89331
H	-2.45642	-6.08138	3.04069	H	2.60994	-1.80988	-3.13170
H	-3.91864	-6.30432	0.99379	H	-4.58256	0.86506	-3.34319
H	-4.05012	-4.39500	-0.65641	H	-18.62009	-0.54518	1.46334
H	-2.73485	-2.30866	-0.27159	H	18.62022	0.54378	-1.46324
H	12.03720	1.07248	4.32208	P	-11.40726	-1.72890	1.85206
H	10.81524	0.05955	3.48789	P	-12.37519	1.68406	-1.70523
H	11.23299	1.34925	-4.32755	P	-11.85246	-1.62280	-1.87686
H	12.60911	0.45623	-3.60414	P	-11.35679	1.61519	1.93131
H	-3.05340	-0.31251	1.90531	P	0.59975	-1.87997	-1.63342
H	-2.64032	-0.80087	3.57516	P	0.89338	1.39209	-1.88895
H	12.98747	-1.25072	4.10294	S	-5.80963	0.12560	0.05466
H	13.88421	-0.18470	2.97747	C	-14.80853	-3.95884	2.94757
H	10.63278	-1.10188	-4.28122	C	-9.78789	-2.65744	2.14526
H	9.62025	-0.26071	-3.06580	C	-13.85247	2.83142	-1.47602
H	7.23481	-0.84969	3.26546	C	-15.18526	2.36600	-1.62965
H	-1.14577	1.13866	3.89290	C	-13.34497	-2.71917	-2.25567
H	-2.60962	1.80933	3.13141	C	-14.49726	-5.26111	2.49935
H	4.58254	-0.86542	3.34302	C	-8.78396	-2.72616	1.14980
Ru	-11.74005	-0.00818	0.04944				

Tabale S11. Cartesian coordinates of **5.**

Ru	0.00001	0.00002	0.00003	C	2.56111	2.40295	2.13423
P	0.21254	1.67493	-1.78374	C	3.30835	-0.01171	-0.14806
P	0.09677	-1.53536	-1.87729	C	8.31359	-0.35186	-1.72627
S	-5.54644	0.35628	1.73307	C	-1.51866	-2.96401	1.55100
C	-9.32632	0.48977	2.37865	C	1.53465	1.76400	4.22301
C	-2.07393	0.01041	0.06865	C	-2.20969	5.68899	1.59848
C	-1.44447	-1.90924	-2.82871	C	6.98834	0.09345	0.36417
C	-1.16945	2.68141	-2.51742	C	-0.73436	-0.76191	3.35586
C	-2.70856	-2.09626	-4.90324	C	-2.08934	3.33780	1.03173
C	-3.53104	2.92004	-3.04753	C	-1.19746	-4.17451	0.91206
C	-4.71145	0.02689	0.21094	C	-2.17734	-5.14159	0.68393
C	1.20601	-0.67190	-3.10466	C	5.64436	0.18706	0.79477
C	0.90435	-3.20211	-1.77390	C	1.94172	-4.49807	3.94208
C	-2.49413	2.23479	-2.40845	C	-1.03450	5.56331	2.34118
C	-2.56113	-2.40299	-2.13393	C	-2.73614	4.57170	0.94717
C	-3.30833	0.01183	0.14807	C	3.80741	2.59371	4.20252
C	-8.31375	0.35195	1.72578	C	7.12825	-0.19273	-0.98102
C	1.51881	2.96393	-1.55110	C	3.72753	2.75121	2.81713
C	-1.53480	-1.76412	-4.22281	C	0.90476	-3.81769	3.30316
C	2.20965	-5.68901	-1.59827	C	-3.49589	-4.91571	1.08475
C	-6.98828	-0.09297	-0.36461	C	-0.38544	4.32998	2.43032
C	0.73427	0.76187	-3.35583	C	3.25976	-4.05441	3.81248
C	2.08923	-3.33783	-1.03146	C	-2.84753	-2.74772	1.94574
C	1.19775	4.17448	-0.91219	C	-3.82761	-3.71586	1.71564
C	2.17772	5.14151	-0.68417	H	4.57516	3.13796	2.25848
C	-5.64426	-0.18657	-0.79508	H	4.71877	2.85854	4.73232
C	-1.94173	4.49797	-3.94218	H	2.75739	1.97124	5.98207
C	1.03452	-5.56331	-2.34107	H	0.68893	1.40040	4.79702
C	2.73603	-4.57173	-0.94689	H	2.52275	2.51298	1.05799
C	-3.80755	-2.59386	-4.20214	H	-0.11173	-4.17991	3.41533
C	-7.12832	0.19292	0.98063	H	1.71690	-5.37643	4.54178
C	-3.72758	-2.75130	-2.81675	H	4.06695	-4.58866	4.30708
C	-0.90473	3.81761	-3.30329	H	4.55129	-2.56168	2.93842
C	3.49622	4.91552	-1.08509	H	2.71667	-1.35756	1.81332
C	0.38546	-4.32998	-2.43023	H	-3.13703	-1.81745	2.42061
C	-3.25977	4.05442	-3.81236	H	-4.85034	-3.52332	2.02769
C	2.84764	2.74754	-1.94593	H	-4.25861	-5.66951	0.90789
C	3.82780	3.71561	-1.71595	H	-1.90509	-6.07090	0.19094
H	-4.57517	-3.13803	-2.25803	H	-0.17735	-4.37173	0.60048
H	-4.71894	-2.85873	-4.73187	H	0.52871	4.25298	3.00825
H	-2.75765	-1.97147	-5.98178	H	-0.61749	6.42607	2.85453
H	-0.68912	-1.40054	-4.79688	H	-2.71181	6.65039	1.52822
H	-2.52269	-2.51297	-1.05769	H	-3.64853	4.65620	0.36340
H	0.11177	4.17974	-3.41564	H	-2.50264	2.47638	0.51929
H	-1.71692	5.37624	-4.54201	H	-2.19143	0.68087	2.63209
H	-4.06700	4.58865	-4.30694	H	-1.28160	1.23796	4.03971
H	-4.55130	2.56187	-2.93797	H	7.84508	0.23156	1.01475
H	-2.71662	1.35778	-1.81294	H	0.15807	-0.75917	3.98838
H	3.13703	1.81723	-2.42079	H	-1.49472	-1.34805	3.88272
H	4.85049	3.52299	-2.02807	H	5.34571	0.41621	1.81107
H	4.25900	5.66927	-0.90833	H	-10.22148	0.61177	2.94377
H	1.90557	6.07087	-0.19122	H	10.22118	-0.61189	-2.94445
H	0.17768	4.37179	-0.60054	S	5.54629	-0.35634	-1.73325
H	-0.52865	-4.25298	-3.00823	C	9.32609	-0.48979	-2.37923
H	0.61755	-6.42607	-2.85446	C	2.07395	-0.01034	-0.06859
H	2.71177	-6.65040	-1.52799	C	1.44440	1.90918	2.82892
H	3.64838	-4.65624	-0.36305	C	1.16950	-2.68137	2.51748
H	2.50248	-2.47642	-0.51897	C	2.70837	2.09609	4.90354

H	2.19138	-0.68090	-2.63209	C	3.53104	-2.91993	3.04782
H	1.28149	-1.23803	-4.03966	C	4.71146	-0.02668	-0.21109
H	-7.84495	-0.23091	-1.01531	C	-1.20608	0.67186	3.10470
H	-0.15822	0.75910	-3.98827	C	-0.90438	3.20209	1.77405
H	1.49457	1.34799	-3.88279	C	2.49417	-2.23466	2.40870
H	-5.34550	-0.41554	-1.81138	P	-0.09679	1.53534	1.87740
P	-0.21250	-1.67492	1.78377				

VI. References

[S1] A. B. Pangborn, M. A. Giardello, R. H. Grubbs, R. K. Rosen, F. J. Timmers, *Organometallics,* 1996, **15**, 1518-1520.

[S2] S. Rigaut, J. Perruchon, L. Le Pichon, D. Touchard, P. Dixneuf. *J. Organomet. Chem.* 2003, **670**, 37-44.

[S3] D. Touchard, P. Haquette, S. Guesmi, L. Le Pichon, A. Daridor, L. Toupet, P. H. Dixneuf, *Organometallics* 1997, **16**, 3640-3648.

[S4] T. X. Neenan, G. M. Whitesides, *J. Org. Chem.* 1988, **53**, 2489-2496.

[S5] K. Sugimoto, H. Idei, Y. Tanaka, M. Akita, *J. Organomet. Chem.*, 2017, **847**, 121-131.

[S6] P. de Frémont, N. M. Scott, E. D. Stevens, S. P. Nolan, *Organometallics*, 2005, **24**, 2411-2418.

[S7] *APEX 2,* version 2011.11-3, Bruker AXS Inc., Madison, Wisconsin, USA.

[S8] Rigaku Oxford Diffraction, CrysAlisPro, Rigaku Corporation, Tokyo, Japan. 2015.

[S9] O. V. Dolomanov, L. J. Bourhis, R. J. Gildea, J. A. K. Howard, H. Puschmann, *J. Appl. Cryst.*, 2009, **42**, 339-341.

[S10] G. M. Sheldrich, *Acta Cryst.* 2015, **A71**, 3.

[S11] G. M. Sheldrich, *Acta Cryst.* 2015, **C71**, 3.

[S12] M. Parthey, J. B. G. Gluyas, M. A. Fox, P. J. Low, M. Kaupp, *Chem. Eur. J.* 2014, **20**, 6895 – 6908.

[S13] M. Renz, K. Theilacker, C. Lambert, M. Kaupp, *J. Am. Chem. Soc.* 2009, **131**, 16292–16302.

[S14] Gaussian 16, Revision B.01, M. J. Frisch, G. W. Trucks, H. B. Schlegel, G. E. Scuseria, M. A. Robb, J. R. Cheeseman, G. Scalmani, V. Barone, G. A. Petersson, H. Nakatsuji, X. Li, M. Caricato, A. V. Marenich, J. Bloino, B. G. Janesko, R. Gomperts, B. Mennucci, H. P. Hratchian, J. V. Ortiz, A. F. Izmaylov, J. L. Sonnenberg, D. Williams-Young, F. Ding, F. Lipparini, F. Egidi, J. Goings, B. Peng, A. Petrone, T. Henderson, D. Ranasinghe, V. G. Zakrzewski, J. Gao, N. Rega, G. Zheng, W. Liang, M. Hada, M. Ehara, K. Toyota, R. Fukuda, J. Hasegawa, M. Ishida, T. Nakajima, Y. Honda, O. Kitao, H. Nakai, T. Vreven, K. Throssell, J. A. Montgomery, Jr., J. E. Peralta, F. Ogliaro, M. J. Bearpark, J. J. Heyd, E. N. Brothers, K. N. Kudin, V. N. Staroverov, T. A. Keith, R. Kobayashi, J. Normand, K. Raghavachari, A. P. Rendell, J. C. Burant, S. S. Iyengar, J. Tomasi, M. Cossi, J. M. Millam, M. Klene, C. Adamo, R. Cammi, J. W. Ochterski, R. L. Martin, K. Morokuma, O. Farkas, J. B. Foresman, and D. J. Fox, Gaussian, Inc., Wallingford CT, 2016.

[S15] Gaussian 09, Revision D.01, M. J. Frisch, G. W. Trucks, H. B. Schlegel, G. E. Scuseria, M. A. Robb, J. R. Cheeseman, G. Scalmani, V. Barone, B. Mennucci, G. A. Petersson, H. Nakatsuji, M. Caricato, X. Li, H. P. Hratchian, A. F. Izmaylov, J. Bloino, G. Zheng, J. L. Sonnenberg, M. Hada, M. Ehara, K. Toyota, R. Fukuda, J. Hasegawa, M. Ishida, T. Nakajima, Y. Honda, O. Kitao, H. Nakai, T. Vreven, J. A. Montgomery, J. E. Peralta Jr., F. Ogliaro, M. Bearpark, J. J. Heyd, E. Brothers, K. N. Kudin, V. N. Staroverov, R. Kobayashi, J. Normand, K. Raghavachari, A. Rendell, J. C. Burant, S. S. Iyengar, J. Tomasi, M. Cossi, N. Rega, J. M. Millam, M. Klene, J. E. Knox, J. B. Cross, V. Bakken, C. Adamo, J. Jaramillo,

R. Gomperts, R. E. Stratmann, O. Yazyev, A. J. Austin, R. Cammi, C. Pomelli, J. W. Ochterski, R. L. Martin, K. Morokuma, V. G. Zakrzewski, G. A. Voth, P. Salvador, J. J. Dannenberg, S. Dapprich, A. D. Daniels, Ö Farkas, J. B. Foresman, J. V. Ortiz, J. Cioslowski, and D. J. Fox. Gaussian, Inc., Wallingford CT, 2009.

[S16] T. Tada, M. Kondo and K. Yoshizawa, *J. Chem. Phys.*, 2004, **121**, 8050–8057.

[S17] K. Sugimoto, Y. Tanaka, S. Fujii, T. Tada, M. Kiguchi, M. Akita, *Chem. Commun.*, 2016, **52**, 5796-5799.
